# Nanoscale junctional membrane curvatures recruit BIN1 and SNX9 for endothelial collective migration

**DOI:** 10.1083/jcb.202509207

**Published:** 2026-07-27

**Authors:** Vera Janssen, Hannah de Kraker, Jesse S. Aaron, Annett de Haan, Iris de Heer, Satya Khuon, Jason Da Silva, Amber J.M. Driessen, Teng-Leong Chew, Josephine M.E. Tan, Anne K. Lagendijk, Ana Angulo-Urarte, Stephan Huveneers

**Affiliations:** 1 Department of Medical Biochemistry, Amsterdam UMC, Amsterdam Cardiovascular Sciences, University of Amsterdam, Amsterdam, the Netherlands; 2 Advanced Imaging Center, Howard Hughes Medical Institute (HHMI), Janelia Research Campus, Ashburn, VA, USA; 3 Centre for Cell Biology of Chronic Disease, Institute for Molecular Bioscience, The University of Queensland, Brisbane, Australia; 4 Department of Physiology, Amsterdam UMC, VU University, Amsterdam Cardiovascular Sciences, Amsterdam, the Netherlands; 5 Endothelial Pathobiology and Microenvironment, Josep Carreras Leukaemia Research Institute, Barcelona, Spain

## Abstract

Collective endothelial migration during vascular development relies on dynamic cell–cell contacts, yet how the junctions between leader and follower cells are organized remains poorly understood. Using Cryo-SIM followed by FIB-SEM, we investigated the nanoscale 3D ultrastructure of asymmetric adherens junctions (AAJs), revealing a wide spectrum of membrane folds at the contact interface between migrating endothelial cells. By performing a junction localization screen, we identified a variety of membrane curvature-sensing BAR domain proteins selectively enriched at AAJs. Among these, BIN1 and SNX9 emerged as novel regulators of front-to-rear polarity in follower cells during endothelial collective migration. The spatiotemporal recruitment of these individual BAR proteins associated with distinct phases of AAJ remodeling. Furthermore, depletion of SNX9 disturbed VE-cadherin dynamics, endothelial cell directionality, and sheet migration in the common cardinal vein of zebrafish. These findings highlight junctional nanoscale membrane curvatures as hubs for the spatiotemporal recruitment of a repertoire of BAR proteins to remodel AAJs and guide collective endothelial migration during vascular development.

## Teaser

Janssen et al. show that nanoscale membrane curvatures at asymmetric endothelial cell–cell junctions serve as a scaffold to recruit BAR proteins, including BIN1 and SNX9, during collective migration. These curvature-sensitive proteins coordinate junction remodeling, cell polarity, and directional movement, identifying membrane topology as a key organizer of endothelial guidance during vascular development.

## Introduction

Coordinated cell migration depends on dynamic intercellular junctions, which not only hold tissues together but also enable cells to move collectively. The regulation of junctions is essential for shaping tissues during embryonic development, tissue regeneration, and disease progression (Friedl and Mayor, 2017; Khalil and de Rooij, 2019). In addition to mediating adhesion between neighboring cells, junctions serve as hubs for intercellular communication via force transmission, coordination of proliferation, exchange of molecules, and signal transduction (Angulo-Urarte et al., 2020; Collins and Nelson, 2015; Friedl and Gilmour, 2009; Friedl and Mayor, 2017; Grimsley-Myers et al., 2020; Gumbiner and Kim, 2014; Han et al., 2016; Kim et al., 2011; Trepat et al., 2009; Yap et al., 2018). During vascular development, the plasticity of endothelial junctions supports sprouting, lumen formation, and collective cell rearrangements, all while safeguarding vessel barrier integrity (Arima et al., 2011; Bentley et al., 2014; Grimsley-Myers et al., 2020; Szymborska and Gerhardt, 2018).

Endothelial junctions are generated by VE-cadherin–based adherens junctions (AJs). Genetic deletion of VE-cadherin is embryonically lethal in mice due to defects in the morphogenesis of the vascular network (Carmeliet and Collen, 2000; Carmeliet et al., 1999; Gory-Faure et al., 1999). VE-cadherin forms homodimers through its extracellular domain. Its cytoplasmic domain associates with the actomyosin network (Huveneers et al., 2012), which mechanically couples ECs to coordinate collective behavior. At the front of collectively migrating ECs, leader cells are polarized and primarily move in response to mechanical and growth factor cues. The coordination of follower cells, in turn, depends on junctional mechanotransduction. Disrupting VE-cadherin’s function, or its cytoskeletal linkage, impairs directional migration and front-to-rear polarity (Carvalho et al., 2019; Helker et al., 2013; Vitorino and Meyer, 2008).

The junctional interface between leader and follower cells is subject to forces arising from differences in their cytoskeletal organization. Rho-mediated actomyosin contractility at the rear of leader cells results in pulling forces at the front of adherent follower cells. Meanwhile, at the front of the followers, actin polymerization promotes cell protrusions (Efimova and Svitkina, 2018; Janssen and Huveneers, 2024; Malinova and Huveneers, 2018). The integration of these pushing and pulling forces induces the formation of tubular VE-cadherin–based junctions that extend from leader into follower ECs, which are termed asymmetric AJs (AAJs) or cadherin fingers (Brevier et al., 2008; Dorland et al., 2016; Efimova and Svitkina, 2018; Hayer et al., 2016; Malinova et al., 2021). The formation of AAJs is necessary to guide follower cells and ensure collective endothelial migration and angiogenesis (Hayer et al., 2016; Malinova et al., 2021).

We have previously shown that protein kinase C and casein kinase substrate in neurons 2 (PACSIN2), a Bin/Amphiphysin/Rvs (BAR) protein, is recruited to the trailing end of AAJs in follower cells. PACSIN2 protects AAJs from fast internalization (Dorland et al., 2016). In general, BAR domain–containing proteins sense and induce membrane curvature (Simunovic et al., 2019; Simunovic et al., 2015), and contain auxiliary domains to mediate protein–protein interactions, as well as regulation of Rho GTPase signaling. The presence of the F-BAR protein PACSIN2 at endothelial AAJs and its importance for EC migration highlight important questions about how the plasma membrane architecture between leader and follower cells is organized, and how this organization supports junction remodeling. To address this, we mapped AAJs in three dimensions at isotropic nanometer resolution using cryo-structured illumination microscopy paired with focused-ion beam scanning electron microscopy (Cryo-SIM with FIB-SEM). These experiments revealed elaborate membrane folds at the contact interface between migrating ECs, displaying a spectrum of curvature sizes along the junctions. This diversity in junctional membrane topology suggested that BAR domain proteins beyond PACSIN2 contribute to AAJ dynamics. By combining a junction localization screen with loss-of-function migration assays, we identified bridging integrator 1 (BIN1) and sorting nexin-9 (SNX9) as novel junction regulators. We show that recruitment of the membrane curvature sensor SNX9 and its interactor dynamin-2 follows after PACSIN2 recruitment to trigger VE-cadherin internalization from AAJs. Depletion of SNX9 in cultured ECs and zebrafish embryos demonstrates that SNX9-mediated junction remodeling is important for directed collective migration in the developing vasculature. This study reveals a mechanism by which spatiotemporal regulation of nanoscale membrane topology controls endothelial junction plasticity during vascular development.

## Results

### Ultrastructural characterization of the asymmetric junctional membrane

**Figure 1.**
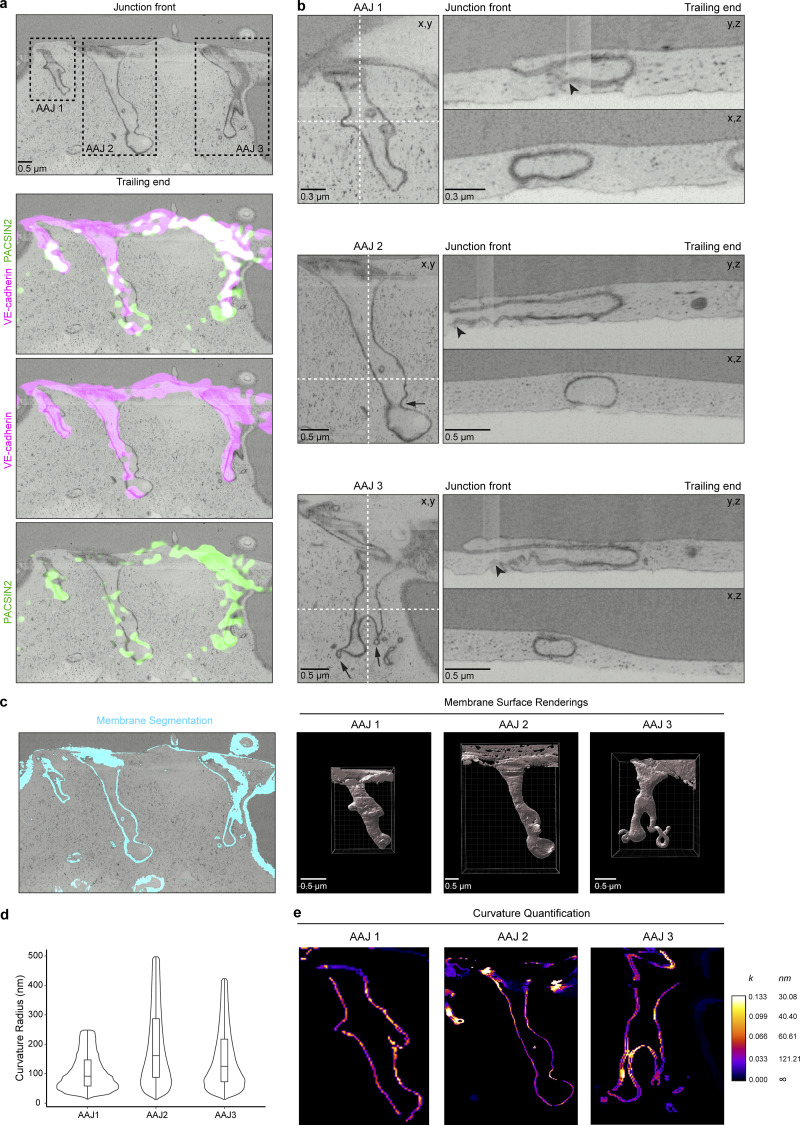
**Ultrastructural characterization of the asymmetric junctional membrane with Cryo-SIM and FIB-SEM. (a)** Representative FIB-SEM slice of three AAJs in BOECs acquired at 4 nm isotropic voxel size overlaid with Cryo-SIM–acquired localization of VE-cadherin–mScarlet (magenta) and PACSIN2-GFP (green). **(b)** Magnification of FIB-SEM images of individual AAJs with orthoslices in XY, YZ, and XZ, with dotted lines indicating axes along which the volume was sliced. Arrowheads indicate narrow concave curvature at the junction front; arrows indicate ring-shaped structures and spiral-shaped membranes. **(c)** Left image is a representative FIB-SEM slice of three endothelial AAJs acquired at 4 nm isotropic voxel size overlaid with plasma membrane segmentations in cyan. Right images show plasma membrane surface rendering of individual AAJs. See Video 3. **(d)** Violin and box plots depicting the average membrane curvature per pixel from a 60 nm search radius from the follower cell side’s perspective. **(e)** Heatmaps of subjunctional curvature values *(k)* with radius of curvature (nm) within AAJs. *k* = 0–0.133, radius = ∼30-infinity nm. Scale bars = 0.3 and 0.5 μm.

**Figure S1.**
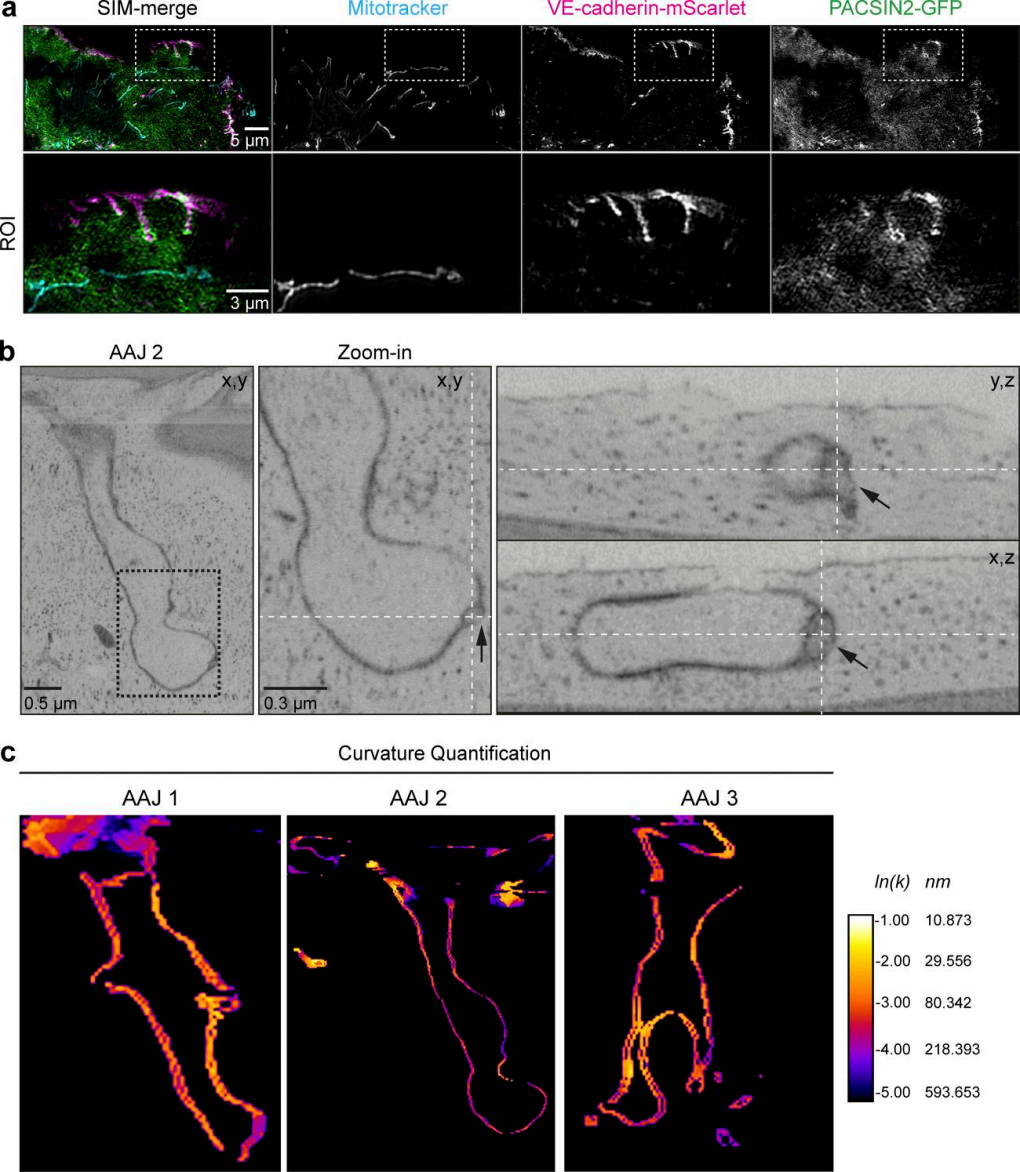
**Cryo-SIM, FIB-SEM, and curvatures of endothelial AAJs. (a)** Aligned SIM images of BOECs labeled for MitoTracker (cyan) and overexpressing VE-cadherin–mScarlet (magenta) and PACSIN2-GFP (green). ROI indicates region containing AAJs 1–3. **(b)** FIB-SEM orthoslices of AAJ2 to indicate a vesicle-like structure that extends with a single membrane from the double junctional membrane (black arrow). **(c)** Heatmaps of log-transformed subjunctional curvature values *(k)* with radius of curvature (nm) within AAJs. *ln*(*k)* = −1 to −5, radius = ∼10–600 nm. Scale bars = 5, 3, 0.5, and 0.3 μm.

Through conventional fluorescence microscopy, AAJs appear as linear extensions from leader toward follower cells. Images of these junctions with electron microscopy, and the notion that curvature-sensing proteins are recruited, indicate that the AAJs are tubular structures (Brevier et al., 2008; Dorland et al., 2016; Hayer et al., 2016). However, a comprehensive understanding of the intercellular plasma membrane topology at AAJs is currently lacking. To visualize the ultrastructural organization of VE-cadherin–based AAJs in ECs with nanoscale isotropic resolution, we paired Cryo-SIM with FIB-SEM. First, Cryo-SIM was performed on cord blood outgrowth endothelial cells (BOECs) expressing PACSIN2-GFP and VE-cadherin–mScarlet. Subsequently, PACSIN2-positive AAJs were further processed with FIB-SEM following a previously published workflow (Bharathan et al., 2023; Hoffman et al., 2020). Cryo-SIM data were registered onto corresponding 3D electron microscopy volumes using mitochondria as landmarks (Fig. 1 a; Fig. S1 a; and Videos 1 and 2). 2D sections and 3D reconstructions of the FIB-SEM datasets, at 4 × 4 × 4 nm voxel size, revealed that AAJs were fully engulfed by follower cells, from which basal and apical membrane lamellae enclosed the junctional structure (Fig. 1 b). Along most of their length, AAJs were composed of a double membrane from leader and follower cells, except in regions on the internalizing side of the AAJs where single membrane structures could be discerned that bud off from the junctional interface (Fig. S1 b). The most striking feature of AAJs was that their plasma membrane exhibited nonuniform curvatures (Fig. 1 b). This included concave and convex curvatures along the junction length, with narrow curvatures closer to the junction front as well as at the trailing end (Fig. 1 b). The trailing ends of AAJs were associated with ring-shaped structures, resembling the necks of budding endocytic vesicles and the formation of spiral-shaped membranes (Fig. 1, b and c; AAJ 2 and 3). To characterize the membrane curvature landscape at AAJs, we generated local Gaussian curvature maps from 3D reconstructions of plasma membrane segmentations of the FIB-SEM dataset (Fig. 1 c and Video 3). These analyses revealed local curvatures ranging from ∼12 to 500 nm within individual AAJs, calculated from the follower cell side’s perspective (Fig. 1 d and Fig. S1 c). Notably, the FIB-SEM resolution and analysis revealed a previously unknown breadth range within the narrow curvature spectrum (Fig. 1 e). These data demonstrate that AAJs constitute highly folded membrane landscapes extending deep into follower cells.

**Video 1.** **FIB-SEM overview of BOECs.** Overview through Z of FIB-SEM dataset at 4 × 4 × 4 nm isotropic resolution of BOECs and zoom-in of region with AAJ 1–4. Black boxes indicate AAJ ROIs. Scale bars = 5 and 1 μm.

**Video 2.** **Traverse across the z-axis EM + SIM overlay of AAJ 1–3.** FIB-SEM dataset of AAJ 1–3 through Z, overlaid with PACSIN2 (green) and VE-cadherin (magenta) signal acquired by SIM. Scale bar = 0.5 μm.

**Video 3.** **3D membrane renderings of AAJ 1–3.** Rotated view of 3D plasma membrane renderings of AAJ 1–3 from the segmented FIB-SEM dataset.

### Multiple BAR proteins are recruited to AAJs

**Figure 2.**
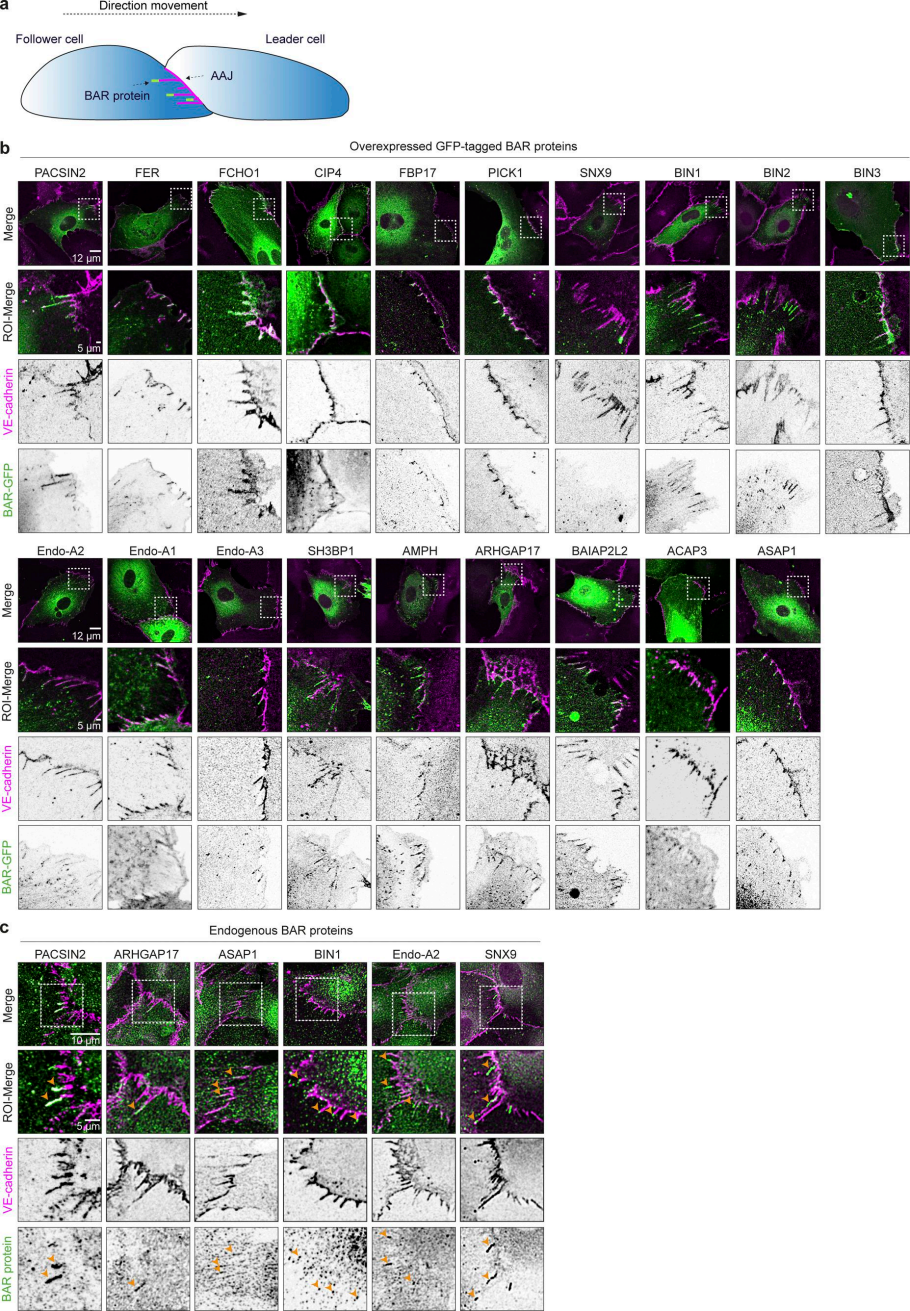
**Multiple BAR proteins are recruited to AAJs. (a)** Schematic representation of the AAJs between migrating leader and follower endothelial cells. **(b)** Representative confocal images from three independent experiments of HUVECs expressing GFP-tagged BAR proteins (green) immunostained for VE-cadherin (magenta). White squares indicate ROIs at AAJs, for which magnifications and single channels are shown below. **(c)** Representative confocal images of HUVECs immunostained for indicated endogenous BAR proteins (green) and VE-cadherin (magenta). White squares indicate ROIs, for which magnifications and single channels are shown below. Orange arrowheads indicate junctional recruited BAR proteins. Scale bars = 12, 10 and 5 μm.

**Figure S2.**
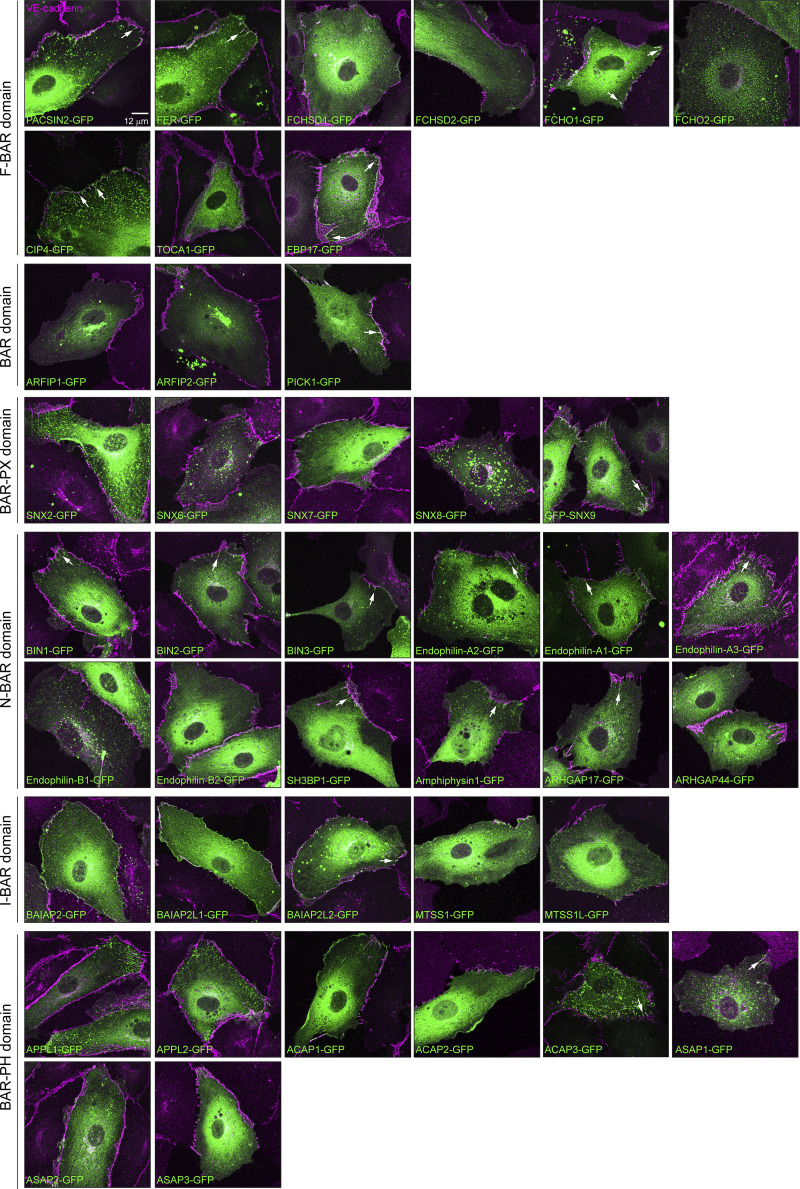
**Localization of BAR-GFP proteins in endothelial cells.** Representative confocal microscopy images of transfected HUVECs expressing GFP-tagged BAR domain–containing protein (green) immunostained for VE-cadherin (magenta). White arrows highlight asymmetric AJs that are positive for the indicated BAR protein. Scale bar = 12 μm.

**Figure S3.**
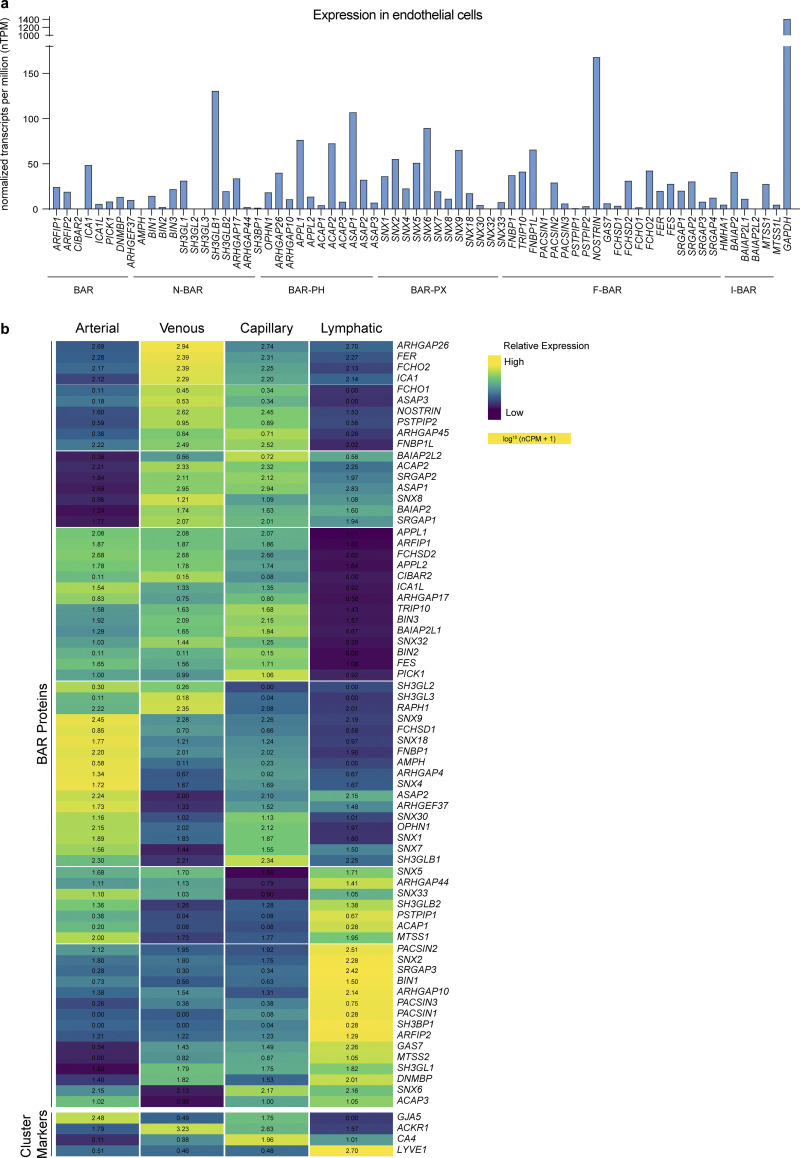
**BAR protein expression in endothelial cells. (a)** Number of normalized transcripts per million (nTPM) in endothelial cells for all known genes encoding human BAR domain proteins recorded in the RNA single cell type dataset from the Human Protein Atlas (summarized expression levels per gene in 81 cell types from 31 datasets). Genes are ordered based on the structure of the BAR domain, as indicated below the graph. GAPDH is displayed as a comparison. **(b)** Hierarchically clustered expression levels of genes encoding human BAR domain proteins across arterial, venous, capillary, and lymphatic endothelial cell types. Human vasculature scRNA-seq data were obtained from the publicly available Tabula Sapiens dataset (Jones et al., 2022). Log10-transformed nCPM values per gene are indicated per gene, and a row-wise scaling was applied for color mapping across the cell types. The lower panel indicates gene expression of cluster markers for arterial (*GJA5*), venous (*ACKR1*), capillary (*CA4*), and lymphatic (*LYVE1*) endothelial cells.

Members of the BAR protein family exhibit specificity for distinct membrane curvatures, making the highly folded membrane topology at AAJs ideally suited for their selective recruitment. The F-BAR protein PACSIN2 decorates the follower cell side of AAJs (Fig. 2 a) (Dorland et al., 2016). However, the FIB-SEM dataset showed that AAJs contained concave and convex membrane geometries extending beyond PACSIN2-positive regions. We therefore hypothesized that additional BAR proteins are recruited to the AAJs. To test this hypothesis, we screened a library of 41 expression plasmids encoding GFP-tagged BAR proteins, which were transfected at least three independent times in human umbilical vein endothelial cells (HUVECs), and assessed their localization (Table S1 and Fig. S2). In addition to PACSIN2, we found that at least 18 out of the 41 tested BAR proteins were recruited to AAJs (Fig. 2 b, Fig. S2, and Table S1); being tyrosine-protein kinase Fer (FER), F-BAR domain only protein 1 (FCHO1), Cdc42-interacting protein 4 (CIP4), formin-binding protein 17 (FBP17/FNBP1), PRKCA-binding protein (PICK1), SNX9, bridging integrator 1–3 (BIN1, BIN2, BIN3), endophilin-A2 (SH3GL1), endophilin-A1 (SH3GL2), endophilin-A3 (SH3GL3), SH3 domain-binding protein 1 (ARHGAP43), Amphiphysin 1 (AMPH), Rho GTPase–activating protein 17 (ARHGAP17/RICH1), Brain-specific angiogenesis inhibitor 1-associated protein 2-like protein 2 (Pinkbar/BAIAP2L2), ArfGAP with coiled-coil, ankyrin repeat and PH domains 3 (ACAP3/Centaurin-b5), and PH domain–containing protein 1 (ASAP1/DEF-1). Of note, these BAR proteins exhibited selective differences in junctional recruitment, both in the extent of their junctional distribution (e.g., see FER versus BIN2) and in the fraction of AAJs with which they associated (e.g., see BIN1 versus SNX9) (Fig. 2 b). We assessed the endogenous expression levels of these junctional BAR proteins in human ECs within publicly available transcriptomic datasets, which showed that nine junctional BAR proteins were expressed in human ECs (ACAP3, ARHGAP17, ASAP1, BIN1, BIN3, CIP4 (*TRIP10*), endophilin-A2 (*SH3GL1*), FBP17 (*FNBP1*), FER, PICK1, and SNX9 (Fig. S3 a). Analysis of gene expression levels for BAR proteins in human vascular scRNA-seq data (Jones et al., 2022) further shows that *ASAP1*, *TRIP10*, *SH3GL1*, *FNBP1, FER*, *PICK1*, and *SNX9* are expressed at similar levels across arterial, venous, capillary, and lymphatic EC types (Fig. S3 b). *BIN1* is enriched in lymphatic endothelium compared with other vascular clusters, whereas *ARHGAP17* and *BIN3* are more constrained to non-lymphatic ECs (Fig. S3 b). Next, we investigated the bona fide localization of endogenous BAR proteins in ECs using a selection of commercially available validated antibodies for immunofluorescence, which confirmed the presence of ARHGAP17, ASAP1, BIN1, endophilin-A2, and SNX9 at AAJs (Fig. 2 c). These results clearly demonstrate that multiple BAR proteins are recruited to endothelial AAJs.

### BIN1 and SNX9 control endothelial front-rear polarity and collective cell migration

**Figure S4.**
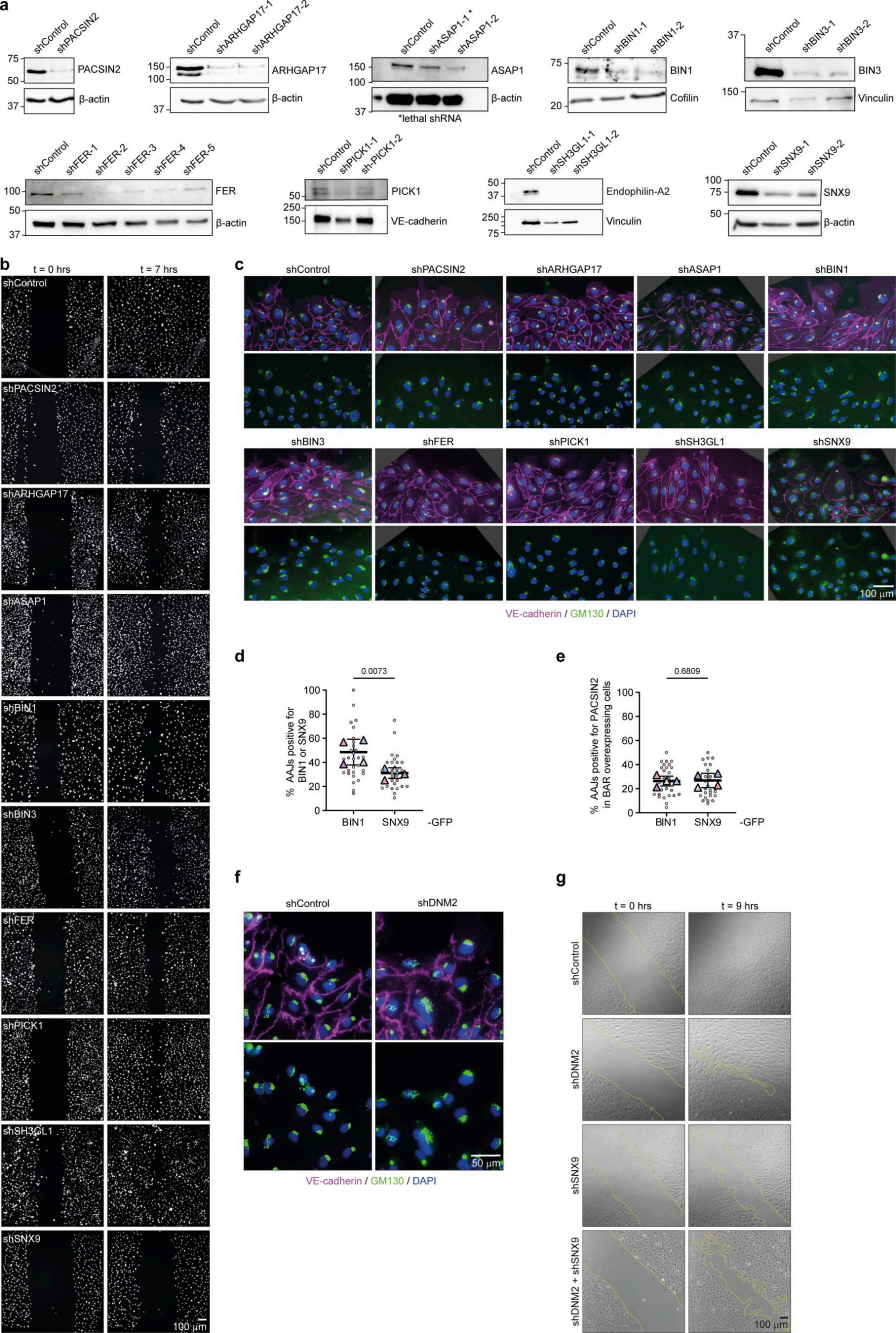
**Validation of shRNAs for BAR proteins, BAR-GFP overexpression, and DNM2 knockdowns. (a)** Representative western blots showing shRNA knockdown efficiency for the indicated BAR proteins with targeting shRNAs. Asterisk indicates an shRNA whose knockdown is lethal to cells. **(b)** Representative widefield images of wound migration at t = 0 and t = 7 h after scratch in HUVECs depleted with shRNAs of indicated BAR proteins stained with Hoechst. See also Video 4. **(c)** Representative images of collectively migrating HUVECs transduced with shRNAs targeting the indicated BAR proteins, fixed 6 h after scratch wound formation. Nuclei are stained with DAPI (blue), junctions with VE-cadherin (magenta), and Golgi with GM130 (green). **(d and e)** Dot plots showing the percentage of AAJs per cell that are positive for BIN1-GFP or SNX9-GFP (d) and dot plots showing the percentage of AAJs per cell that are positive for PACSIN2 in cells expressing BIN-GFP or GFP-SNX9 (e). Data are presented as mean ± SD. Circles indicate individual data points and triangles indicate means of individual experiments with colors corresponding to the experimental replicates. Statistical analysis was performed using a two-sided Student’s *t* test. *n =* 29 cells for BIN1-GFP and 28 for GFP-SNX9 from four independent experiments. P values are indicated on the graph. **(f)** Representative images of migrating HUVECs transduced with shControl and shDNM2-1, fixed 6 h after scratch wound formation. Nuclei are stained with DAPI (blue), junctions with VE-cadherin (magenta), and Golgi with GM130 (green). **(g)** Representative phase contrast images of wound migration at t = 0 and t = 9 h after scratch in HUVECs transduced with shControl and shDNM2-1. See also Video 13. Scale bars = 100 and 50 μm. Source data are available for this figure: SourceData FS4.

**Figure 3.**
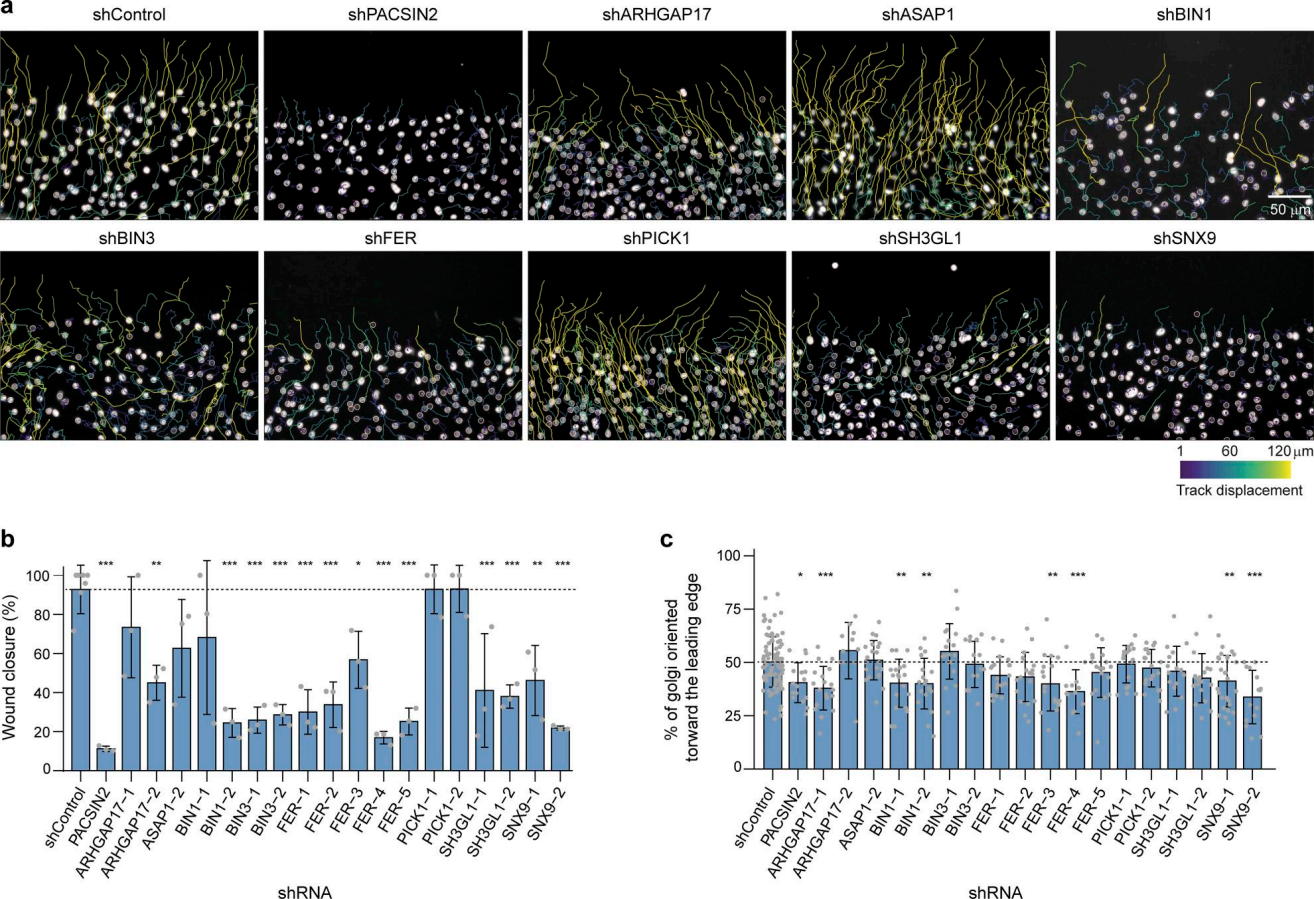
**BIN1, FER, and SNX9 control endothelial collective cell migration. (a)** Representative widefield images of migrating HUVEC monolayers transduced with shRNAs targeting the indicated BAR proteins, captured 2 h after scratch wound formation. Nuclei are stained with Hoechst (greyscale). Migration tracks, shown for the 2–9 h period after scratch, illustrate differences in cell migration dynamics between conditions. Scale bar = 50 μm. **(b)** Quantification of endothelial sheet wound closure 9 h after scratch. For each shRNA, at least *n* = 3 movies were quantified from at least three independent experiments. **(c)** Quantification of Golgi orientation in the first four rows of follower cells at the scratch edge. For each shRNA, at least *n* = 5 images per experiment were quantified from at least three independent experiments. Data are presented as mean ± SD, and the statistical analysis was performed using one-way ANOVA, followed by Dunnett’s test for multiple comparisons versus shControl. * = P < 0.05, ** = P < 0.01, and *** = P < 0.001.

Endothelial collective migration and front-rear polarity depend on the proper turnover of AAJs (Malinova et al., 2021). To investigate whether endothelial-expressed junctional BAR proteins regulate collective cell migration, we performed scratch wound assays of HUVEC monolayers in which the expression of individual BAR proteins was depleted by shRNAs. Cells were seeded at the same densities, and at the time of scratch wounding, cells were ∼100% confluent. Notably, in shBIN1 conditions, cultures were slightly less confluent, despite plating a similar number of cells, suggesting that BIN1 depletion may affect proliferation. Knockdown efficiency was confirmed for ARHGAP17, ASAP1, BIN1, BIN3, FER, PICK1, endophilin-A2 (*SH3GL1*), and SNX9 by western blot (Fig. S4 a). We next evaluated two key aspects of cell migration: the efficiency of wound closure by endothelial monolayers and the front-rear polarity of follower cells, which reflects the level of coordination within the migrating collective (Carvalho et al., 2019). The ability of ECs to close the wound was significantly affected by the depletion of ARHGAP17, BIN1, BIN3, FER, endophilin-A2, SNX9, and PACSIN2 (positive control) (Fig. 3, a and b; Fig. S4 b; and Video 4). Among those junctional BAR proteins, the knockdown of BIN1, FER, and SNX9 also resulted in decreased front-rear polarity for at least two independent shRNAs (Fig. 3 c and Fig. S4 c). While the depletion of BIN3 and endophilin-A2 decreased wound closure capacity, there was no significant difference in their front-rear polarity (Fig. 3, b and c). For ARHGAP17 knockdowns, different results were obtained depending on the shRNA clones, which prevented a clear assessment of its role in endothelial cell migration. Together, these findings demonstrate that the junctional BAR proteins BIN1, FER, and SNX9 are important for coordinated endothelial cell migration.

**Video 4.** **Effect of BAR protein knockdowns on collective EC migration.** Representative widefield movies for HUVEC monolayers depleted with shRNAs of indicated BAR proteins. Nuclei were labeled with Hoechst and cell migration was imaged for 17 h.

### Spatiotemporal recruitment of PACSIN2, BIN1, and SNX9 correlates with specific stages of junction remodeling

**Figure 4.**
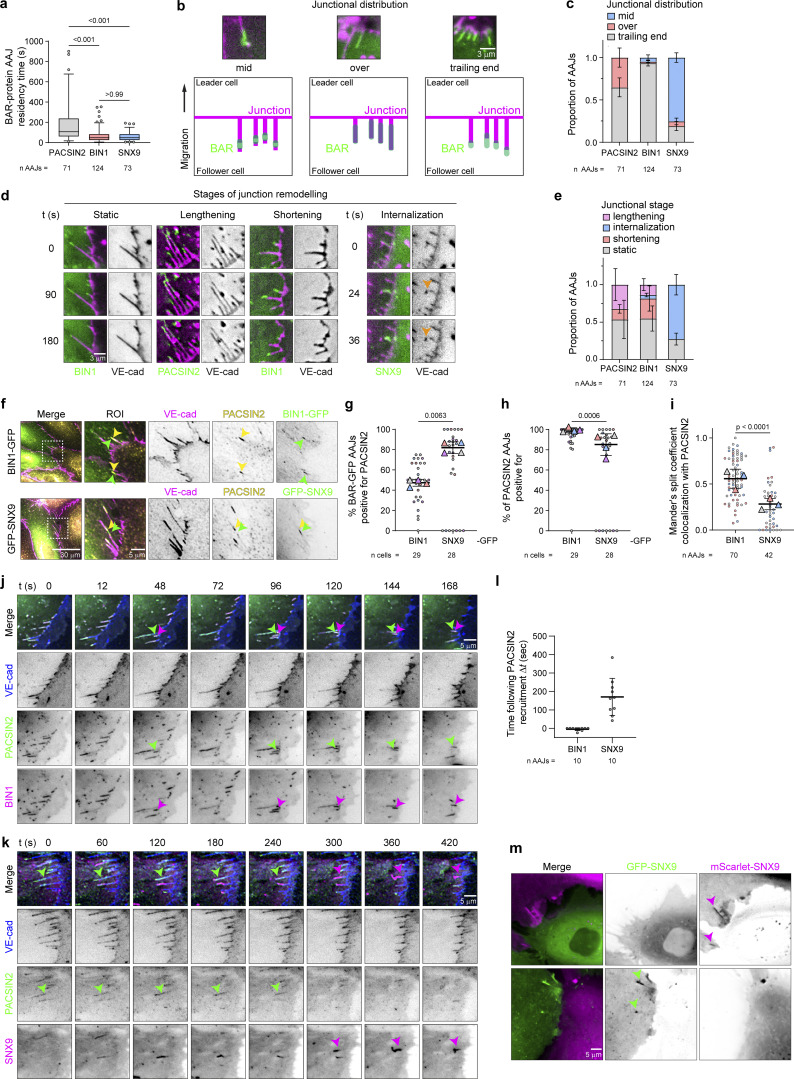
**Spatiotemporal recruitment of PACSIN2, BIN1, and SNX9 correlates with specific stages of junction remodeling. (a)** Quantification of residency time of overexpressed GFP-tagged PACSIN2, BIN1, and SNX9 at AAJs in collectively migrating BOECs following scratch wounding, from widefield time-lapse recordings acquired at 6-s intervals. *n* = 71, 124, and 73 AAJs positive for the different proteins, respectively, from three independent experiments. **(b)** Widefield fluorescence images of BAR protein distribution along AAJs from collectively migrating BOECs expressing VE-cadherin–mScarlet (magenta) and GFP-tagged BAR proteins (green). Schematic drawings show how subjunctional distribution was classified into three categories based on the localization of BAR proteins relative to VE-cadherin: mid, over, or trailing end. **(c)** Quantification of the proportion of AAJs in which the indicated GFP-tagged BAR proteins localize to each of the defined subjunctional regions. *n* = 71, 124, and 73 AAJs positive for the different proteins, respectively, from three independent experiments. **(d)** Widefield time-lapse sequence depicting stages of AAJ remodeling from collectively migrating BOECs expressing VE-cadherin–mScarlet (magenta) and GFP-tagged BAR proteins (green). Merged and VE-cadherin channels are shown to highlight the progression of remodeling events associated with the presence of each BAR protein. Orange arrows indicate VE-cadherin internalizing events at AAJ trailing ends. **(e)** Bar graphs showing the distribution of remodeling stages among AAJs positive for the indicated GFP-tagged BAR proteins. *n* = 71, 124, and 73 AAJs positive for the different proteins, respectively, from three independent experiments. **(f)** Widefield images of collectively migrating BOECs expressing BIN1-GFP or GFP-SNX9 (green), immunostained for VE-cadherin (magenta) and PACSIN2 (yellow). White boxes indicate ROIs that are magnified in the right panels, highlighting AAJs positive for both PACSIN2 and the indicated BAR protein. Green arrowheads indicate regions of BIN1 or SNX9 recruitment, and yellow arrowheads indicate regions of PACSIN2 recruitment along AAJs. **(g)** Dot plots showing the percentage of BIN1- or SNX9-positive AAJs per cell that are positive for PACSIN2. *n =* 29 cells for BIN1-GFP and 28 for GFP-SNX9, from three independent experiments. **(h)** Dot plots showing the percentage of PACSIN2-positive AAJs per cell that are positive for BIN1 or SNX9. *n =* 29 cells for BIN1-GFP and 28 for GFP-SNX9, from three independent experiments. **(i)** Dot plots of Mander’s split colocalization coefficient of BIN1-GFP and GFP-SNX9 with PACSIN2 at AAJs. *n* = 70 and 42 junctions, respectively, from three independent experiments. **(j and k)** Widefield time-lapse images of collectively migrating BOECs expressing VE-cadherin–mTurquoise (blue), PACSIN2-GFP (green), and BIN1-mScarlet (magenta) (j) or mScarlet-SNX9 (magenta) (k) imaged at 6-s time resolution (see Videos 7 and 8). Arrowheads indicate signal of junctional BAR proteins (green for PACSIN2, magenta for BIN1 or SNX9). **(l)** Dot plots showing differences in recruitment timing of BIN1 and SNX9 following junctional PACSIN2 recruitment. *n* = 10 AAJs per condition, from at least three independent live-imaging recordings. **(m)** Representative widefield time-lapse images of mosaic AAJs between HUVECs expressing lentiviral GFP-SNX9 (green) or mScarlet-SNX9 (magenta) from three independent experiments. Arrowheads indicate junctional recruitment of SNX9-GFP (green) or SNX9-mScarlet (magenta) (See Video 9). Data are presented as median in a (box plots show interquartile range 25–75 percentiles, and whiskers representing the 5–95 percentiles); as mean ± SEM in (c and e) and mean ± SD of experiment means in g, h, i, and l, circles indicate individual data points and triangles indicate means of individual experiments with colors corresponding to the experimental replicates. Statistical analysis was performed using a Kruskal–Wallis test followed by Dunn’s multiple comparisons test in a, Mann–Whitney test in g and h, and a two-sided Student’s *t* test in i. P values are indicated on the graphs. Scale bars = 30, 5 and 3 μm.

The recruitment of BAR proteins depends on the local curvature and lipid composition of membranes (Chan Wah Hak et al., 2018; Simunovic et al., 2019; Simunovic et al., 2015; Taylor et al., 2011), pointing to potential distinctive functions of individual BAR proteins at AAJs. The role of junctional PACSIN2 recruitment is to limit VE-cadherin internalization (Dorland et al., 2016; Malinova et al., 2021). Since we validated the junctional localization of BIN1 and SNX9 at the endogenous level, we next investigated their role in junction dynamics. Live imaging of collectively migrating BOECs expressing GFP-tagged BIN1, SNX9, or PACSIN2 together with VE-cadherin–mScarlet showed that each BAR protein resided at AAJs for different durations (Video 5). Quantification of the junctional residency time demonstrated that BIN1 and SNX9 (average of 67 and 60 s, respectively) persisted threefold shorter compared with PACSIN2 (180 s) (Fig. 4 a). Furthermore, we observed that the colocalization of each BAR protein with VE-cadherin diverged across the length of the AAJs. To quantify this, we defined three types of junctional distributions of BAR proteins: “mid,” if the BAR protein connected seemingly separate VE-cadherin structures; “over, ” when the BAR protein colocalized with the VE-cadherin–positive area; and the “trailing end,” when the BAR protein extended beyond the VE-cadherin area (Fig. 4 b). This analysis indicated that PACSIN2 and BIN1 were predominantly associated with the trailing end of AAJs (65 and 94%, respectively). By contrast, SNX9 was mostly localized in the mid-junctional region (75%) (Fig. 4 c). These results indicate that distinct subjunctional regions can be discerned along AAJs, which are characterized by spatial recruitment patterns of BIN1, SNX9, and PACSIN2.

**Video 5.** **BAR protein recruitment and AAJ remodeling.** Representative widefield movies of remodeling AAJs in collectively migrating BOECs overexpressing VE-cadherin–mScarlet (magenta) and indicated BAR proteins SNX9, BIN1, or PACSIN2 (green). Time resolution = 6 s per frame.

AAJs can exist as relatively static structures or undergo remodeling as observed by junction lengthening, shortening, and internalization events. To investigate whether the subjunctional presence of BAR proteins is related to junctional remodeling stages, we live-imaged collectively migrating BOECs expressing VE-cadherin–mScarlet and GFP-tagged BAR proteins (Video 6). The presence of PACSIN2 over or at the trailing end of AAJs was associated with lengthening or static AAJs (Fig. 4, d and e), consistent with its junctional protective function (Dorland et al., 2016; Malinova et al., 2021). The recruitment of BIN1 occurred at static and shortening junctions (Fig. 4, d and e). Interestingly, SNX9, the only BAR protein with prominent affinity for mid-junctional localization, was specifically enriched at internalizing junctions (Fig. 4, d and e). Given the strong difference between BIN1, SNX9, and PACSIN2 in their subjunctional distribution and their recruitment correlating with specific junctional remodeling stages, we conclude that individual BAR proteins function in a coordinated spatiotemporal manner during AAJ remodeling.

**Video 6.** **Stages of AAJ remodeling.** Representative widefield movies of remodeling AAJs in collectively migrating BOECs overexpressing VE-cadherin–mScarlet (magenta) and BAR proteins (green). Time resolution = 6 s per frame.

Next, to dissect how individual BAR domain proteins interrelate at AAJs, we immunostained BIN1-GFP– and GFP-SNX9–expressing ECs for endogenous PACSIN2 and VE-cadherin. BIN1-GFP localized to ∼45% of AAJs, while SNX9 localized to ∼30% of AAJs (Fig. S4 d). Overexpression of either BIN1 or SNX9 did not change the recruitment of PACSIN2 to AAJs (Fig. S4 e). Most SNX9-positive AAJs also recruited PACSIN2, while only ∼50% of the BIN1-positive AAJs contained PACSIN2 (Fig. 4, f and g). Vice versa, BIN1 co-occurred with most PACSIN2-positive AAJs, while SNX9 overlapped with a subset (Fig. 4 h). Furthermore, at double BAR protein–positive junctions, BIN1 strongly colocalized with PACSIN2, while SNX9 and PACSIN2 colocalized to a significantly lesser extent, consistent with their distinct subjunctional distribution (Fig. 4 i). Next, we live-imaged mScarlet-tagged BIN1 or SNX9 simultaneously with PACSIN2-GFP and VE-cadherin–mTurquoise to determine their junctional recruitment dynamics. BIN1 consistently colocalized with PACSIN2 at short AAJs, but its signal diminished as junctions elongated, while PACSIN2 remained strongly present. Conversely, transient spikes in BIN1 intensity coincided with junctional shortening through compaction (Fig. 4 j and Video 7). In contrast, SNX9 recruitment followed PACSIN2 with an average delay of 170 ± 101 s (*n* = 10 AAJs) and preceded the emergence of a mid-junctional domain from which VE-cadherin internalized into follower cells, along with a concurrent drop in PACSIN2 signal (Fig. 4, k and l; and Video 8). To determine from which cell side SNX9 is recruited, we live imaged mosaic endothelial monolayers, in which half the cell population expressed lentivirally transduced GFP-SNX9 and the other half mScarlet-SNX9. SNX9 signal at AAJs was either GFP or mScarlet (Fig. 4 m and Video 9), indicating unilateral recruitment from the follower cell side to AAJs. Altogether, these findings show that different stages of AAJ remodeling are associated with specific spatiotemporal recruitment patterns of PACSIN2, BIN1, and SNX9.

**Video 7.** **BIN1 and PACSIN2 recruitment to AAJs.** Representative widefield movies of remodeling AAJs in collectively migrating BOEC overexpressing VE-cadherin–mTurquoise (blue), BIN1-mScarlet (magenta), and PACSIN2-GFP (green). Arrowheads indicate junctional recruitment of PACSIN2 (green) or BIN1 (magenta). Time resolution = 6 s per frame.

**Video 8.** **SNX9 and PACSIN2 recruitment to AAJs.** Representative widefield movies of remodeling AAJs in collectively migrating BOECs overexpressing VE-cadherin–mTurquoise (blue), mScarlet-SNX9 (magenta), and PACSIN2-GFP (green). Arrowheads indicate junctional recruitment of PACSIN2 (green) or SNX9 (magenta). Time resolution = 6 s per frame.

**Video 9.** **SNX9 at AAJs is derived from one side.** Representative widefield movies of mosaic AAJs between HUVECs overexpressing GFP-SNX9 (green) or mScarlet-SNX9 (magenta). Time resolution = 8 s per frame.

### SNX9 associates with dynamin-2 during asymmetric junction remodeling

**Figure 5.**
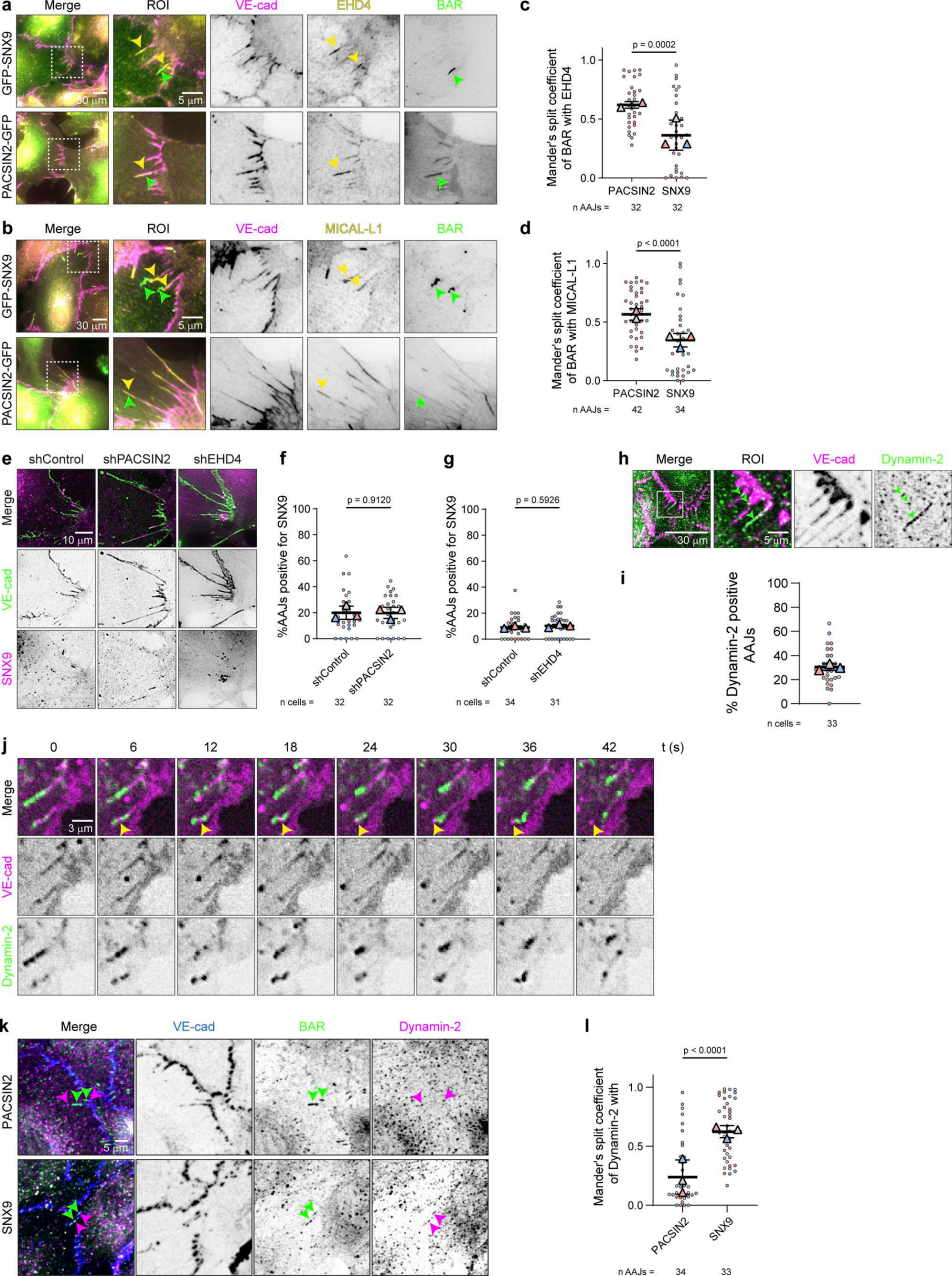
**SNX9 associates with dynamin-2 during asymmetric junction remodeling. (a and b)** Widefield images of GFP-SNX9 and PACSIN2-GFP (green) expressing HUVECs immunostained for VE-cadherin (magenta) and either EHD4 (a) or MICAL-L1 (b) (yellow). The white squares indicate the ROIs that are magnified in the right panels. Green arrowheads indicate AAJs with BAR proteins and yellow arrowheads indicate AAJs with EHD4 (a) or MICAL-L1 (b). **(c and d)** Dot plots of Mander’s split colocalization coefficient of PACSIN2-GFP or GFP-SNX9 with EHD4 (c) and MICAL-L1 (d) at AAJs. *n* = 32 AAJs for PACSIN2/EHD4, 32 for SNX9/EHD4, 42 for PACSIN2/MICAL-L1, and 34 for SNX9/MICAL-L1 from three independent experiments. **(e)** Widefield images of PACSIN2- and EHD4-depleted HUVECs immunostained for SNX9 (magenta) and VE-cadherin (green). **(f and g)** Dot plots indicating the percentage of SNX9-positive AAJs of total AAJs per cell in HUVECs transduced with shControl and shPACSIN2 (f) or shControl and shEHD4 (g). *n* = 32 for shControl, 32 for shPACSIN2 in f and *n* = 34 for shControl, and 31 for shEHD4 in g from three independent experiments. **(h)** Confocal images of HUVECs immunostained for VE-cadherin (magenta) and dynamin-2 (green). Green arrowheads indicate dynamin-2–positive AAJs. **(i)** Quantification of the percentage of dynamin-2–positive AAJs of total AAJs per cell in HUVECs. *n* = 33 cells from three independent experiments. **(j)** Widefield time-lapse sequence depicting dynamin-2–GFP recruitment to internalizing VE-cadherin–mScarlet-positive AAJs (See Video 10). Yellow arrowheads indicate AAJs that undergo internalization events. **(k)** Maximum intensity projection of confocal z-stacks of HUVECs immunostained for VE-cadherin (blue), dynamin-2 (magenta), and either PACSIN2 or SNX9 (green). Magenta arrowheads indicate regions of dynamin-2 recruitment, and green arrowheads indicate regions of PACSIN2/SNX9 recruitment at AAJs. **(l)** Dot plots showing Mander’s split colocalization coefficient between junctional PACSIN2 or SNX9 and dynamin-2. *n* = 34 AAJs for PACSIN2, and 33 for SNX9 from three independent experiments. Data are presented as mean ± SD of experiment means. Statistical analysis was performed using a two-sided Student’s *t* test in c, d, f, and g and a Mann–Whitney U test in l. Circles indicate individual data points, and triangles indicate means of individual experiments with colors corresponding to the experimental replicates. P values are indicated on the graphs. Scale bars = 30, 10, 5, and 3 μm.

The sequential recruitment of SNX9 to PACSIN2-positive AAJs points to a potential interdependence with trafficking regulators within the PACSIN2/EHD4/MICAL-L1 complex (Malinova et al., 2021). Immunofluorescence imaging of ECs expressing GFP-SNX9 showed very limited colocalization between SNX9 and the PACSIN2 interactors EHD4 and MICAL-L1 at AAJs (Fig. 5, a–d). To investigate whether junctional SNX9 recruitment depends on PACSIN2 or EHD4, we assessed SNX9 localization in shControl, shPACSIN2, and shEHD4 HUVEC monolayers by immunostaining. These experiments showed that the proportion of AAJs displaying junctional SNX9 was unaffected by PACSIN2 or EHD4 knockdowns (Fig. 5, e–g). These results show that the junctional recruitment of SNX9 occurs separately from this complex of curvature-sensing proteins.

SNX9 is recruited separately from the PACSIN2 complex, localizes at internalizing junctions, and has been reported to interact with dynamin-2 through its SH3 domain to mediate membrane scission during endocytosis (Schöneberg et al., 2017). To test whether SNX9 associates with dynamin-2 during VE-cadherin internalization, we performed immunostainings for endogenous VE-cadherin and dynamin-2. Dynamin-2 was detected in punctate structures distributed throughout ECs, consistent with its known role in various endocytic processes (Fig. 5 h). Notably, dynamin-2 also localized to ∼30% of AAJs (Fig. 5, h and i). Live-cell imaging of ECs expressing dynamin2-GFP further revealed that dynamin-2 is recruited to AAJs during junctional scission and internalization (Fig. 5 j and Video 10), corresponding to the temporal recruitment dynamics of SNX9 (Fig. 4 k). Immunofluorescence imaging of dynamin-2 showed that it strongly colocalized with SNX9 at AAJs (Fig. 5, k and l). Together, these observations indicate that SNX9 and dynamin-2 are recruited to the mid region of AAJs coinciding with VE-cadherin internalization.

**Video 10.** **Dynamin-2 recruitment to AAJs.** Representative widefield movies of remodeling AAJs in collectively migrating BOECs overexpressing VE-cadherin–mScarlet and dynamin-2–GFP. Yellow arrowhead indicates a junctional internalization event. Time resolution = 6 s per frame.

### SNX9 controls VE-cadherin turnover at asymmetric junctions

**Figure 6.**
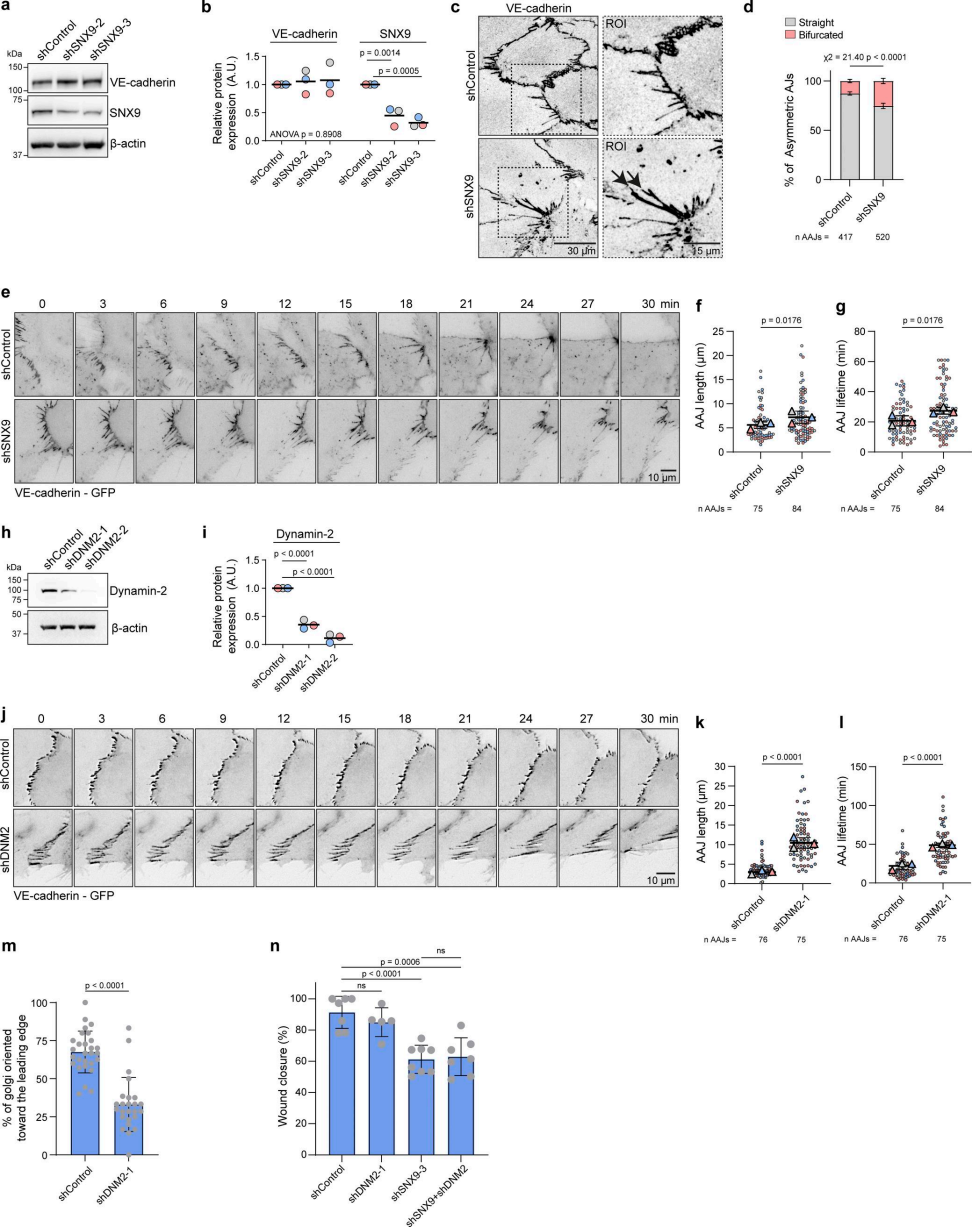
**SNX9 controls VE-cadherin turnover at asymmetric junctions. (a)** Representative Western blot analysis of control and SNX9-depleted HUVECs using indicated shRNAs blotted for VE-cadherin, SNX9, and β-actin. **(b)** Quantification of VE-cadherin and SNX9 protein levels normalized to β-actin, from three independent experiments. **(c)** Confocal images of shControl and shSNX9 HUVECs immunostained for VE-cadherin highlighting AAJs. Black arrows in the ROI highlight representative bifurcated AAJs, where VE-cadherin staining diverges from the main junctional direction. **(d)** Bar graphs displaying the percentage of straight or bifurcated AAJs for shControl and shSNX9 HUVECs. *n* = 417 and 520 AAJs in shControl and shSNX9 ECs, respectively, from three independent experiments. **(e)** Stills from a widefield time-lapse imaging sequence of shControl and shSNX9 BOECs overexpressing VE-cadherin–GFP (See Video 11). **(f)** Dot plots indicating maximum AAJ length in µm during time-lapse imaging of shControl and shSNX9 BOECs. *n* = 75 and 84 AAJs for shControl and shSNX9 ECs, respectively, from three independent experiments. **(g)** Dot plots indicating AAJ lifetime from start to end of AAJ formation and disappearance in minutes during time-lapse imaging of shControl and shSNX9 BOECs. *n* = 75, 84 AAJs for shControl and shSNX9 ECs, respectively, from three independent experiments. **(h)** Representative western blot analysis of control and *DNM2*-depleted HUVECs using indicated shRNAs blotted for dynamin-2 and β-actin. **(i)** Quantification of dynamin-2 protein levels normalized to β-actin, from three independent experiments. **(j)** Stills from a widefield time-lapse imaging sequence of shControl and shDNM2-1 HUVECs overexpressing VE-cadherin–GFP (See Video 12). **(k)** Dot plots indicating maximum AAJ length in µm during time-lapse imaging of shControl and shDNM2-1 HUVECs. *n* = 76, 75 AAJs for shControl and shDNM2-1 ECs respectively, from three independent experiments. **(l)** Dot plots indicating AAJ lifetime from start to end of AAJ formation and disappearance in minutes during time-lapse imaging of shControl and shDNM2-1 HUVECs. *n* = 76, 75 AAJs for shControl and shDNM2 ECs, respectively, from three independent experiments. **(m)** Quantification of Golgi orientation in the first four rows of follower cells at the scratch edge of shControl and shDNM2-1 HUVECs. For each shRNA, at least *n* = 7 images per experiment were quantified from three independent experiments. **(n)** Quantification of endothelial wound closure 9 h after scratch of shControl, shDNM2-1, shSNX9-3, and simultaneous shDNM2 + shSNX9 HUVECs. At least *n* = 2 movies per experiment were quantified from three independent experiments. Data are presented as mean ± SD of experiment means (b, f, g, i, k, l, m, and n) or SEM (d). Circles indicate individual data points, and triangles indicate means of individual experiments with colors corresponding to the experimental replicates. Statistical analysis was performed using one-way ANOVA in b and i, followed by Tukey’s multiple comparisons in *n*, a χ-square test in d, an unpaired two-sided Student’s *t*-test (m), and Mann–Whitney U test in f, g, k, and l. P values are indicated on the graphs. Scale bars = 30, 10, and 5 μm. Source data are available for this figure: SourceData F6.

To investigate whether SNX9 is functionally important for VE-cadherin–based junction turnover, we depleted SNX9 in HUVECs using shRNAs. SNX9 depletion did not affect total VE-cadherin protein levels (Fig. 6, a and b) and shSNX9 ECs formed confluent monolayers with intact VE-cadherin junctions (Fig. 6 c). However, AAJs in shSNX9 ECs appeared less organized, with irregular spacing and bifurcations at their trailing edges compared with the straighter morphology of shControl AAJs (Fig. 6, c and d). Live-cell imaging of VE-cadherin-GFP in shControl and shSNX9 BOECs confirmed these changes: compared with controls, AAJs were on average 27% longer and exhibited a 30% increase in junctional lifetime before internalization in SNX9-depleted cells (Fig. 6, e–g; and Video 11). To determine whether dynamin-2 acts downstream in this process, we depleted dynamin-2 using two independent shRNA clones (Fig. 6, h and i). The more efficient shRNA (shDNM2-2) resulted in loss of EC adhesion, showing its importance, but precluding functional analysis. The moderate depletion achieved by shDNM2-1 was sufficient to perturb junction turnover, resulting in increased AAJ length and prolonged lifetimes (Fig. 6, j–l; and Video 12). Next, we assessed the role of dynamin-2 in endothelial collective migration. Notably, shDNM2-1 ECs failed to orient the Golgi toward the scratch wound (Fig. 6 m; Fig. S4 f); however, these cells still migrated randomly and closed the wounds within a comparable timeframe with shControls (Fig. 6 n; Fig. S4 g; and Video 13). Combined shSNX9 and shDNM2 knockdown did not further exacerbate the migration defect relative to shSNX9 alone (Fig. 6 n; Fig. S4 g; and Video 13). Together, these findings show that SNX9 regulates VE-cadherin turnover at AAJs, an effect that may be partially mediated through the recruitment of dynamin-2.

**Video 11.** **SNX9 controls VE-cadherin turnover at AAJs.** Representative widefield movies of shControl and shSNX9 BOECs overexpressing VE-cadherin–GFP. Scale bar = 20 µm. Time resolution = 1 min per frame.

**Video 12.** **Dynamin-2 controls VE-cadherin turnover at AAJs.** Representative widefield movies of shControl and shDNM2 HUVECs overexpressing VE-cadherin–GFP. Scale bar = 20 µm. Time resolution = 30 s per frame.

**Video 13.** **Effect of SNX9 and DNM2 knockdowns on collective EC migration.** Representative phase contrast movies of scratch assays in HUVEC monolayers transduced with shControl or shRNAs targeting DNM2 and/or SNX9. Cell migration was imaged for 18 h. Scale bar = 200 µm. Time resolution = 5 min per frame.

### SNX9 controls endothelial collective migration within the common cardinal vein of zebrafish

**Figure S5.**
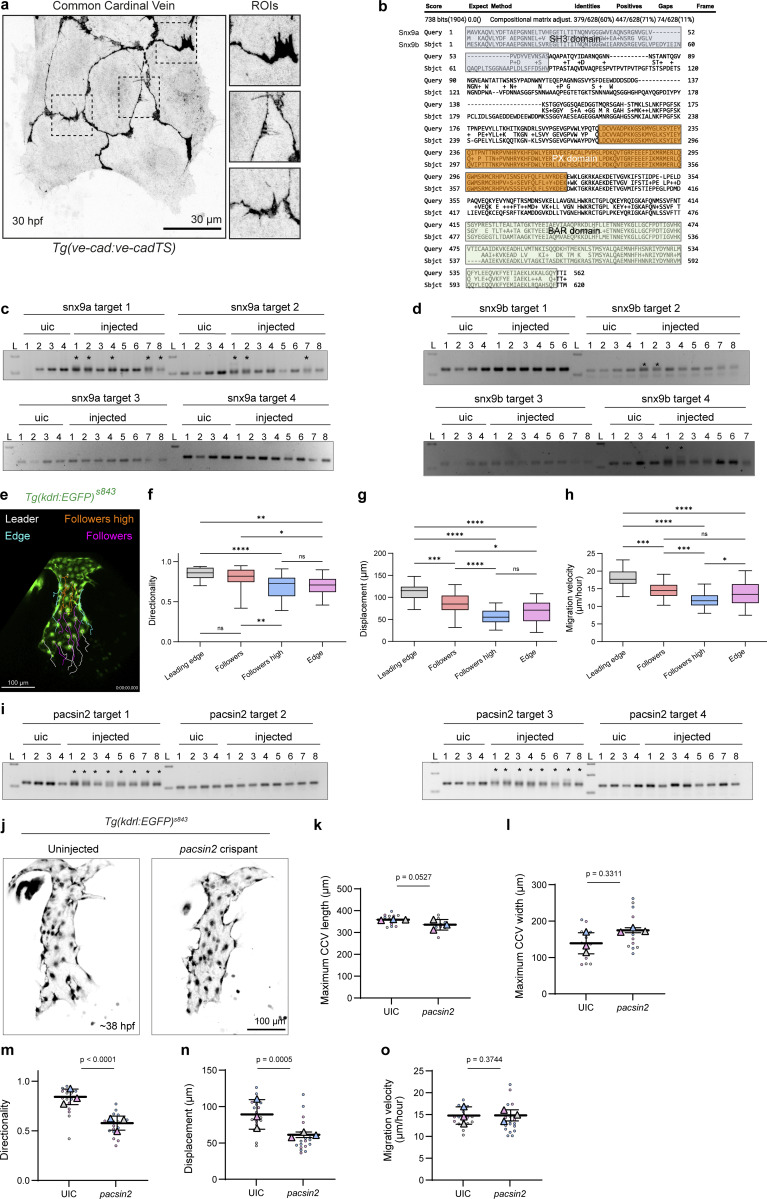
**Snx9 alignment, knockout efficiency of gRNA pools and CCV migration parameters. (a)** Maximum projection of an Airyscan confocal image and ROIs of endothelial junctions in the CCV of a *Tg(ve-cad:ve-cadTS)* zebrafish embryo at 30 hpf. **(b)** Sequence homology alignment of zebrafish proteins Snx9a and Snx9b using Blast. SH3, PX, and BAR domains are indicated to indicate homology of these protein domains. **(c and d)** Validation of targeting efficiency by PCR analysis of each amplicon targeted by individual s*nx9a or snx9b* gRNAs in the gRNA pool. Uninjected siblings from each experiment are compared against injected embryos showing indels causing the appearance of additional amplicons or loss of distinctive bands for lanes indicated with asterisks. **(e)** GFP image of the CCV of an uninjected *Tg(kdrl:EGFP)*^*s843*^;*Tg(fli1a:H2B-mCherry)*^*uq37bh*^ zebrafish embryo. Lines indicate individual cell trajectories starting at 31 and ending at 40 hpf. Trajectories are color-coded based on starting cell location: leader cells (white), follower cells (magenta), cells at the dorsal side of the CCV with a double endothelial cell layer (followers high, orange), and cells at the lateral edges (edge, cyan). **(f–h)** Box-and-whisker plots representing single cell migration parameters separated by starting location in the CCV quantified for directionality (f), displacement (g), and cell velocity (h). *n* = 17, 28, 38, and 21 for leading cells, followers, dorsal followers, and lateral cells, respectively, from 4 to 5 replicate experiments. Data are presented as median with 25–75 percentiles, and whiskers indicate min-max values. Statistical analysis was performed by a one-way ANOVA test followed by Tukey HSD post hoc tests. P <0.05 = *, P < 0.01 = **, P < 0.001 = ***, and P < 0.0001 = ****. **(i)** Validation targeting efficiency by PCR analysis of each amplicon targeted by individual *pacsin2* gRNAs in the gRNA pool. Uninjected siblings from each experiment are compared against injected embryos showing indels causing the appearance of additional amplicons or loss of distinctive bands for lanes indicated with asterisks. **(j)** Maximum projection of the CCV at 38 hpf in uninjected and *pacsin2* crispants. **(k and l)** Dot plots representing quantification of maximum CCV length (k) and width (l) at 40 hpf for uninjected embryos compared with *pacsin2* sibling crispants. *n* = 13, and 11 CCVs, respectively, from three independent experiments; from which one used a previously run control group in Fig. 7. **(m–o)** Dot plots representing single cell migration parameters acquired from tracks of follower cells for an 8-h period: directionality (m), displacement (n), and migration velocity (o) in *pacsin2* crispants compared with their uninjected siblings. *n* = 18, 19 follower cells for *uic* and *pacsin2* crispants, respectively, from three independent experiments; one of which used a previously run control group in Fig. 7. Data are presented as mean ± SD of experiment means. Circles indicate individual data points, and triangles indicate means of individual experiments with colors corresponding to the experimental replicates. Statistical analysis was performed by the Mann–Whitney U test. *P*-values are indicated on the graphs. Scale bars = 100 and 30 μm. Source data are available for this figure: SourceData FS5.

**Figure 7.**
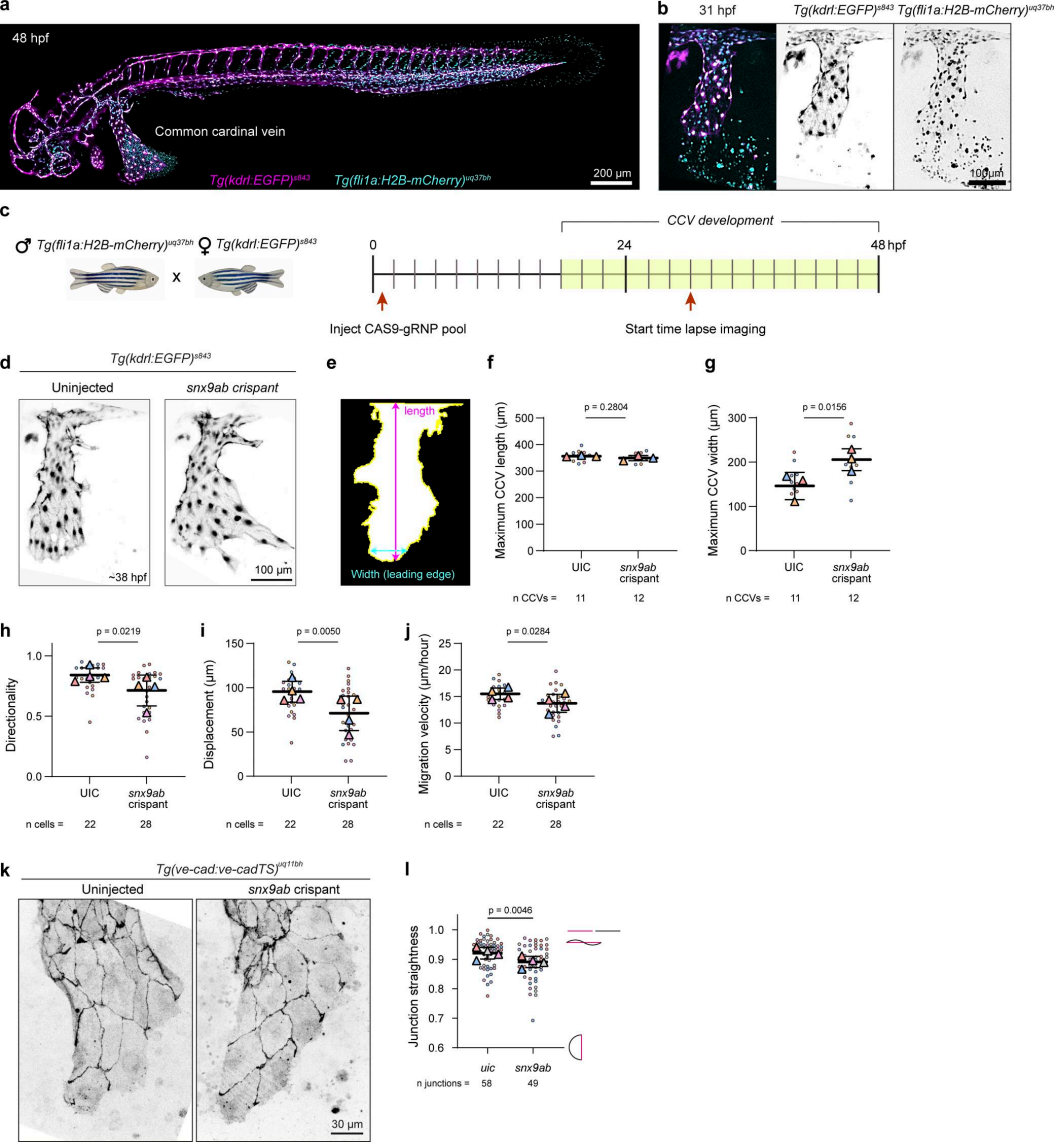
**SNX9 controls endothelial collective migration within the CCV of zebrafish. (a)** Zebrafish 48 hpf embryo with *Tg(kdrl:EGFP)*^*s843*^ blood vasculature in magenta and *Tg(fli1a:H2B-mCherry)*^*uq37bh*^ endothelial cell nuclei in cyan. **(b)** Images from a time-lapse sequence showing EGFP from *Tg(kdrl:EGFP)*^*s843*^ (blood vasculature, magenta) and mCherry (nuclei, cyan) from *Tg(fli1a:H2B-mCherry)*^*uq37bh*^ expression in the CCV at 31 hpf. **(c)** Schematic representation of experimental set-up for CRISPR/CAS9-gRNP injections into the yolk of 1-cell stage embryos from *Tg(kdrl:EGFP)*^*s843*^ and *Tg(fli1a:H2B-mCherry)*^*uq37bh*^ crosses and microscopy pipeline to image cell migration dynamics during the development of the CCV between ∼24–48 hpf. **(d)** Maximum projection of the CCV at 38 hpf in uninjected and *snx9ab* crispants. **(e)** Schematic of quantification set-up with maximum CCV length and width. **(f and g)** Dot plots representing quantification of maximum CCV length (f) and width (g) at 40 hpf for uninjected embryos compared with *snx9* sibling crispants. *n* = 11, and 12 CCVs, respectively, from three independent experiments. **(h–j)** Dot plots representing single-cell migration parameters acquired from tracks of follower cells for an 8-h period: directionality (h), displacement (i), and migration velocity (j) in *snx9ab* crispants compared with their uninjected siblings. *n* = 22, 28 follower cells for *uic* and *snx9ab* crispants, respectively, from 4 independent experiments. **(k)** Representative timeframe of a confocal Airyscan maximum projection time-lapse sequence of the leading edge of the CCV of uninjected or *snx9ab* crispant *TgBAC(ve-cad:ve-cadTS)*^*uq11bh*^ siblings at ∼30 hpf. **(l)** Dot plot of quantification of junctional straightness for uninjected and *snx9ab* crispant siblings. Graph is clipped at 0.6, close to 0.637, which is the straightness of a semicircle, to more accurately visualize the distribution of the data. A semicircle, curved line (straightness = 0.96) and straight line (straightness = 1) are indicated on the graph as reference points. *n =* 58 and 49 junctions for uic and *snx9ab* crispant embryos, respectively, from four independent experiments. Data are presented as mean ± SD of experiment means. Circles indicate individual data points, and triangles indicate means of individual experiments with colors corresponding to the experimental replicates. Statistical analysis was performed by Mann–Whitney U test in f–j and l. P values are indicated on the graphs. Scale bars = 200, 100, and 30 μm.

To assess the importance of SNX9 for *in vivo* endothelial collective behavior, we next studied the development of the common cardinal veins (CCVs) in zebrafish. CCV development occurs through collective endothelial migration, where VE-cadherin–mediated junctions guide directional movement (Hamm et al., 2016; Helker et al., 2013). During this process, junctions are formed and dynamically remodeled in a manner reminiscent of AAJs observed in cultured endothelial cells (Fig. S5 a). The CCVs initially form as open-ended tubes and subsequently connect to the heart via the migration of an endothelial sheet by 50 h postfertilization (hpf). The zebrafish genome contains two SNX9 paralogs, *snx9a* and *snx9b,* which share high protein sequence homology (60% identity) particularly across the SH3, PX, and BAR domains (Fig. S5 b). We applied reverse genetics to generate CRISPR/Cas9-mediated *snx9a/b* double knockout F0 mutants (Wu et al., 2018), hereafter referred to as crispants. *Snx9a/b* crispants were generated by injecting CAS9-gRNPs of four pooled gRNAs per gene into *Tg(kdrl:EGFP)*^*s843*^;*Tg(fli1a:H2B-mCherry)*^*uq37bh*^ double transgenic embryos to visualize ECs and nuclei, respectively (Fig. 7, a–c; and Fig. S5, c and d). At the gross morphological level, *snx9a/b* crispants were undistinguishable from uninjected siblings, and cardiovascular function was not affected. Next, to determine a role in collective EC migration *in vivo*, we explored CCV development. At 38 hpf, the CCV was significantly wider in *snx9a/b* crispants, which was driven by a posterior divergence of CCV ECs (Fig. 7, d–g; and Video 14). To understand the underlying cause of these changes in CCV morphogenesis, we tracked EC migration during CCV development (∼32 to 40 hpf) and focused on the properties of follower cells, which rely on directional collective migration (Fig. S5, e–h). In *snx9a/b* crispants, follower cell directionality, displacement, and velocity were significantly reduced compared with uninjected siblings (Fig. 7, h–j). Of note, we also observed impaired EC migration in the CCV of *pacsin2* crispant embryos (Fig. S5, i–o), in line with its previously described role in endothelial guidance in the mouse retina (Malinova et al., 2021). To investigate whether the defects in endothelial migration in *snx9a/b* crispants could stem from impaired junctional remodeling, we used Airyscan confocal microscopy to live image VE-cadherin protein dynamics by utilizing the *TgBAC(ve-cad:ve-cadTS)*^*uq11bh*^ transgenic line (Lagendijk et al., 2017). Time-lapse recordings revealed that *snx9ab* crispants still formed AAJ-like structures, although the endothelial cell–cell junctions in migrating CCV endothelial cells appeared noticeably less straight compared with those in uninjected siblings, indicating altered junctional dynamics (Fig. 7, k and l; and Video 15). Together, these findings establish SNX9 as a regulator of endothelial junctions during endothelial collective migration in vascular development.

**Video 14.** ***Snx9ab* depletion deregulates EC migration in the CCV.** Representative movie of endothelial cells in the developing CCV in zebrafish embryos injected with gRNA pools against *snx9ab* or an uninjected sibling. Maximum projections from confocal time-lapses of Z-stacks taken at 30-min intervals for 13 h from *Tg(kdrl:EGFP)*^*s843*^*;Tg(fli1a:H2B-mCherry)*^*uq37bh*^ double transgenic embryos. Channel depicted here is *Tg(kdrl:EGFP)*^*s843*^. Scale bar = 100 µm. Time resolution = 30 min per frame.

**Video 15.** ***Snx9ab* depletion alters VE-cadherin remodeling in the CCV.** Representative maximum projection of an Airyscan-processed z-stack time-lapse of VE-cadherin in endothelial cells of the developing CCV in uninjected and *snx9ab* crispant *Tg(ve-cad:ve-cadTS)*^*uq11bh*^ zebrafish embryos. Z-stacks were processed, and maximum projections were generated from confocal time-lapses for 1 h. Scale bar = 30 µm. Time resolution = 1 min per frame.

## Discussion

Our findings reveal that AAJs are not mere extensions of classical junctions, but represent a plasma membrane curvature-rich junctional state that serves as a platform for the spatiotemporal recruitment of BAR proteins. Our systematic screen identified a surprisingly broad spectrum of BAR proteins localizing to AAJs. From those, BIN1 and SNX9 emerged as key regulators of collective endothelial migration, localizing to junctional subregions where they mediate cell–cell junction remodeling. Importantly, depletion of SNX9 disrupted endothelial junction dynamics and impaired collective migration in zebrafish, highlighting the physiological relevance of curved junctional membranes and their remodeling through multiple BAR proteins for vascular development.

The high-resolution FIB-SEM dataset presented in this study provides the first 3D reconstruction of AAJs, resolving their ultrastructure as double-membrane tubes formed by the apposed plasma membranes of leader and follower cells, where an invagination from the leader cell is tightly wrapped by the follower cell. This “engulfment” architecture, previously hinted at in EM studies (Hayer et al., 2016), is now captured in high detail, providing a precise 3D structural view of how leader and follower membranes interlock to form AAJs. In addition, we generated subjunctional curvature maps of AAJs, showing that they are not uniform tubes, but display pronounced nanoscale heterogeneity along their length. Such curvature diversity provides a structural basis for the recruitment of BAR proteins with different curvature preferences along subjunctional regions. While this analysis allowed us to quantify curvature distributions at nanometer resolution, the lateral and axial resolution of SIM was insufficient to correlate subjunctional BAR-protein localization intensities with specific curvature values from the FIB-SEM data. This limitation warrants further technical developments combining other super-resolution microscopy methods with volumetric EM.

BAR proteins are important for multiple modes of endocytosis; amongst others, they are recruited during clathrin- and endophilin-mediated endocytosis, where their sequential, curvature-dependent recruitment is well characterized: F-BARs bind shallow membrane invaginations, BAR and N-BAR proteins act at more highly curved intermediates, and PX-BAR proteins act at the narrowest necks (Kaksonen and Roux, 2018; Casamento and Boucrot, 2020). Whether an analogous curvature-sorted principle underlies the junctional BAR-protein recruitment and internalization of VE-cadherin at AAJs remains to be determined. Yet, the complex membrane geometry of AAJs, spanning curvature radii from ∼12 to 500 nm, provides a structure that could accommodate distinct BAR proteins according to their intrinsic curvature preferences. Consistent with this, we found that PACSIN2, which associates with membrane curvatures of ∼14–100 nm radius (Wang et al., 2009), localized along large areas of the junctions, and was released from junctional areas prior to the recruitment of SNX9 together with dynamin-2 to mid-junctional regions. SNX9 and dynamin-2 preferentially bind to membranes with narrower curvatures, with radii of ∼20 and 10 nm, respectively (Kaksonen and Roux, 2018; Yarar et al., 2007). Dynamin-2 colocalized with SNX9 but not with PACSIN2, mirroring the inverse PACSIN2–dynamin-2 relationship described at caveolae (Senju et al., 2015). The spatiotemporal recruitment of SNX9 and dynamin-2 in combination with the ring-shape membrane structures observed in AAJ2 of the FIB-SEM data suggest that these junctional regions are characterized by highly narrow membrane curvatures, potentially preparing for internalization. Collectively, these findings support a curvature-sorted hand-off mechanism, in which membrane scission is executed by SNX9-dynamin-2 at tighter junctional curvatures, analogous to the sequential coordination at clathrin-coated pits (Taylor et al., 2011).

However, curvature preference alone is insufficient to explain how the various BAR proteins are recruited to AAJs. The localization screen revealed that not all members of a given BAR subfamily localize to junctions equivalently. This indicates that other protein–protein interactions through other domains and additional cellular determinants beyond membrane geometry govern their recruitment. Resolving which curvature subdomains each BAR protein occupies within AAJs, and what auxiliary factors license their recruitment, represent an intriguing direction for future studies.

A few BAR proteins have previously been described to support endothelial migration during developmental angiogenesis and the regeneration of injured blood vessels (Genet et al., 2019; Malinova et al., 2021; Yuge et al., 2022). We here extended the repertoire of junction-associated BAR proteins with BIN1 and SNX9 as prominent regulators of collective endothelial migration. However, not all junctional-localizing BAR proteins contributed equally to migration: for instance, the knockdown of ASAP1 and PICK1 produced no detectable migration defect, while the depletion of endophilin-A2 and BIN3 delayed wound closure, without affecting follower’s cell polarity. Because several BAR proteins localize to similar regions at endothelial junctions, the loss of one protein may be compensated by others or by increased expression of related family members. As a result, the effects of individual knockdowns may underestimate the overall role of BAR proteins in junctional remodeling and endothelial migration. Future studies using combined genetic perturbations or acute protein degradation approaches will therefore be informative to define their collective functions.

The temporal dynamics of BAR protein recruitment reveal functions that extend beyond endocytosis at remodeling junctions. For instance, PACSIN2 persisted at AAJs during elongation and stable phases, while BIN1 transiently accumulated at short or retracting junctions. This is in line with BIN1’s described capacity to promote actin filament bundling and stabilization (Dräger et al., 2017) and its ability to inhibit dynamin-2 GTPase activity to stabilize membrane invaginations (Cowling et al., 2017; Fujise et al., 2022; Fujise et al., 2021). BIN1 also recruits dynein onto endocytic carriers (Ferreira et al., 2021), raising the possibility that AAJ remodeling is coupled to microtubule-based trafficking. The precise contributions of BIN1 and of the many other junctional BAR proteins that were identified to AAJ dynamics remain an exciting avenue for future research. In this regard, the notion that BIN1 and other BAR proteins, such as SRGAP3, are highly expressed in lymphatic ECs (Fig. S3 b), indicates that differential BAR protein expression may finely tune junctional dynamics across different vascular beds.

VE-cadherin endocytosis is a well-established process that regulates angiogenesis and vascular patterning (Arima et al., 2011; Bentley et al., 2014; Bentley et al., 2009; Grimsley-Myers et al., 2020; Malinova et al., 2021). Our findings add a new layer to this regulation by showing that recruitment of BAR proteins locally controls the balance between junction stabilization and turnover. PACSIN2 and BIN1 act as stabilizers with different dynamics, while SNX9 promotes local VE-cadherin internalization. This interplay allows junctions to maintain sufficient adhesive strength for leader-follower coupling while retaining the plasticity that is needed for remodeling during migration. At the same time, our findings also indicate that there are differences in junction dynamics when comparing migrating endothelial cells in vitro and in vivo. Future work will need to dissect how the localized control at AAJs cross talks with broader angiogenic signaling pathways for vascular development and to understand whether its dysregulation contributes to vascular malformations or pathological angiogenesis.

## Materials and methods

### Cell culture

Pooled HUVECs from multiple donors (C2519A; Lonza) were cultured in Endothelial Cell Growth Medium 2 culture medium supplemented with the Growth Medium 2 Supplement Pack (PromoCell) on gelatin-coated tissue culture plastics. HEK293T cells (CRL-3216; ATCC) were cultured in Dulbecco’s modified Eagle’s medium (DMEM) with L-glutamine and supplemented with 10% FCS and 1% penicillin-streptomycin. Human cord BOECs (Mahlandt et al., 2023) were cultured in growth factor–supplemented EGM2 supplemented with 16% FCS. BOECs and HUVECs were used interchangeably to validate findings across different types of endothelial cells.

### DNA plasmids, lentiviral transduction, and transfections

Knockdown experiments were performed using pLKO.1 lentiviral vectors from the RNAi Consortium (TRC) library (Root et al., 2006). For validated shRNAs, two sequences were selected, and for nonvalidated shRNAs, five shRNAs were used (Table S2). Lentiviral particles were produced in HEK293T cells, which were transiently transfected with third-generation packaging constructs and lentiviral expression plasmids using Trans-IT LT1 (Mirus). HUVECs transduced with shRNAs were analyzed at least 72 h after transduction. Knockdown efficiency was assayed using standard western blotting. For ASAP1, only one shRNA was used as the other shRNA resulted in cell death. Human BAR-protein-GFP transient expression plasmids were a kind gift from Emmanuel Boucrot (University College London, London, UK) (Chan Wah Hak et al., 2018). SNX9 and BIN1 were transferred to a pmScarlet vector to generate mScarlet-SNX9 and BIN1-mScarlet. GFP-SNX9 and mScarlet-SNX9 were cloned into a lentiviral pLV-CMV-IRES-Puro expression plasmid. The lentiviral expression construct pLV-CMV-PACSIN2-GFP was generated before (Dorland et al., 2016). Rat dynamin2-GFP cDNA was a kind gift from Igor Kovačević (Martin Luther University, Halle-Wittenberg, Germany) and cloned into a pLV-CMV-IRES-Puro expression plasmid. pLV-CMV-VE-cadherin-mScarlet-Puro and pLV-CMV-VE-cadherin-mTurquoise2-IRES-Puro were generated by insertion of human VE-cadherin (NM_001795.5) into a pLV-CMV-mScarlet-IRES-PURO or pLV-CMV-mTurquoise2-IRES-Puro plasmid. HUVECs and BOECs were transfected at 80% confluency with plasmids using Lipofectamine 3000 (Invitrogen) or Lipofectamine LTX (Invitrogen) with DNA concentrations ranging between 500–2,000 ng. For lentiviral overexpression, BOECs and HUVECs were transduced at 60% confluence by overnight incubation with lentiviral particles and subsequent selection with 250 ng/ml puromycin (Sigma-Aldrich; #P8833). The following day, cells were reseeded on coverslips for fixation and immunostaining or seeded on Labtek/Mattek coverglass dishes for live imaging.

### Antibodies for immunofluorescence and western blotting

The following antibodies and dyes were used for immunostaining or immunoblotting: mouse anti-human VE-cadherin-647, CD144 55-7H1 (Cat #561567; BD Biosciences, RRID:AB_10712766, 1:200 for IF), mouse anti-human VE-cadherin (Cat #sc-9989; Santa Cruz Biotechnology, RRID:AB_2077957, 1:100 for IF, 1:1,000 for WB), rabbit anti-human VE-cadherin (D87F2) XP (Cat #2500; Cell Signaling, RRID:AB_10839118, 1:100 for IF, 1:1,000 for WB), mouse anti-human GM130 (Cat #610823; BD Biosciences, clone 130, RRID:AB_398142, 1:100 for IF), rabbit-β-actin (Cat #4967; Cell Signaling, RRID:AB_330288, 1:1,000 for WB), mouse anti-human-ASAP1 (Cat #sc-374410; Santa Cruz Biotechnology, RRID:AB_10989256, 1:100 for IF, 1:1,000 for WB), rabbit anti-human-ARHGAP17 (gift from Rafael Garcia-Mata (University of Toledo, Toledo, Ohio, USA) [Kreider-Letterman et al., 2023], 1:100 for IF and 1:3,000 for WB), mouse anti-human ARHGAP17 (Cat #sc-514438; Santa Cruz Biotechnology, 1:100 for IF, 1:500 for WB), mouse anti-human-BIN1 (Cat #sc-23918; Santa Cruz Biotechnology, RRID:AB_667901, 1:200 for WB), rabbit anti-human-BIN1 (Cat# GTX103259; GeneTex, RRID:AB_11165763, 1:100 for IF), mouse anti-human-BIN3 (Cat #sc-514396; Santa Cruz Biotechnology, 1:1,000 for WB), mouse anti-human-Endophilin-A2 (Cat #sc-365704; Santa Cruz Biotechnology, RRID:AB_1085938, 1:100 for IF, 1:1,000 for WB), FER (Cat #sc-390484; Santa Cruz Biotechnology, 1:1,000 for WB), mouse anti-human-PICK1 (Cat #sc-390479; Santa Cruz Biotechnology, 1:1,000 for WB), rabbit anti-human-SNX9 (Cat# 15721-1-AP; Proteintech, RRID:AB_2286415, 1:200 for IF, 1:1,000 for WB), rabbit anti-human-EHD4 (gift from Markus Plomann, University of Cologne, Cologne, Germany), rabbit anti-human-MICAL-L1 (NBP2-55389; Novus Biologicals, RRID:AB_3340623, 1:100 for IF and WB), mouse anti-human dynamin2 (Cat #sc-166669; Santa Cruz Biotechnology, RRID:AB_2093684, 1:100 for IF, 1:1,000 for WB), rabbit anti-human dynamin2 (Cat #NBP2-47477; Novus Biologicals, RRID:AB_2890914, 1:100 for IF, 1:1,000 for WB), rabbit anti-human-PACSIN2 (Cat #AP8088b, RRID:AB_2161856, 1:100 for IF, 1:5,000 for WB), MitoTracker Deep Red (Cat #M22426; Invitrogen, 1:2,000), and DAPI (Cat #62248; Thermo Fisher Scientific, 1:10,000 for IF). Secondary antibodies for IF were anti-mouse and anti-rabbit AlexaFluor-488, AlexaFluor-594, AlexaFluor-647 (Cat# A-21200; Thermo Fisher Scientific, RRID:AB_2535786, A-21201; RRID:AB_2535787, A-21463; RRID:AB_2535869, A-21441; RRID:AB_2535859, A-21442; RRID:AB_2535860, A-21443; RRID:AB_2535861, 1:1,000 for IF). For western blotting, secondary anti-mouse and rabbit antibodies coupled to HRP were obtained from Bio-Rad (Cat #1706515, #1721011, 1:1,000 for WB). Western blots were imaged using enhanced chemiluminescence detection (#34580; Thermo Fisher Scientific) on an Amersham ImageQuant 800 GxP machine (29653452; Cytiva). Protein band intensity was quantified using the FIJI/ImageJ Gel Analyzer plugin.

### FIB-SEM and Cryo-CLEM

Following the workflow reported by Bharathan et al. (2023); Hoffman et al. (2020), cells were cultured on optically flat sapphire disks (3 mm diameter, 50–80 μm thick, Nanjing Co-Energy Optical Crystal Co., Ltd.). Samples were incubated with MitoTracker for 2 min at 37°C, and dipped three times in freezing media containing Fluorobrite media (Thermo Fisher, A1896702), 25% Dextran (31389-100G; Sigma-Aldrich, Mr ∼40,000), and 0.8-pM TetraSpeck microspheres (0.2 μm diameter, T7280; Invitrogen), and subjected to High Pressure Freezing (HPF Compact 01, Wohlwend GmbH) according to manufacturer’s instructions. Residual ice on the non-cell–bearing side of the sapphire disks was removed via scalpel scraping under liquid N_2_. Samples were then loaded into a custom-built cryostat with an imaging window that maintained a sample temperature of ∼77 K for the duration of imaging. Cells were excited via 488 nm (4 W, Coherent, Genesis CX STM), 561 nm (5 W, MPB Communications, 2RU-VFLP-5000-560-B1R), and 642 nm laser (2 W, MPB Communications, 2RU-VFL-P-2000-642-B1R) illumination and imaged via a 100×, 0.85 NA objective lens (Nikon, CFI L Plan EPI CRB) onto an sCMOS camera detector (Orca Flash 4.0 v3, Hamamatsu Corp.). Candidate regions of interest (ROIs) were imaged using 3D-SIM, with a typical field of view of 130 × 130 um (xy) and 8 um (z-depth). Images were processed using the SIM reconstruction algorithm reported by Gustafsson et al. (2008) with the following typical reconstruction parameters: 0.007 Wiener Filter, 0.7 γ apodization, and 15-pixel radii of the singularity suppression at the OTF origins. Chromatic shifts between each color channel were digitally corrected using the TetraSpek beads as alignment fiducials.

After cryo-fluorescence imaging, samples were subjected to the freeze substitution and resin embedding protocol set forth in Hoffman et al. (2020), including staining with 2% OsO4, 0.1% uranyl acetate, and 3% water in acetone under liquid nitrogen. Samples were embedded in Eponate 12 resin (Ted Pella, Inc.). Sapphire disks were removed from the resin block, and the remaining embedded cells were imaged using a micro X-ray CT system (XRadia 510, Carl Zeiss X-ray Microscopy, Inc.) to identify the ROIs identified via SIM imaging. Fluorescence images were overlaid onto X-ray images to ensure proper ROI location. An ultramicrotome (EM UC7, Leica Microsystems) was used to iteratively remove the resin surrounding the region to be imaged in combination with supplemental X-ray CT imaging. Finally, samples were sputter-coated with 10 nm gold and 100 nm carbon (PECS 682; Gatan) to maintain sample conductivity for EM.

Samples were loaded into a custom FIB-SEM described by Xu et al. consisting of a Zeiss Gemini 450 Field Emission SEM and a Zeiss Capella FIB column oriented at 90° to the SEM beam (Xu et al., 2017). Consecutive milling was performed using a 15 nA gallium ion beam source at 30 kV. Images were acquired at 4 × 4 × 4 nm pixel resolution using a 200 kHz readout rate, 0.25 nA electron dose, and 0.9 kV landing energy. FIB-SEM image slices were laterally registered with respect to each other using a Python implementation of the SIFT-alignment algorithm (Lowe, 2004) available at https://github.com/gleb-shtengel/FIB-SEM.

#### Membrane and organelle segmentation

A random forest classifier model was trained from the processed FIB-SEM data using Ilastik (www.ilastik.org) to produce segmentations of endothelial cell plasma membrane and mitochondria, respectively. CLEM image registration between 3D-SIM images and EM segmentations was first performed using mitochondria as fiducial structures via Big Warp in FIJI (https://imagej.net/plugins/bigwarp). Registration refinement was then performed using 3D-SIM images of PACSIN2 and VE-cadherin with EM segmentations of the endothelial plasma membranes.

#### Plasma membrane curvature calculations

Image regions containing endothelial asymmetric junctions were then analyzed to compute local plasma membrane curvature using custom MATLAB code adapted from Claxton (2026)https://www.mathworks.com/matlabcentral/fileexchange/11168-surface-curvature. Briefly, plasma membrane probability maps generated from ilastik were thresholded, and an isosurface was computed for each junction. The average Gaussian curvature at each point along the computed surface was calculated across an ∼60 nm search radius to create curvature maps for each asymmetric junction.

### Transcriptomic data genes encoding BAR-proteins in endothelial cells

Data were obtained using the Human Proteome Atlas RNA single-cell type database https://www.proteinatlas.org/, which summarized expression levels per gene in 81 cell types from 31 datasets. We plotted expression data (number of normalized transcripts per million) of genes encoding human BAR proteins filtered with the following ENSEMBL ID’s:

ENSEMBL_ID;ENSG00000164144;ENSG00000132254;ENSG00000003147;ENSG00000163596;ENSG00000100151;ENSG00000107554;ENSG00000183111;ENSG00000136717;ENSG00000110934;ENSG00000147439;ENSG00000141985;ENSG00000107295;ENSG00000140600;ENSG00000097033;ENSG00000148341;ENSG00000140750;ENSG00000006740;ENSG00000100092;ENSG00000079482;ENSG00000145819;ENSG00000071205;ENSG00000157500;ENSG00000136044;ENSG00000072818;ENSG00000114331;ENSG00000131584;ENSG00000153317;ENSG00000151693;ENSG00000088280;ENSG00000028528;ENSG00000205302;ENSG00000114520;ENSG00000089006;ENSG00000129515;ENSG00000162627;ENSG00000106266;ENSG00000130340;ENSG00000178996;ENSG00000148158;ENSG00000172803;ENSG00000173548;ENSG00000187239;ENSG00000125733;ENSG00000137942;ENSG00000124507;ENSG00000100266;ENSG00000165912;ENSG00000140368;ENSG00000152229;ENSG00000163072;ENSG00000007237;ENSG00000197948;ENSG00000137478;ENSG00000130475;ENSG00000157107;ENSG00000151422;ENSG00000182511;ENSG00000196935;ENSG00000266028;ENSG00000196220;ENSG00000089820;ENSG00000175866;ENSG00000006453;ENSG00000128298;ENSG00000170873;ENSG00000173166;ENSG00000111640;ENSG00000153789;ENSG00000078053;ENSG00000180448;ENSG00000132613; GAPDH was used as reference.

For analysis of gene expression in distinct endothelial cell types, human vascular scRNA-seq data from the publicly available Tabula Sapiens dataset was used (Jones et al., 2022). For each gene, normalized counts per million (nCPM) were extracted for arterial, venous, capillary, and lymphatic endothelial cells. *FAM92B*, *HMHA1*, and *MTSS1L* were not detected. To visualize differential gene expression across endothelial cell clusters, nCPM values were log10-transformed (log10[nCPM + 1]), and genes were hierarchically clustered using Euclidean distance and complete linkage. To highlight relative differences in expression for each gene across clusters, a row-wise scaling was applied to the log-transformed nCPM values for color mapping, while the absolute log-transformed nCPM values were overlaid in each cell. The visualization was done in RStudio using packages ComplexHeatmap, tidyverse, and grid.

### Immunofluorescence stainings

IF stainings of cells were performed by fixing standard cultured or collectively migrating (2–6 h post-scratch wounding) HUVECs/BOECs in 4% PFA in PBS supplemented with 0.9 mM CaCl_2_ and 0.5 mM MgCl_2_ (PBS++) for 5 min at 37°C or 10 min at room temperature. Cells were washed two times in PBS and permeabilized for 5 min at room temperature with 0.5% Triton X-100 in PBS and blocked with 2% bovine serum albumin (BSA) in PBS. Primary and secondary antibodies were diluted in PBS and incubated in the dark for 1 h each. After each incubation, the coverslips were thoroughly washed in PBS. After the last wash, the coverslips were mounted on microscope slides in Mowiol4-88 (#475904; Calbiochem) and DABCO (D27802; Sigma-Aldrich) solution or in ProLong Glass Antifade Mountant (P36980; Invitrogen). For widefield imaging of fixed samples, a NIKON eclipse TI equipped with a CFI Plan 20 × 0.75 NA dry objective or 60× 1.49 NA Apo TIRF oil objective, a Lumencor SOLA SEII light source and standard DAPI, CFP, GFP, mCherry and Cy5 filter cubes were used at room temperature. For live imaging, an Okolab cage incubator and humidified CO_2_ gas chamber set to 37°C and 5% CO_2_ were used during imaging. For confocal imaging of fixed samples, a Leica Stellaris 8 equipped with a 63 × 1.40 NA oil objective, White Light Laser and Power HyD detectors were used at room temperature.

### Scratch wound assays

HUVECs were cultured on standard plastic multiwell plates, glass coverslip-bottom multiwell plates (cat #89626; ibidi) or on glass coverslips coated with 5 µg/ml fibronectin. After reaching confluency, the monolayers were scratched with a p200 pipette tip and washed with EGM-2. For live imaging and individual cell tracking, Hoechst was added to the cells to label the nuclei (#34580; Thermo Fisher Scientific), and 2 h after scratch wound induction, plates were mounted on an inverted NIKON Eclipse TI microscope equipped with an Okolab cage incubator and humidified gas chamber with environmental settings at 37°C and 5% CO_2_. Phase-contrast and fluorescence imaging of wound closure was performed with 10× CFI Achromat DL dry objective (0.25 NA) and an Andor Zyla 4.2 plus sCMOS camera for 18 h with a time interval of 5–15 min. Scratch wound closure was assessed by taking the percentage of the scratch area that was closed after 7 (Fig. 3) or 9 (Fig. 6) hours of imaging. For the corresponding IF imaging in Fig. S4, c and f, the coverslips were fixed in 4% PFA in PBS++ 6 h after wounding and subsequently immunostained for VE-cadherin, Golgi apparatus, and DAPI.

### Screen localization of BAR-GFP proteins in endothelial cells

The assessment of cellular localization of GFP-tagged BAR proteins was performed using a standardized Lipofectamine transfection protocol. The following day, transfected cells were seeded onto fibronectin-coated glass coverslips at defined densities to obtain reproducibly subconfluent endothelial monolayers at the time of fixation and confocal imaging. Imaging was consistently performed 48 h after transfection to minimize variability in expression levels and cellular state. Only cells within the monolayer were analyzed; isolated cells and dividing cells were excluded. BAR protein association with AJs and other cellular compartments was assessed by manual inspection based on the overlay of GFP (BAR proteins) and VE-cadherin signals. This analysis was performed based on three independent transfections and at least 12 transfected cells per plasmid that displayed clear expression. Only signals that were clearly colocalized with or directly adjacent to VE-cadherin–positive junctions were scored as junction-associated.

### Zebrafish experiments

All animal work adhered to the guidelines of the animal ethics committee at the University of Queensland (Permit, 2022/AE000091). Published transgenic lines used for this work are *Tg(fli1a:H2B-mCherry)*^*uq37bh*^*, Tg(kdrl:EGFP)*^*s843*^ (Baek et al., 2019) and *TgBAC(ve-cad:ve-cadTS)*^*uq11bh*^ (Lagendijk et al., 2017). For live-imaging purposes, all embryos were treated with 0.0003% phenylthiourea from 24 hpf to prevent pigmentation and anesthetized in Tricaine (Sigma-Aldrich E10521-50G, 0.08 mg/ml). To create crispants, we injected 5 nl of guide RNA mix containing 500 ng single-guide RNA duplexes and 10 μg/μl NLS-CAS9 protein (Cat#1081058; IDT) into single-cell stage embryos. After each experiment, embryos were lysed, and a genome PCR was performed to assess targeting efficiency. gRNA and primers for genome PCRs are listed in Table S3. Embryos were staged and cleaned after injections, and sorted for fluorescence at 24–30 hpf. For imaging of the CCV, embryos were mounted in 35-mm glass-bottom dishes or in glass-bottom 6-well plates using 0.7% low-melting point agarose. Confocal *z*-stacks were acquired at 28.5°C on either a Zeiss Axiovert 200 Inverted Microscope with LSM 710 confocal Scanners with BiG detectors using a Plan Apochromat 20 × 0.8 NA dry objective, or a Zeiss Axiovert 200 Inverted Microscope with LSM 880 Confocal Scanner with Fast Airyscan Detector using a Plan Apochromat 40 × 1.2 NA water objective. Embryos were mounted with a tilt so that the tail points upward and the head and upper spine are closest to the bottom to position the CCV in a flat position toward the objective. CCV development was imaged using *Tg(kdrl:EGFP)*^*s843*^ and *Tg(fli1a:H2B-mCherry)*^*uq37bh*^ transgenic embryos between 30 and 48 hpf at 30-min intervals. VE-cadherin dynamics was imaged using *TgBAC*(*ve-cad:ve-cadTS)*^*uq11bh*^ transgenic embryos between 30 and 36 hpf with a time resolution of 45 s to 1 min for 1 h for each embryo. Afterward, Airyscan post-processing and maximum z projections were performed. After imaging experiments, heart-beat and blood flow were assessed to ensure embryo viability was not affected by long-term imaging experiments.

### Image analysis and quantification

Image processing and analysis were performed in Imaris version 10.2, Fiji version 2.9.0, and Ilastik version 1.4.0.

### In vitro experiments

#### Wound closure

FIJI was used to manually draw an ROI in the empty wound area at 0 and 7 or 9 h post-scratch induction, and wound closure percentage was calculated as wound area at the corresponding endpoint divided by the initial wound area.

#### Golgi orientation

Coverslips with shRNA-treated HUVECs were imaged, and angles between the Golgi and nucleus were measured using the angle tool in FIJI. Only the follower cells up to four cell layers behind the leader cells were measured, and the percentage of follower cells in which the Golgi was oriented within 120° toward the migration front was calculated for each image.

#### Colocalization analysis

The colocalization of SNX9-GFP and BIN1-GFP with PACSIN2, EHD4, and MICAL-L1 or the colocalization of dynamin-2 with SNX9 or PACSIN2 was analyzed by taking AAJs larger than 1 um and thresholding both channels of interest. Colocalization was calculated as the Mander’s split coefficient between the thresholded protein of interest (BAR-GFP or dynamin-2) and that of thresholded PACSIN2, EHD4, MICAL-L1, or SNX9.

#### Spatiotemporal recruitment dynamics of BAR-GFP at AAJs

BOECs expressing VE-cadherin–mScarlet with GFP-SNX9, BIN1-GFP, or PACSIN2-GFP were seeded at subconfluency on MatTek imaging dishes (MATTEK P35G-1.5-14-C) and live-imaged at 6 s time-interval. From these videos, the GFP-spots were segmented by background subtraction (radius 10 rolling ball), Gaussian blur, and thresholding (Intermodes). TrackMate (settings: Label image detector, nearest neighbor tracking linking distance = 1) was used to track individual BAR spots, and TrackScheme was used for manual curation of BAR protein spots at AAJs to generate a dataset for spot residency times. Every spot at AAJs was classified according to the subjunctional distribution and for its correlation with a junctional remodeling event.

#### AAJ morphology assessment

Junctions were manually classified in EC monolayers to be straight or bifurcating and counted for the total AAJs classified in this experiment.

#### AAJ protein localization

For every experiment that determined the percentage of AAJs positive for the indicated protein, the percentage of AAJs positive for SNX9, PACSIN2, and dynamin-2 of total AAJs was calculated for each cell.

#### AAJ protein temporal analysis

Time-lapse movies were converted to hyperstacks in Fiji, and individual AAJs with BAR recruitment events were manually selected; for each event, a ROI was drawn over the junction and mean fluorescence intensity over time was extracted for both PACSIN2 and BIN1/SNX9 channels using Z-axis profiling. Recruitment time for each protein was defined as the first frame showing a clear increase above baseline, and the temporal delay (Δt) between BIN1/SNX9 and PACSIN2 arrival at the AAJ was calculated.

#### AAJ lifetime and length

AAJ lifetime and length were quantified from time-lapse movies acquired at 30- or 60-s intervals. For each AAJ, the first frame in which the junction appeared and the last frame in which it disappeared were manually identified, and junction lifetime was calculated as the difference between the last and first frame multiplied by the acquisition interval of the corresponding movie. Maximum AAJ length was measured in FIJI using the line tool by manually selecting the frame in which each AAJ reached its greatest apparent length. Only AAJs that both appeared and disappeared within the imaging period were included in the analysis to ensure complete lifetime measurements.

### In vivo experiments

#### Shape analysis

To analyze CCV shape parameters, a custom FIJI script was used to automatically extract CCV length, perimeter, and area from each timeframe from the maximum projections. In short, the script rotated the images such that the anterior and posterior cardinal veins were horizontal, converted to grayscale, and inverted. The *kdrl:EGFP* channel was thresholded (settings 5, 255), and manual lines were drawn separating the PCV and ACV from the CCV. The script then used Particle Analyzer to extract CCVs as ROIs in each timeframe from which shape parameters, area, length, perimeter were calculated. To correct shape parameters for variations in experimental timing, we aligned the timings between individual experiments by calculating a general growth rate for the uninjected control embryos and then took the maximum area, perimeter and height for the recalculated 40 hpf.

#### CCV analysis and cell tracking

Maximum widths of the leading edge of temporally aligned CCVs at 40 hpf were measured by manually drawing a line between the leftmost and rightmost cell of the leading edge of the CCV using FIJI. Cells were tracked in Imaris for 8 h between 31 and 39 hpf. CCVs were rotated and aligned in time with the reference frame tool to correct for embryo movement. The spot analysis tool was used to select nuclei on the *kdrl:EGFP* channel, and track analysis with manual curation was used to create cell trajectories. Cell trajectories were classified based on their starting location as leader cells, follower cells, dorsal follower cells, or lateral cells. Cells that underwent division were excluded from analysis.

#### Junction straightness

Junction straightness was quantified from the first timeframe of representative time-lapse Airyscan confocal sequences of *Tg*(*ve-cad:ve-cadTS)*^*uq11bh*^ embryos. In FIJI, the segmented line tool was used to manually trace the junction between two neighboring cells to measure the total junctional distance. Absolute junctional distance was measured using the straight line tool between the junctional vertices. Straightness was calculated by the ratio of absolute distance divided by the total distance. Only junctions from cells in the CCV monolayer were quantified.

### Statistical analysis

Graphs and statistical analyses were performed using GraphPad Prism version 10 and RStudio version 13 with R version 3.6.0. Datasets were assessed for normality using histograms and Q–Q plots. The statistical tests used for each analysis are specified in the corresponding figure legends.

### Online supplemental material

Fig. S1 shows Cryo-SIM, FIB-SEM, and curvatures of endothelial AAJs. Fig. S2 shows localization of BAR-GFP proteins in endothelial cells. Fig. S3 shows BAR protein expression in endothelial cells. Fig. S4 shows validation of shRNAs for BAR proteins, BAR-GFP overexpression, and DNM2 knockdowns. Fig. S5 shows Snx9 alignment, knockout efficiency of gRNA pools, and CCV migration parameters. Video 1 shows a FIB-SEM overview of BOECs. Video 2 shows a traverse across the z-axis EM + SIM overlay of AAJ 1–3. Video 3 shows 3D membrane renderings of AAJ 1–3. Video 4 shows the effect of BAR protein knockdowns on collective EC migration. Video 5 shows BAR protein recruitment and AAJ remodeling. Video 6 shows stages of AAJ remodeling. Video 7 shows BIN1 and PACSIN2 recruitment to AAJs. Video 8 shows SNX9 and PACSIN2 recruitment to AAJs. Video 9 shows that SNX9 at AAJs is derived from one side. Video 10 shows dynamin-2 recruitment to AAJs. Video 11 shows that SNX9 controls VE-cadherin turnover at AAJs. Video 12 shows dynamin-2 controls VE-cadherin turnover at AAJs. Video 13 shows the effect of SNX9 and DNM2 knockdowns on collective EC migration. Video 14 shows *Snx9ab* depletion deregulates EC migration in the CCV. Video 15 shows *Snx9ab* depletion alters VE-cadherin remodeling in the CCV. Table S1 lists BAR proteins screened for localization at AAJs and other compartments in endothelial cells in three independent experiments. Table S2 shows unique identifiers of the shRNA constructs from TRC. Table S3 shows gRNA sequences used to knock out genes in zebrafish and HRMA primers.

## Supplementary Material

Review History

Table S1 lists BAR proteins screened for localization at AAJs and other compartments in endothelial cells in three independent experiments.

Table S2 shows unique identifiers of the shRNA constructs from TRC.

Table S3 shows gRNA sequences used to knock out genes in zebrafish and HRMA primers.

SourceData F6 is the source file for Fig. 6.

SourceData FS4 is the source file for Fig. S4.

SourceData FS5 is the source file for Fig. S5.

## Data Availability

Source images and other data are available from the corresponding author, Stephan Huveneers (s.huveneers-at-amsterdamumc.nl), upon reasonable request.

## References

[bib1] Angulo-Urarte, A., T. van der Wal, and S. Huveneers. 2020. Cell-cell junctions as sensors and transducers of mechanical forces. Biochim. Biophys. Acta Biomembr. 1862:183316. 10.1016/j.bbamem.2020.183316 32360073

[bib2] Arima, S., K. Nishiyama, T. Ko, Y. Arima, Y. Hakozaki, K. Sugihara, H. Koseki, Y. Uchijima, Y. Kurihara, and H. Kurihara. 2011. Angiogenic morphogenesis driven by dynamic and heterogeneous collective endothelial cell movement. Development. 138:4763–4776. 10.1242/dev.068023 21965612

[bib3] Baek, S., T.G. Oh, G. Secker, D.L. Sutton, K.S. Okuda, S. Paterson, N.I. Bower, J. Toubia, K. Koltowska, S.J. Capon, . 2019. The alternative splicing regulator Nova2 constrains vascular erk signaling to limit specification of the lymphatic lineage. Dev. Cell. 49:279–292.e275. 10.1016/j.devcel.2019.03.017 31014480

[bib4] Bentley, K., C.A. Franco, A. Philippides, R. Blanco, M. Dierkes, V. Gebala, F. Stanchi, M. Jones, I.M. Aspalter, G. Cagna, . 2014. The role of differential VE-cadherin dynamics in cell rearrangement during angiogenesis. Nat. Cell Biol. 16:309–321. 10.1038/ncb2926 24658686

[bib5] Bentley, K., G. Mariggi, H. Gerhardt, and P.A. Bates. 2009. Tipping the balance: Robustness of tip cell selection, migration and fusion in angiogenesis. PLoS Comput. Biol. 5:e1000549. 10.1371/journal.pcbi.1000549 19876379 PMC2762315

[bib6] Bharathan, N.K., W. Giang, C.L. Hoffman, J.S. Aaron, S. Khuon, T.-L. Chew, S. Preibisch, E.T. Trautman, L. Heinrich, J. Bogovic, . 2023. Architecture and dynamics of a desmosome–endoplasmic reticulum complex. Nat. Cell Biol. 25:823–835. 10.1038/s41556-023-01154-4 37291267 PMC10960982

[bib7] Brevier, J., D. Montero, T. Svitkina, and D. Riveline. 2008. The asymmetric self-assembly mechanism of adherens junctions: A cellular push-pull unit. Phys. Biol. 5:016005. 10.1088/1478-3975/5/1/016005 18379019

[bib8] Carmeliet, P., and D. Collen. 2000. Molecular basis of angiogenesis. Role of VEGF and VE-cadherin. Ann. N. Y. Acad. Sci. 902:249–262. 10.1111/j.1749-6632.2000.tb06320.x 10865845

[bib9] Carmeliet, P., M.G. Lampugnani, L. Moons, F. Breviario, V. Compernolle, F. Bono, G. Balconi, R. Spagnuolo, B. Oosthuyse, M. Dewerchin, . 1999. Targeted deficiency or cytosolic truncation of the VE-cadherin gene in mice impairs VEGF-Mediated endothelial survival and angiogenesis. Cell. 98:147–157. 10.1016/s0092-8674(00)81010-7 10428027

[bib10] Carvalho, J.R., I.C. Fortunato, C.G. Fonseca, A. Pezzarossa, P. Barbacena, M.A. Dominguez-Cejudo, F.F. Vasconcelos, N.C. Santos, F.A. Carvalho, and C.A. Franco. 2019. Non-canonical Wnt signaling regulates junctional mechanocoupling during angiogenic collective cell migration. Elife. 8:e45853. 10.7554/eLife.45853 31246175 PMC6684320

[bib60] Casamento, A., and E. Boucrot. 2020. Molecular mechanism of Fast Endophilin-Mediated Endocytosis. Biochem. J. 477:2327–2345. 10.1042/BCJ20190342 32589750 PMC7319585

[bib27] Chan Wah Hak, L., S. Khan, I.D. Meglio, A.L. Law, S.L.A. Häsler, L.M. Quintaneiro, A.P.A. Ferreira, M. Krause, H.T. McMahon, and E. Boucrot. 2018. Fbp17 and cip4 recruit ship2 and lamellipodin to prime the plasma membrane for fast endophilin-mediated endocytosis. Nat. Cell Biol. 20:1023–1031. 10.1038/s41556-018-0146-8 30061681 PMC6122583

[bib11] Claxton, D. 2026. Surface curvature. In MATLAB Central File Exchange. https://www.mathworks.com/matlabcentral/fileexchange/11168-surface-curvature. Retrieved July 16, 2026.

[bib12] Collins, C., and W.J. Nelson. 2015. Running with neighbors: Coordinating cell migration and cell-cell adhesion. Curr. Opin. Cell Biol. 36:62–70. 10.1016/j.ceb.2015.07.004 26201843 PMC4639402

[bib13] Cowling, B.S., I. Prokic, H. Tasfaout, A. Rabai, F. Humbert, B. Rinaldi, A.S. Nicot, C. Kretz, S. Friant, A. Roux, and J. Laporte. 2017. Amphiphysin (BIN1) negatively regulates dynamin 2 for normal muscle maturation. J. Clin. Invest. 127:4477–4487. 10.1172/JCI90542 29130937 PMC5707152

[bib14] Dorland, Y.L., T.S. Malinova, A.M.D. Van Stalborch, A.G. Grieve, D. Van Geemen, N.S. Jansen, B.J. De Kreuk, K. Nawaz, J. Kole, D. Geerts, . 2016. The F-BAR protein pacsin2 inhibits asymmetric VE-cadherin internalization from tensile adherens junctions. Nat. Commun. 7:12210. 10.1038/ncomms12210 27417273 PMC4947187

[bib15] Dräger, N.M., E. Nachman, M. Winterhoff, S. Bruhmann, P. Shah, T. Katsinelos, S. Boulant, A.A. Teleman, J. Faix, and T.R. Jahn. 2017. Bin1 directly remodels actin dynamics through its BAR domain. EMBO Rep. 18:2051–2066. 10.15252/embr.201744137 28893863 PMC5666605

[bib16] Efimova, N., and T.M. Svitkina. 2018. Branched actin networks push against each other at adherens junctions to maintain cell-cell adhesion. J. Cell Biol. 217:1827–1845. 10.1083/jcb.201708103 29507127 PMC5940301

[bib17] Ferreira, A.P.A., A. Casamento, S. Carrillo Roas, E.F. Halff, J. Panambalana, S. Shaan, K. Schützenhofer, L. Chan Wah Hak, K. McGourty, K. Thalassinos, . 2021. CDK5 and GSK3β inhibit fast endophilin-mediated endocytosis. Nat. Commun. 12:2424. 10.1038/s41467-021-22603-4 33893293 PMC8065113

[bib18] Friedl, P., and D. Gilmour. 2009. Collective cell migration in morphogenesis, regeneration and cancer. Nat. Rev. Mol. Cell Biol. 10:445–457. 10.1038/nrm2720 19546857

[bib19] Friedl, P., and R. Mayor. 2017. Tuning collective cell migration by cell-cell junction regulation. Cold Spring Harb Perspect. Biol. 9:a029199. 10.1101/cshperspect.a029199 28096261 PMC5378050

[bib20] Fujise, K., S. Noguchi, and T. Takeda. 2022. Centronuclear myopathy caused by defective membrane remodelling of dynamin 2 and BIN1 variants. Int. J. Mol. Sci. 23:6274. 10.3390/ijms23116274 35682949 PMC9181712

[bib21] Fujise, K., M. Okubo, T. Abe, H. Yamada, I. Nishino, S. Noguchi, K. Takei, and T. Takeda. 2021. Mutant BIN1-Dynamin 2 complexes dysregulate membrane remodeling in the pathogenesis of centronuclear myopathy. J. Biol. Chem. 296:100077. 10.1074/jbc.RA120.015184 33187981 PMC7949082

[bib22] Genet, G., K. Boyé, T. Mathivet, R. Ola, F. Zhang, A. Dubrac, J. Li, N. Genet, L. Henrique Geraldo, L. Benedetti, . 2019. Endophilin-A2 dependent VEGFR2 endocytosis promotes sprouting angiogenesis. Nat. Commun. 10:1–15. 10.1038/s41467-019-10359-x 31138815 PMC6538628

[bib23] Gory-Faure, S., M.H. Prandini, H. Pointu, V. Roullot, I. Pignot-Paintrand, M. Vernet, and P. Huber. 1999. Role of vascular endothelial-cadherin in vascular morphogenesis. Development. 126:2093–2102. 10.1242/dev.126.10.2093 10207135

[bib24] Grimsley-Myers, C.M., R.H. Isaacson, C.M. Cadwell, J. Campos, M.S. Hernandes, K.R. Myers, T. Seo, W. Giang, K.K. Griendling, and A.P. Kowalczyk. 2020. VE-cadherin endocytosis controls vascular integrity and patterning during development. J. Cell Biol. 219:e201909081. 10.1083/jcb.201909081 32232465 PMC7199849

[bib25] Gumbiner, B.M., and N.G. Kim. 2014. The Hippo-YAP signaling pathway and contact inhibition of growth. J. Cell Sci. 127:709–717. 10.1242/jcs.140103 24532814 PMC3924201

[bib26] Gustafsson, M.G., L. Shao, P.M. Carlton, C.J. Wang, I.N. Golubovskaya, W.Z. Cande, D.A. Agard, and J.W. Sedat. 2008. Three-dimensional resolution doubling in wide-field fluorescence microscopy by structured illumination. Biophys. J. 94:4957–4970. 10.1529/biophysj.107.120345 18326650 PMC2397368

[bib28] Hamm, M.J., B.C. Kirchmaier, and W. Herzog. 2016. Sema3d controls collective endothelial cell migration by distinct mechanisms via Nrp1 and PlxnD1. J. Cell Biol. 215:415–430. 10.1083/jcb.201603100 27799363 PMC5100291

[bib29] Han, M.K., E. Hoijman, E. Noel, L. Garric, J. Bakkers, and J. de Rooij. 2016. αE-catenin-dependent mechanotransduction is essential for proper convergent extension in zebrafish. Biol. Open. 5:1461–1472. 10.1242/bio.021378 27612508 PMC5087688

[bib30] Hayer, A., L. Shao, M. Chung, L.M. Joubert, H.W. Yang, F.C. Tsai, A. Bisaria, E. Betzig, and T. Meyer. 2016. Engulfed cadherin fingers are polarized junctional structures between collectively migrating endothelial cells. Nat. Cell Biol. 18:1311–1323. 10.1038/ncb3438 27842057 PMC6159904

[bib31] Helker, C.S.M., A. Schuermann, T. Karpanen, D. Zeuschner, H.G. Belting, M. Affolter, S. Schulte-Merker, and W. Herzog. 2013. The zebrafish common cardinal veins develop by a novel mechanism: Lumen ensheathment. Development. 140:2776–2786. 10.1242/dev.091876 23698350

[bib32] Hoffman, D.P., G. Shtengel, C.S. Xu, K.R. Campbell, M. Freeman, L. Wang, D.E. Milkie, H.A. Pasolli, N. Iyer, J.A. Bogovic, . 2020. Correlative three-dimensional super-resolution and block-face electron microscopy of whole vitreously frozen cells. Science. 367:eaaz5357. 10.1126/science.aaz5357 31949053 PMC7339343

[bib33] Huveneers, S., J. Oldenburg, E. Spanjaard, G. van der Krogt, I. Grigoriev, A. Akhmanova, H. Rehmann, and J. de Rooij. 2012. Vinculin associates with endothelial VE-cadherin junctions to control force-dependent remodeling. J. Cell Biol. 196:641–652. 10.1083/jcb.201108120 22391038 PMC3307691

[bib34] Janssen, V., and S. Huveneers. 2024. Cell-cell junctions in focus - Imaging junctional architectures and dynamics at high resolution. J. Cell Sci. 137:jcs262041. 10.1242/jcs.262041 39480660

[bib35] Jones, R.C., J. Karkanias, M.A. Krasnow, A.O. Pisco, S.R. Quake, J. Salzman, N. Yosef, B. Bulthaup, P. Brown, W. Harper, . 2022. The Tabula Sapiens: A multiple-organ, single-cell transcriptomic atlas of humans. Science. 376:eabl4896. 10.1126/science.abl4896 35549404 PMC9812260

[bib36] Kaksonen, M., and A. Roux. 2018. Mechanisms of clathrin-mediated endocytosis. Nat. Rev. Mol. Cell Biol. 19:313–326. 10.1038/nrm.2017.132 29410531

[bib37] Khalil, A.A., and J. de Rooij. 2019. Cadherin mechanotransduction in leader-follower cell specification during collective migration. Exp. Cell Res. 376:86–91. 10.1016/j.yexcr.2019.01.006 30633881

[bib38] Kim, N.G., E. Koh, X. Chen, and B.M. Gumbiner. 2011. E-cadherin mediates contact inhibition of proliferation through Hippo signaling-pathway components. Proc. Natl. Acad. Sci. USA. 108:11930–11935. 10.1073/pnas.1103345108 21730131 PMC3141988

[bib39] Kreider-Letterman, G., A. Castillo, E.K. Mahlandt, J. Goedhart, A. Rabino, S. Goicoechea, and R. Garcia-Mata. 2023. ARHGAP17 regulates the spatiotemporal activity of Cdc42 at invadopodia. J. Cell Biol. 222:e202207020. 10.1083/jcb.202207020 36571786 PMC9794838

[bib40] Lagendijk, A.K., G.A. Gomez, S. Baek, D. Hesselson, W.E. Hughes, S. Paterson, D.E. Conway, H.G. Belting, M. Affolter, K.A. Smith, . 2017. Live imaging molecular changes in junctional tension upon VE-cadherin in zebrafish. Nat. Commun. 8:1402. 10.1038/s41467-017-01325-6 29123087 PMC5680264

[bib41] Lowe, D.G. 2004. Distinctive image features from scale-invariant keypoints. Int. J. Comput. Vis. 60:91–110. 10.1023/b:visi.0000029664.99615.94

[bib42] Mahlandt, E.K., S. Palacios Martinez, J.J.G. Arts, S. Tol, J.D. van Buul, and J. Goedhart. 2023. Opto-RhoGEFs, an optimized optogenetic toolbox to reversibly control Rho GTPase activity on a global to subcellular scale, enabling precise control over vascular endothelial barrier strength. Elife. 12:RP84364. 10.7554/eLife.84364 37449837 PMC10393062

[bib43] Malinova, T.S., A. Angulo-Urarte, J. Nüchel, M. Tauber, M.M. van der Stoel, V. Janssen, A. de Haan, A.G. Groenen, M. Tebbens, M. Graupera, . 2021. A junctional PACSIN2/EHD4/MICAL-L1 complex coordinates VE-cadherin trafficking for endothelial migration and angiogenesis. Nat. Commun. 12:2610. 10.1038/s41467-021-22873-y 33972531 PMC8110786

[bib44] Malinova, T.S., and S. Huveneers. 2018. Sensing of cytoskeletal forces by asymmetric adherens junctions. Trends Cell Biol. 28:328–341. 10.1016/j.tcb.2017.11.002 29195724

[bib45] Root, D.E., N. Hacohen, W.C. Hahn, E.S. Lander, and D.M. Sabatini. 2006. Genome-scale loss-of-function screening with a lentiviral RNAi library. Nat. Methods. 3:715–719. 10.1038/nmeth924 16929317

[bib46] Schöneberg, J., M. Lehmann, A. Ullrich, Y. Posor, W.T. Lo, G. Lichtner, J. Schmoranzer, V. Haucke, and F. Noé. 2017. Lipid-mediated PX-BAR domain recruitment couples local membrane constriction to endocytic vesicle fission. Nat. Commun. 8:15873. 10.1038/ncomms15873 28627515 PMC5481832

[bib47] Simunovic, M., E. Evergren, A. Callan-Jones, and P. Bassereau. 2019. Curving cells inside and out: Roles of BAR domain proteins in membrane shaping and its cellular implications. Annu. Rev. Cell Dev. Biol. 35:111–129. 10.1146/annurev-cellbio-100617-060558 31340125

[bib59] Senju, Y., E. Rosenbaum, C. Shah, S. Hamada-Nakahara, Y. Itoh, K. Yamamoto, K. Hanawa-Suetsugu, O. Daumke, and S. Suetsugu. 2015. Phosphorylation of PACSIN2 by protein kinase C triggers the removal of caveolae from the plasma membrane. J. Cell Sci. 128:2766–2780. 10.1242/jcs.167775 26092940

[bib48] Simunovic, M., G.A. Voth, A. Callan-Jones, and P. Bassereau. 2015. When physics takes over: BAR proteins and membrane curvature. Trends Cell Biol. 25:780–792. 10.1016/j.tcb.2015.09.005 26519988 PMC4831700

[bib49] Szymborska, A., and H. Gerhardt. 2018. Hold me, but not too tight—endothelial cell–cell junctions in angiogenesis. Cold Spring Harbor Perspect. Biol. 10:a029223. 10.1101/cshperspect.a029223 PMC6071488 28851748

[bib50] Taylor, M.J., D. Perrais, and C.J. Merrifield. 2011. A high precision survey of the molecular dynamics of mammalian clathrin-mediated endocytosis. PLoS Biol. 9:e1000604. 10.1371/journal.pbio.1000604 21445324 PMC3062526

[bib51] Trepat, X., M.R. Wasserman, T.E. Angelini, E. Millet, D.A. Weitz, J.P. Butler, and J.J. Fredberg. 2009. Physical forces during collective cell migration. Nat. Phys. 5:426–430. 10.1038/nphys1269

[bib52] Vitorino, P., and T. Meyer. 2008. Modular control of endothelial sheet migration. Genes Dev. 22:3268–3281. 10.1101/gad.1725808 19056882 PMC2600767

[bib53] Wang, Q., M.V.A.S. Navarro, G. Peng, E. Molinelli, S.L. Goh, B.L. Judson, K.R. Rajashankar, and H. Sondermann. 2009. Molecular mechanism of membrane constriction and tubulation mediated by the F-BAR protein Pacsin/Syndapin. Proc. Natl. Acad. Sci. USA. 106:12700–12705. 10.1073/pnas.0902974106 19549836 PMC2722316

[bib54] Wu, R.S., I.I. Lam, H. Clay, D.N. Duong, R.C. Deo, and S.R. Coughlin. 2018. A rapid method for directed gene knockout for screening in G0 zebrafish. Dev. Cell. 46:112–125.e4. 10.1016/j.devcel.2018.06.003 29974860

[bib55] Xu, C.S., K.J. Hayworth, Z. Lu, P. Grob, A.M. Hassan, J.G. Garcia-Cerdan, K.K. Niyogi, E. Nogales, R.J. Weinberg, and H.F. Hess. 2017. Enhanced FIB-SEM systems for large-volume 3D imaging. Elife. 6:e25916. 10.7554/eLife.25916 28500755 PMC5476429

[bib56] Yap, A.S., K. Duszyc, and V. Viasnoff. 2018. Mechanosensing and mechanotransduction at cell-cell junctions. Cold Spring Harb. Perspect. Biol. 10:a028761. 10.1101/cshperspect.a028761 28778874 PMC6071489

[bib57] Yarar, D., C.M. Waterman-Storer, and S.L. Schmid. 2007. SNX9 couples actin assembly to phosphoinositide signals and is required for membrane remodeling during endocytosis. Dev. Cell. 13:43–56. 10.1016/j.devcel.2007.04.014 17609109

[bib58] Yuge, S., K. Nishiyama, Y. Arima, Y. Hanada, E. Oguri-Nakamura, S. Hanada, T. Ishii, Y. Wakayama, U. Hasegawa, K. Tsujita, . 2022. Mechanical loading of intraluminal pressure mediates wound angiogenesis by regulating the TOCA family of F-BAR proteins. Nat. Commun. 13:2594. 10.1038/s41467-022-30197-8 35551172 PMC9098626

