# Supplementary material for: Nanoscale junctional membrane curvatures recruit BIN1 and SNX9 for endothelial collective migration
Source: J Cell Biol. 2026 Jul 27;225(9):e202509207. doi: 10.1083/jcb.202509207 (PMC13404086; doi:10.1083/jcb.202509207)
Supplement: Table S2 — shows unique identifiers of the shRNA constructs from TRC. [file jcb_202509207_tables2.docx]

**Table 2. Unique identifiers of the short hairpin RNA (shRNA) constructs from The RNAi Consortium (TRC).**

| **GeneNumber** | **TRC** |
| --- | --- |
| ARHGAP17-1 | TRCN0000047450 |
| ARHGAP17-2 | TRCN0000047452 |
| ASAP1-2 | TRCN0000123118 |
| BIN1-1 | TRCN0000118039 |
| BIN1-2 | TRCN0000118041 |
| BIN3-1 | TRCN0000129928 |
| BIN3-2 | TRCN0000130975 |
| DNM2-1 | TRCN0000006648 |
| DNM2-2 | TRCN0000006649 |
| FER-1 | TRCN0000002347 |
| FER-2 | TRCN0000002348 |
| FER-3 | TRCN0000002349 |
| FER-4 | TRCN0000002350 |
| FER-5 | TRCN0000002351 |
| PACSIN2 | TRCN0000037980 |
| PICK1-1 | TRCN0000037905 |
| PICK1-2 | TRCN0000037907 |
| SH3GL1-1 | TRCN0000083923 |
| SH3GL1-2 | TRCN0000083926 |
| shControl | shCOO2 |
| SNX9-1 | TRCN0000147249 |
| SNX9-2 | TRCN0000149857 |
| SNX9-3 | TRCN0000417815 |
