# Supplementary material for: Nanoscale junctional membrane curvatures recruit BIN1 and SNX9 for endothelial collective migration
Source: J Cell Biol. 2026 Jul 27;225(9):e202509207. doi: 10.1083/jcb.202509207 (PMC13404086; doi:10.1083/jcb.202509207)
Supplement: Table S3 — shows gRNA sequences used to knock out genes in zebrafish and HRMA primers. [file jcb_202509207_tables3.docx]

**Table 3. gRNA sequences used to knockout genes in zebrafish and HRMA primers.**

| GENE | Application | Sequence |
| --- | --- | --- |
| pacsin2 | gRNA Target 1 | TAATACGACTCACTATAGGCAGGCCCTCAGTACGGGAGTTTTAGAGCTAGAAATAGC |
|  | gRNA Target 2 | TAATACGACTCACTATAGGCGGAGGACGGCTTTCGCAGTTTTAGAGCTAGAAATAGC |
|  | gRNA Target 3 | TAATACGACTCACTATAGGGTGGTTCCGCTCAAACCAGTTTTAGAGCTAGAAATAGC |
|  | gRNA Target 4 | TAATACGACTCACTATAGGACCGGACCCTCAGCCGCCGTTTTAGAGCTAGAAATAGC |
| snx9a | gRNA Target 1 | TAATACGACTCACTATAGGCATTGATGCCCGCAATCAGTTTTAGAGCTAGAAATAGC |
|  | gRNA Target 2 | TAATACGACTCACTATAGGAAAGTCGACTGGTGGTTAGTTTTAGAGCTAGAAATAGC |
|  | gRNA Target 3 | TAATACGACTCACTATAGGAACTAGGGAAGCGGAAAGGTTTTAGAGCTAGAAATAGC |
|  | gRNA Target 4 | TAATACGACTCACTATAGGTCTTACTTGGGTATCCACGTTTTAGAGCTAGAAATAGC |
| snx9b | gRNA Target 1 | TAATACGACTCACTATAGGGATTTCGCGGCAGAGCCTGTTTTAGAGCTAGAAATAGC |
|  | gRNA Target 2 | TAATACGACTCACTATAGGGAGCTACATCCACCTTAGGTTTTAGAGCTAGAAATAGC |
|  | gRNA Target 3 | TAATACGACTCACTATAGGGTTTGACAATAATGCCTCGTTTTAGAGCTAGAAATAGC |
|  | gRNA Target 4 | TAATACGACTCACTATAGGGCATCCACAGGCCTACCAGTTTTAGAGCTAGAAATAGC |
| pacsin2 | HRMA Forward Target 1 | GGGCTGAATGAGTGTTCTCG |
|  | HRMA Reverse Target 1 | CTTCTCGGCCTCGGTCAT |
|  | HRMA Forward Target 2 | GATGATCGGGGGCTTCAA |
|  | HRMA Reverse Target 2 | GCTGCCTGTGTTTACCTCCT |
|  | HRMA Forward Target 3 | AGGGAGCAGATCCACAAGAG |
|  | HRMA Reverse Target 3 | CTGTCTCCAATGCTCTGTAAATG |
|  | HRMA Forward Target 4 | TGTGTGTGTGCTGCAGGAAT |
|  | HRMA Reverse Target 4 | TGACTCTGGAGCTCTGCTCA |
| snx9a | HRMA Forward Target 1 | GAACTCTGCTTCCGCACAA |
|  | HRMA Reverse Target 1 | CAAATGGTTTTAACTGATGTGG |
|  | HRMA Forward Target 2 | CTTCGATGAGGAATGGGATG |
|  | HRMA Reverse Target 2 | CACTCCGCTGCATGGTTC |
|  | HRMA Forward Target 3 | TGTCGGGTGTGTTGTAAAGC |
|  | HRMA Reverse Target 3 | CAACTCTGGGAGCTCTGGAT |
|  | HRMA Forward Target 4 | GGAGTATCGGCAGATTGGAA |
|  | HRMA Reverse Target 4 | CAAAACTGTTCACAGCTACGG |
| snx9b | HRMA Forward Target 1 | CCCTCACCTGATTGGTTATTG |
|  | HRMA Reverse Target 1 | CCAATGCATGTTTTATTTTTACACA |
|  | HRMA Forward Target 2 | AGGTGAAGCCTGGGGTAACT |
|  | HRMA Reverse Target 2 | AACCCTTCCCTTTCAGGTGT |
|  | HRMA Forward Target 3 | TTACTGGTTTTCCCCGTCTC |
|  | HRMA Reverse Target 3 | TCAGACGAGTAACGGCAATG |
|  | HRMA Forward Target 4 | GCCTTCAAAACATTTGGTTCA |
|  | HRMA Reverse Target 4 | AACAACAATGCATGGCAATC |
