# Supplementary figures and images for: Nanoscale junctional membrane curvatures recruit BIN1 and SNX9 for endothelial collective migration
Source: J Cell Biol. 2026 Jul 27;225(9):e202509207. doi: 10.1083/jcb.202509207 (PMC13404086; doi:10.1083/jcb.202509207)

Source Data Fig. 6

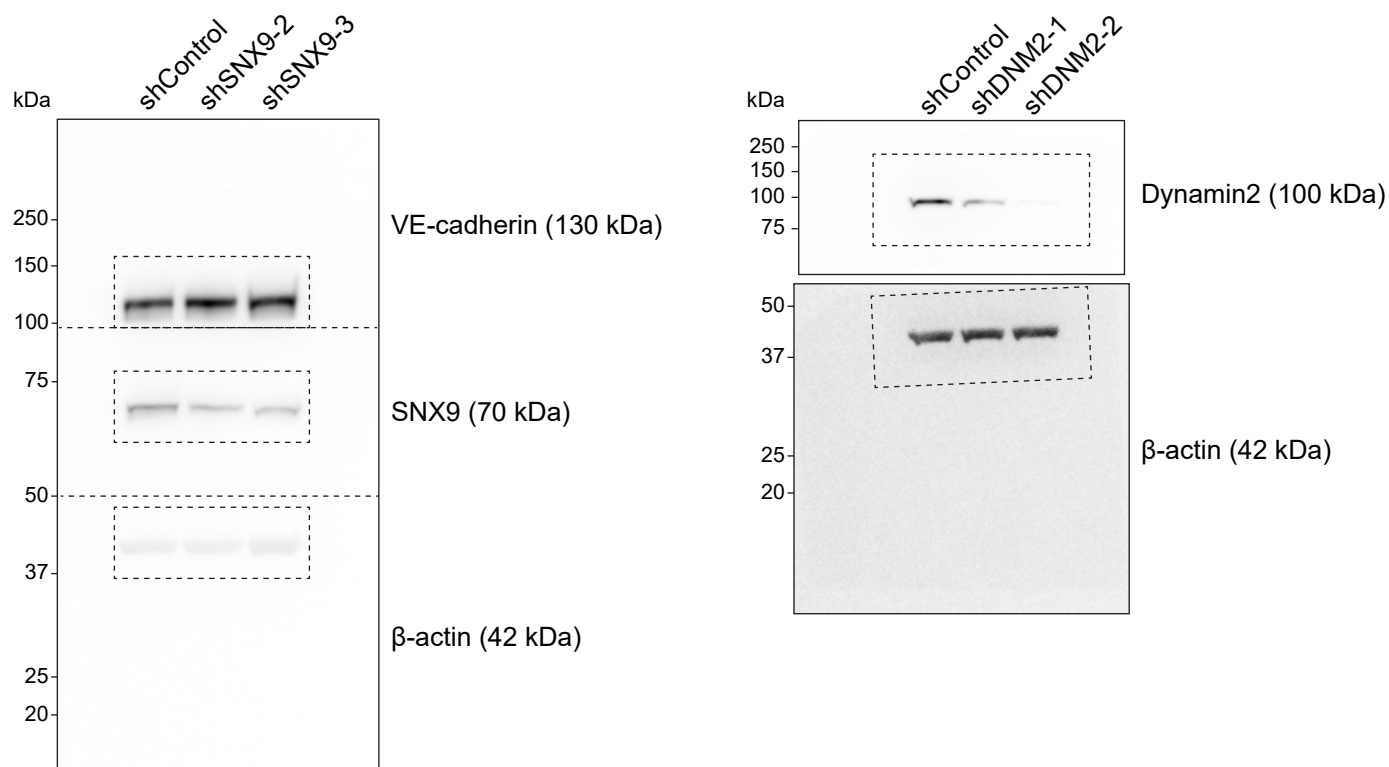

Supplement: SourceData F6 — is the source file for Fig. 6. [file jcb_202509207_sourcedataf6.pdf]

Source Data Supplementary Fig. 4

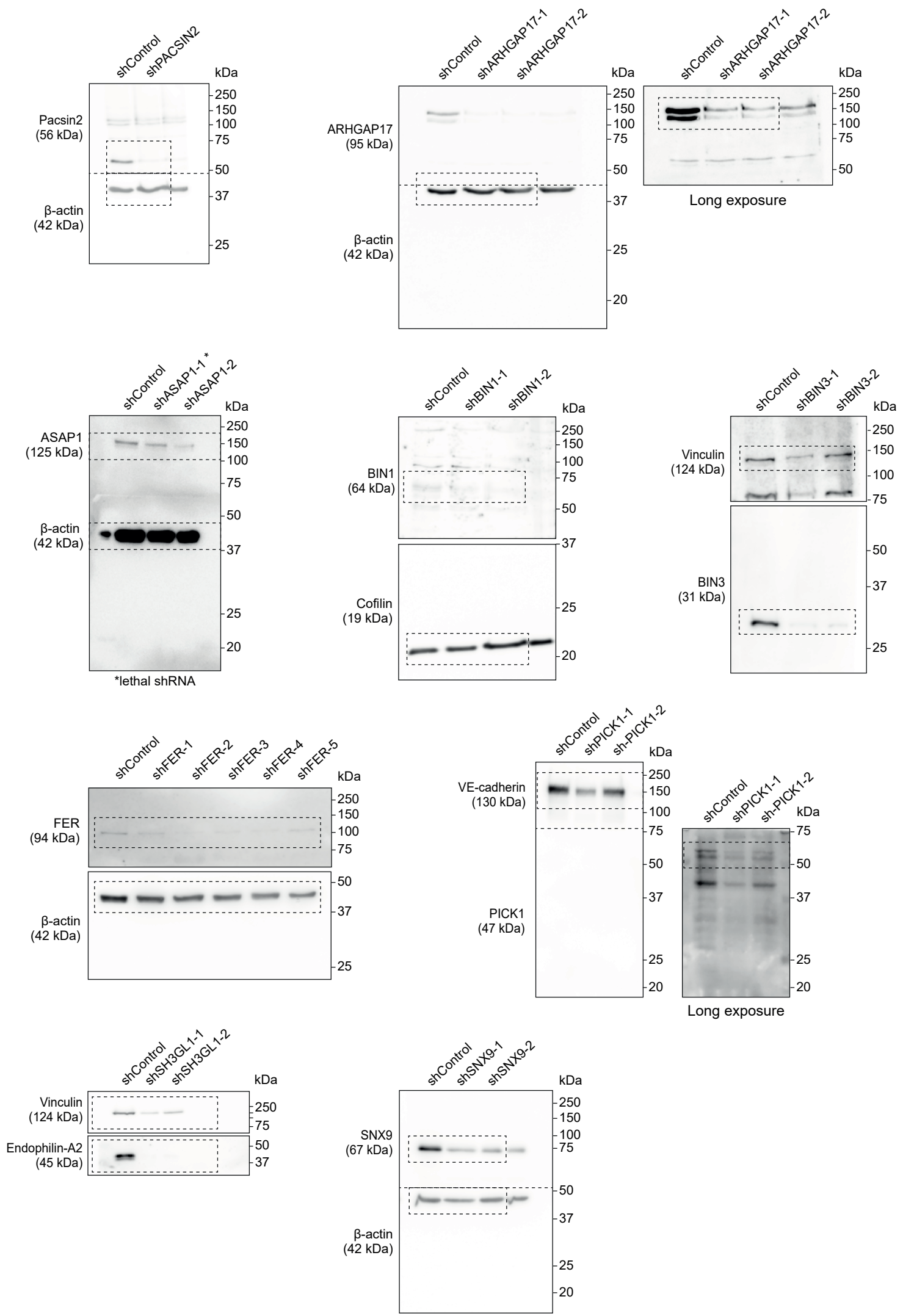

Supplement: SourceData FS4 — is the source file for Fig. S4. [file jcb_202509207_sourcedatafs4.pdf]

Source Data Supplementary Fig. 5

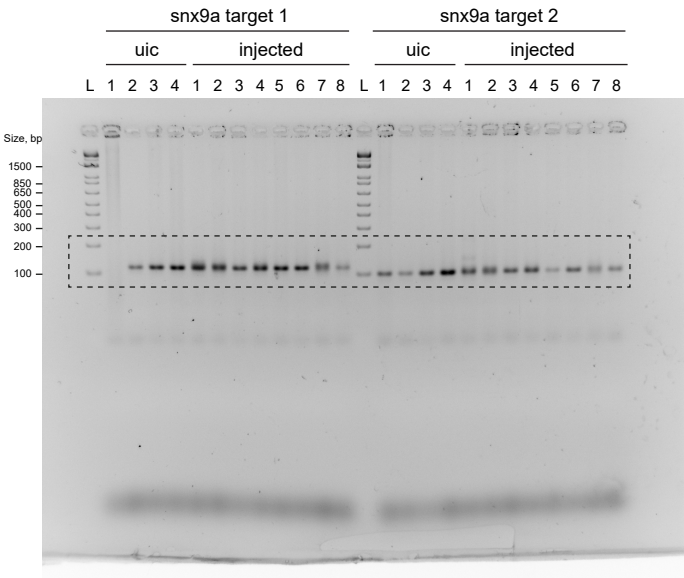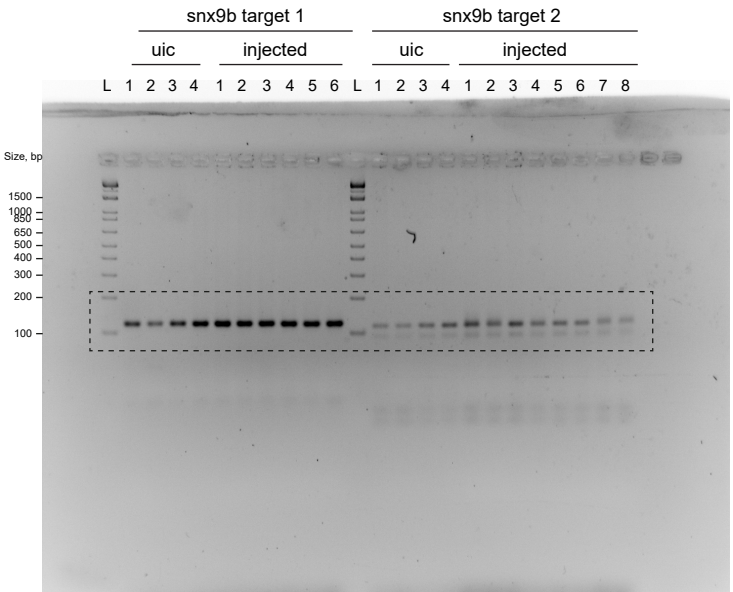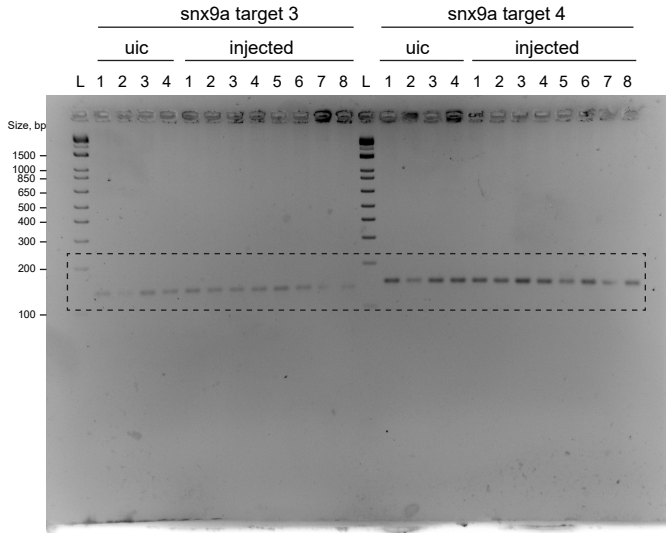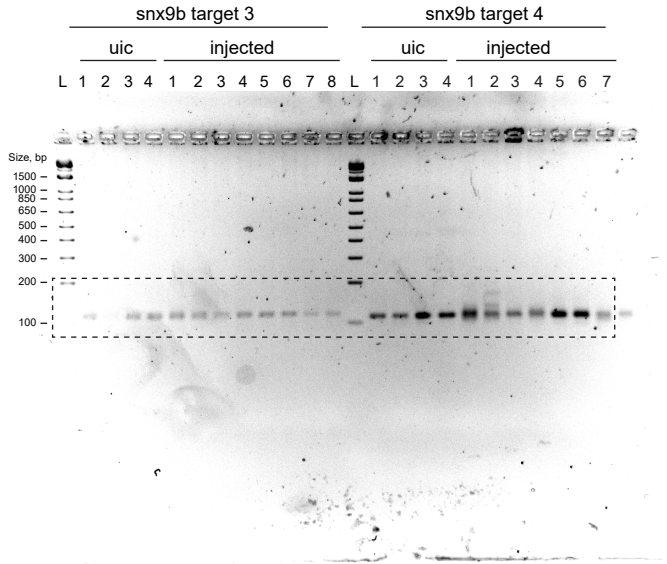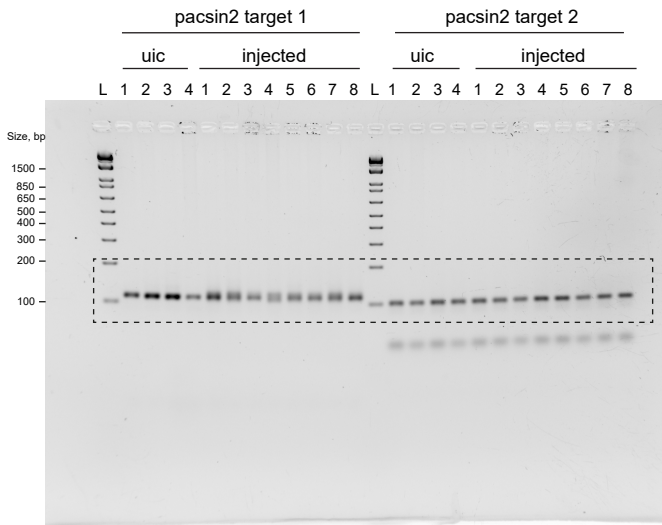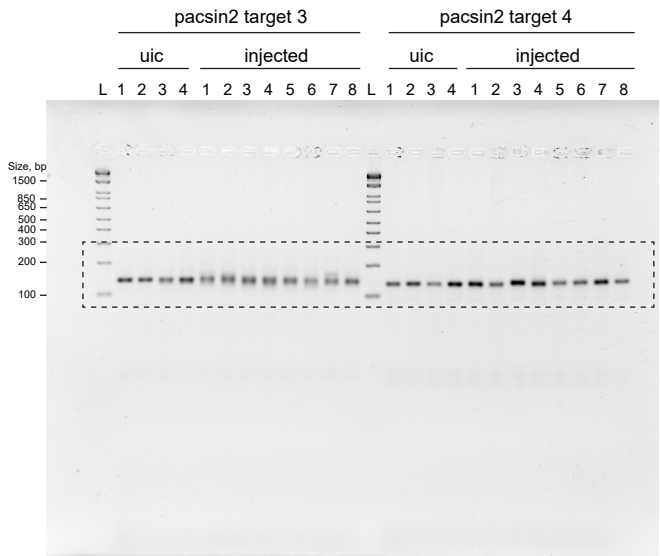

Supplement: SourceData FS5 — is the source file for Fig. S5. [file jcb_202509207_sourcedatafs5.pdf]
