# Supplementary material for: Nanoscale junctional membrane curvatures recruit BIN1 and SNX9 for endothelial collective migration
Source: J Cell Biol. 2026 Jul 27;225(9):e202509207. doi: 10.1083/jcb.202509207 (PMC13404086; doi:10.1083/jcb.202509207)
Supplement: Review History [file jcb_202509207_review_history.pdf]

JCB

Nanoscale junctional membrane curvatures recruit BIN1 and SNX9 for endothelial collective migration

Vera Janssen, Hannah de Kraker, Jesse Aaron, Annett de Haan, Iris de Heer, Satya Khuon, Jason da Silva, Amber Driessen, Teng-Leong Chew, Josephine Tan, Anne Lagendijk, Ana Angulo-Urarte, and Stephan Huveneers

Corresponding Author(s): Stephan Huveneers, Amsterdam University Medical Centers

Review Timeline:

Submission Date:	2025-09-30
Editorial Decision:	2025-10-24
Revision Received:	2026-05-12
Editorial Decision:	2026-06-01
Revision Received:	2026-06-05

Monitoring Editor: Sandrine Etienne-Manneville

Scientific Editor: Gabriele Stephan

Transaction Report:

(Note: With the exception of the correction of typographical or spelling errors that could be a source of ambiguity, letters and reports are not edited. The original formatting of letters and referee reports may not be reflected in this compilation.)

DOI: https://doi.org/10.1083/jcb.202509207

October 24, 2025

Re: JCB manuscript #202509207

Stephan Huveneers
Amsterdam University Medical Centers

Dear Dr. Huveneers,

Thank you for submitting your manuscript entitled "Nanoscale junctional membrane curvatures recruit BIN1 and SNX9 for endothelial collective migration". The manuscript was assessed by expert reviewers, whose comments are appended to this letter. We invite you to submit a revision if you can address the reviewers' key concerns, as outlined here.

You will see that all reviewers are generally supportive, thinking your paper provides significant advances towards the role of specific BAR proteins in asymmetric adherens junctions morphology. They all provide constructive feedback, and we think your manuscript could improve if you were able to address their comments. This includes testing whether the effects of SNX9 and dynamin-2 are dependent (e.g. by using double knockdowns), as recommended by reviewers #1 and #3. All other concerns voiced by the referees should further be addressed.

While you are revising your manuscript, please also attend to the following editorial points to help expedite the publication of your manuscript. Please direct any editorial questions to the journal office.

GENERAL GUIDELINES:

Text limits: Character count for an Article is < 40,000, not including spaces. Count includes title page, abstract, introduction, results, discussion, and acknowledgments. Count does not include materials and methods, figure legends, references, tables, or supplemental legends.

Figures: Articles may have up to 10 main text figures. Figures must be prepared according to the policies outlined in our Instructions to Authors, under Data Presentation, https://jcb.rupress.org/site/misc/ifora.xhtml. All figures in accepted manuscripts will be screened prior to publication.

IMPORTANT: It is JCB policy that if requested, original data images must be made available. Failure to provide original images upon request will result in unavoidable delays in publication. Please ensure that you have access to all original microscopy and blot data images before submitting your revision.

Supplemental information: There are strict limits on the allowable amount of supplemental data. Articles may have up to 5 supplemental figures. Up to 10 supplemental videos or flash animations are allowed. A summary of all supplemental material should appear at the end of the Materials and methods section.

Please note that JCB now requires authors to submit Source Data used to generate figures containing gels and Western blots with all revised manuscripts. This Source Data consists of fully uncropped and unprocessed images for each gel/blot displayed in the main and supplemental figures. For assays performed using capillary electrophoresis and/or immunoassay-based detection, authors should instead provide the electropherogram graph(s) for each experiment, plotting fluorescence/chemiluminescence intensity vs. molecular weight/size. Please be sure to provide one Source Data file for each figure gels, blots, and/or capillary electrophoresis assays along with your revised manuscript files. File names for Source Data figures should be alphanumeric without any spaces or special characters (i.e., SourceDataF#, where F# refers to the associated main figure number or SourceDataFS# for those associated with Supplementary figures). For traditional gels and blots, the lanes of the gels/blots should be labeled as they are in the associated figure, the place where cropping was applied should be marked (with a box), and molecular weight/size standards should be labeled wherever possible. For capillary electrophoresis assays, each trace in the graph should be color-coded and labeled to indicate which protein, gene, or sample is being measured (please try to avoid red/green combinations to accommodate our color-blind readers).

Source Data files will be made available to reviewers during evaluation of revised manuscripts and, if your paper is eventually published in JCB, the files will be directly linked to specific figures in the published article.

Source Data Figures should be provided as individual PDF files (one file per figure). Authors should endeavor to retain a minimum resolution of 300 dpi or pixels per inch. Please review our instructions for export from Photoshop, Illustrator, and PowerPoint here: https://rupress.org/jcb/pages/submission-guidelines#revised

The typical timeframe for revisions is three to four months. If you anticipate any difficulties in meeting this aforementioned revision time limit, please contact us and we can work with you to find an appropriate time frame for resubmission. Please note

that papers are generally considered through only one revision cycle, so any revised manuscript will likely be either accepted or rejected.

When submitting the revision, please include a cover letter addressing the reviewers' comments point by point. Please also highlight all changes in the text of the manuscript.

We hope that the comments below will prove constructive as your work progresses. We would be happy to discuss them further once you've had a chance to consider the points raised in this letter.

Thank you for this interesting contribution to Journal of Cell Biology. You can contact us at the journal office with any questions at cellbio@rockefeller.edu.

Sincerely,

Sandrine Etienne-Manneville
Monitoring Editor
Journal of Cell Biology

Gabriele Stephan
Scientific Editor
Journal of Cell Biology

Reviewer #1:

The authors evaluated asymmetrical adherens junctions (AAJs) between leader and follower cells and their modulation using complex and state-of-the-art techniques including FIB-SEM and high resolution live cell imaging, which enable detailed insights into the ultrastructure and dynamics of these junctions. Further, the authors identify specific BAR proteins as novel regulators of front-to-rear polarity and AAJs in collective cell migration of endothelial cells. The study is interesting and novel by adding insights into the differential contributions of BAR proteins for regulating membrane curvature in AAJs, which profoundly contribute to the process of collective cell migration. Clear strengths of the study are the provision of AAJ 3D reconstructions and the application of different cell culture approaches together with the inclusion of a zebrafish model. However, some weaker aspects remain which should be improved as indicated below.

Major points:

1. In their screen, the authors identified several AAJ-associated bar proteins, selected some of these and tested them further in loss of function studies, demonstrating contributions to collective cell migration. However, it is unclear whether these changes are a result of the BAR proteins localized at the AAJ, as the authors argue, or whether proteins in different localizations are relevant, too. One way to test this would be to select one of the proteins present in endothelial cell but not localized to AAJs and evaluate the functional outcome by silencing approaches. This may serve as excellent negative control, too.
2. Figure 2: How did the authors decide what BARs associate with AAJs? The morphology and size of the cells appears somewhat different in the images provided. Please comment on potential differential states of cells influencing AAJ morphology and BAR association. How did the authors ensure similar states (in terms of density, proliferative stage, quiescence, dividing phase, migratory phase)?
3. Does the AAJ/BAR protein association (at least of those BARs the authors later focus on) also occur under the clear collective cell migration conditions in Figure 3?
4. The temporal relationship of BIN1, SNX9 and PACSIN in Figure 4 is hard to follow based on the images provided in j and k (disclaimer: my Macbook refused to play the .avi videos). How many AAJs/cells were imaged? Please provide an analysis.
5. Similarly, the claim that "dynamin-2 is recruited to AAJs precisely at the moment of junctional scission and internalization" is not comprehensively shown in the image sequence of Fig 5J. Please provide an analysis and a better visualization.
6. Figure 6: Since SNX9 and dynamin-2 are co-recruited and overlap at the AAJ, can the authors show whether the effects of shSNX9 could be rescued by Dynamin-2? Further, is dynamin-2 also required for AAJ stability?
7. Do AAJs also occur in the zebrafish model? Does SNX9 decorate AAJs in the migrating cardinal vein ECs? It would be important to show the relevance of the authors' findings in vivo.

Minor points:
1. The authors use different loading controls for the western blot experiment in Supp figure 4a. Specifically, ARHGAP17 and VE-cadherin are not valid controls since they are not housekeeping proteins, and their expression might be modulated by the knockdown of BAR proteins shown.
2. Supp figure 2: Please provide high magnification images for the junctions to the images provided. How many cells were evaluated to decide if a BAR protein localizes to an AAJ?

3. Please provide exemplary images for the begin and end time points of the results in Figure 3b in the supplements.
4. The legend for PACSIN2 in Figure 4f is not clearly visible with the yellow color. Please adapt.
5. Please provide individual data points for Supplementary Figure 4b and statistical analysis. Are the transcripts normalized to an internal control like GAPDH or actin?
6. The color legends for the figure in Figure S4c is missing.
7. The y-axis is incomplete for Figure S5a.

Reviewer #2:

This manuscript by Janssen et al describes to nanoscale architecure of the asymmetric adherens junctions which are membrane invaginations formed between leader and follower migrating endothelial cells. The localization and distribution of a large number of BAR domain proteins was screened, identifying several of interest. Of these SNX9 and BIN1 were investigated in detail for their distribution and dynamics leading to the discovery that these proteins are required for collective endothelial cell migration. The imaging is excellent and I found the manuscript enjoyable to read. The in vivo experiment is a convincing demonstration of the role of SNX9. My detailed comments are below.

1. Overall, the largest question I have is about which cell, leader or follower, is recruiting the BAR proteins. Given the diversity of membrane curvature, there is positive and negative curvature in the AAJ membrane from each cell. The authors should discuss this concept and provide their thoughts.

2. An experiment to test the contribution of BAR domain proteins in leader vs follower cells would add to the mechanism, but may be beyond the scope and is not required. One could imagine mixing BAR domain expressing and knockdown cells or cells expressing BAR-GFP or BAP-mScarlet and then examining the AAJs between them. This would allow distinction of recruitment and localization from the leader or follower cell.

3. This comment is related to 1. The screen identified many different BAR domain proteins (F-BAR, I-BAR, etc.). Each is known to bind or shape membranes in different ways. Given the different membrane curvatures quantified, can the authors comment on where/which locations the proteins of interest are most likely recruited to?

4. In Figure 1D,E I found the analysis of curvature confusing. For example, some highly curved membranes like the small circles in the bottom of AAJ3 are indicated to have a very low curvature where by eye they seem to have high convex curvature. It may be informative to divide this analysis into convex and concave (or positive and negative) curvature. Currently I interpret the image as showing all convex curvatures denoted as extremely large or tending toward infinity. The authors could also clarify which "side" the curvature is being interpreted from (ie from the trailing cell).

5. In AAJ1and AAJ2 in Fig 1 it appears there are vesicles inside the finger structure. Could these be endocytosed from the leader cell? It seems that as endocytosis happens in the follower cell, a process to balance the membrane area should be happening in the leader cell.

6. Can the authors please note how many junctions were examined by FIBSEM? Is there a population based analysis, perhaps of curvature and VE-Cad or PASCIN2 distribution possible?

Minor Points

Very nice screen of BAR domain proteins. Could the authors describe if there a specific metric used to define if a BAR domain protein was associated and indicate "n" for these screens.

In Fig. 4 The wording around the BAR domain protein distribution in AAJs was somewhat confusing. The definitions of "mid", "over" and "trailing end" are clear in the text and in Fig 4B linescans. The term "subjunctional region" evoked thinking the classes referred to specific locations along the AAJ. Consider "subjunctional distribution" or something similar? Action not required.

Reviewer #3:

In this manuscript, Janssen and colleagues set to expand our understanding on the formation and dynamics of VE-cadherin-based asymmetric adherens junctions (AAJs) in endothelial cells. Using state-of-the-art correlative cryo-SIM/FIB-SEM, they provide a volumetric view of AAJs and reveal pronounced curvature heterogeneity. Building on this, the authors utilize a screen of BAR-domain proteins and identify several AAJ-associated factors with functional impact on collective migration. Among these, BIN1 and SNX9 are developed further, with spatiotemporally distinct localization and evidence that SNX9 contributes to

junctional turnover in vitro and collective migration in vivo. Together, the study extends our understanding of polarized endothelial junction behaviour and points to a sequential machinery that sculpts highly curved and functional structures, with potential relevance beyond endothelial biology.
It is also a technical tour de force.
That said, several parts need clearer explanation or occasionally additional support for some of the main claims. Most notably, the screen and how BAR-protein 'hits' and 'non-hits' were filtered should be spelled out so readers don't mistake technical negatives for biological ones. The link between the impressive volumetric imaging and the later assignment of new AAJ proteins could also be tightened by re-use of established methodology. Finally, a few narrative jumps (e.g., choosing GOI-rich junctions, possible overexpression bias) make some results read stronger than they're currently controlled for. With them addressed, the study would make a solid and broadly useful contribution. Below I list major and minor points by chapter/figure, followed by notes on discussion and methods.

Multiple BAR proteins are recruited to asymmetric adherens junctions
(Fig. 2a-b, 2c; Table 1; Supp. Figs 2-4)

Major

• Screen sampling and basic tallies. It could help readers if the library screen included simple counts - how many cells/fields and experiments per construct - and the fraction of cells displaying AAJs. Brief tallies may also clarify cases where a few AAJs appear positive (e.g., FCHSD2, ACAP1) or where patterns vary (e.g., BAIAP2 on outward vs inward AAJs), reducing the chance that technical negatives are read as biological ones.
• Overexpression/EGFP aggregation. Transparency around occasional EGFP-related aggregation (e.g., SNX8, Endophilin-B1) could be beneficial(1). Noting when aggregates were observed - and adding an "overall localization phenotype" plus an "OE aggregate observed: yes/no" column to Table 1 - would make strengths and limits of the screen easier to weigh.
• Subset verified by IF (Fig. 2c). A brief note on why this subset was validated (e.g., antibody performance/availability) could prevent over-interpretation. Since some non-IF-validated proteins reappear later, a short pointer may help readers not to infer, for example, that FER has been validated to lack endogenous or junctional signal.

Minor

• Fig. 2a/b. A small cartoon summarizing the three phenotypes (overlapping, non-overlapping, trailing) could orient readers quickly.
• Image mark-up. Color-coded arrows on composites and grayscale panels - similar to Figs 4-5 - would likely make BAR-junction co-localization easier to follow.
• Supp. Fig. 2. Clustering proteins by domain class (as in Supp. Fig. 3) may make the survey more readable.
• Supp. Fig. 3 (expression filter). Because cell atlas averages can mask subtype patterns (e.g., FCHO1 is present in various EC subtypes), a brief cross-check against a few EC datasets (HUVEC, HAEC, HAOEC, HBMEC and/or venous/arterial ECs) could illustrate heterogeneity or, conversely, support broader generality. This can be useful to even expand in discussion; how differential expression of these proteins can result in differential junctional dynamics in veins vs. arteries.
• Supp. Fig. 4a (WB layout). Standardizing crop sizes and briefly noting housekeeping choices or intended cross-gene effects that were aimed to be demonstrated(ARHGAP17) could improve legibility and interpretation.

BIN1 and SNX9 control endothelial front-rear polarity and collective migration
(Fig. 3; Suppl. Fig. 4)

Major

• Wound-healing assays and cell density. In several images/videos the overall cell density appears different between control and shRNA groups - most noticeably with shBIN1. A short description of seeding density, confluence at start, and any differences in observed proliferation or viability would be valuable context.
• Polarity readout versus junction state. The statement that unchanged Golgi polarity implies migration independent of junction-driven behavior may read a bit strongly given the Suppl. Fig. 4 examples (e.g., thinner junctions with shBIN3, reduced cytosolic VE-cadherin with shEndophilin-A2 in the shown ROI). Complementing the polarity metric with a basic junction intensity/morphometric readout - or adding a line that some candidates may still influence AAJs despite a negative polarity endpoint - could reduce the chance that readers interpret these as negatives.

Spatiotemporal recruitment of PACSIN2, BIN1 and SNX9 during junction remodelling
(Fig. 4)

• The image analysis is excellent and clearly lays out time-resolved recruitment to AAJs.

Minor

• Because PACSIN2's sub-junctional pattern mirrors the curvature landscape in Fig. 1, a correlative cryo-SIM/FIB-SEM snapshot for BIN1 and SNX9 (even a single well-chosen example per protein) could nicely reinforce spatial separation narrative. If additional cryo-data aren't feasible, a brief note on how the ROIs in Fig. 4 map onto the curvature bins in Fig. 1 would also help readers connect the two datasets.

SNX9 associates with dynamin-2 during asymmetric junction remodeling
(Fig. 5)

Major

• Mechanistic link to DNM2 . The independence from PACSIN/EHD4/MICAL-L1 is well shown, and the SNX9-DNM2 co-localization is convincing. To close the loop on mechanism, silencing DNM2 alongside SNX9 to observe whether the observed junctional, polarity, and migration defects are further exacerbated, would make the conclusion land more firmly.

Minor

• Cell type context. BOECs and HUVECs appear interchangeably across experiments. A short rationale (e.g., availability, expression profile, or imaging suitability) and a line confirming that the key trends are consistent across the two would reduce perceived variability when comparing Fig. 4 and Fig. 5.

SNX9 controls collective migration in the zebrafish CCV
(Fig. 7)

Major

• Staging and controls. It would be helpful to know exactly when morphology was assessed in uninjected vs crispants, and whether any subtle developmental delay was observed. Given that the CCV phenotype is relatively modest, a brief control gRNA cohort and/or a snx9a/b rescue (even on a smaller subset) would be reassuring. If these additions aren't practical, a clear note on limitations and staging checks would still strengthen interpretation.
Minor
• In vitro alignment. The endpoint data from the scratch assays (Supp. 4c) could be lightly re-analyzed with the same junction straightness/bifurcation descriptors used in the live AAJ analyses. Even a small concordance plot (e.g., straightness vs condition, focusing on shSNX9) would help tie the in vitro and in vivo readouts together.

Discussion

• Endothelial heterogeneity & curvature context. While the manuscript nicely emphasizes membrane composition and curvature preferences, a brief nod to heterogeneous expression across EC subtypes (e.g., arterial vs venous beds) and the possibility that curvature dynamics differ by vascular context could help frame generalizability. Even if outside the present scope, flagging this as a promising follow-up (e.g., profiling in defined arterial/venous settings or subtype-matched lines) would guide readers on where this could go next.
• Redundancy and genetic compensation. The relatively modest or absent phenotypes for some candidates may reflect functional overlap. It could be helpful to underline this as a limitation and point to combinatorial perturbations or inducible degradation as a natural next step to map the machinery more completely. Framing it this way keeps the current results intact while signaling realistic routes to sharpen mechanism (e.g., pairwise KOs or acute degron tools to reduce compensation)

Methods

• For qPCR, a few extra lines could make replication easier: qPCR chemistry (dye vs probe), primer sequences/efficiencies and melt-curve checks, reference gene choice and ΔΔCt details (including how shControl baselines were handled), n values and outlier handling

Minor comments

1. Figure formatting. Slight alignment/size tweaks across subpanels could make the multi-panel figures read more cleanly.
2. Nomenclature. Unifying Endophilin-A2 vs SH3GL1 across text and figures would avoid confusion.
3. Live imaging videos. Simple arrow/ROI labels (mirroring the figures) could guide viewers to the key events without rewatching.

References
1. Cranfill, P. J. et al. Quantitative Assessment of Fluorescent Proteins. Nat Methods 13, 557-562 (2016).

Point to point rebuttal to editors and reviewers for JCB manuscript #202509207

Dear Dr. Huveneers,

Thank you for submitting your manuscript entitled "Nanoscale junctional membrane curvatures recruit BIN1 and SNX9 for endothelial collective migration". The manuscript was assessed by expert reviewers, whose comments are appended to this letter. We invite you to submit a revision if you can address the reviewers' key concerns, as outlined here.

You will see that all reviewers are generally supportive, thinking your paper provides significant advances towards the role of specific BAR proteins in asymmetric adherens junctions morphology. They all provide constructive feedback, and we think your manuscript could improve if you were able to address their comments. This includes testing whether the effects of SNX9 and dynamin-2 are dependent (e.g. by using double knockdowns), as recommended by reviewers #1 and #3. All other concerns voiced by the referees should further be addressed.

We included new experiments using dynamin-2 knockdowns to test whether the effect of SNX9 on junction turnover and collective migration is mediated through its recruitment. The knockdown of dynamin-2 perturbed junction turnover, resulting in increased AAJ length and prolonged lifetimes (**Fig. 6j-l and Video 12**). Notably, shDNM2-1 ECs failed to orient the Golgi toward the scratch wound (**Fig. 6m; Supplementary Fig. 4f**), however, these cells still migrated randomly and closed the wounds within a comparable timeframe to shControls (**Fig. 6n; Supplementary Fig. 4g and Video 13**). Combined shSNX9 and shDNM2 knockdown did not further exacerbate the migration defect relative to shSNX9 alone (**Fig. 6n; Supplementary Fig. 4g and Video 13**). Together, these findings show that SNX9 regulates VE-cadherin turnover at AAJs, an effect that may be partially mediated through the recruitment of dynamin-2. Please see textual changes in the corresponding results section and Figure 6.

While you are revising your manuscript, please also attend to the following editorial points to help expedite the publication of your manuscript. Please direct any editorial questions to the journal office.

GENERAL GUIDELINES:

Text limits: Character count for an Article is < 40,000, not including spaces. Count includes title page, abstract, introduction, results, discussion, and acknowledgments. Count does not include materials and methods, figure legends, references, tables, or supplemental legends.

IMPORTANT: It is JCB policy that if requested, original data images must be made available. Failure to provide original images upon request will result in unavoidable delays in publication. Please ensure that you have access to all original microscopy and blot data images before submitting your revision.

Supplemental information: There are strict limits on the allowable amount of supplemental data.

Articles may have up to 5 supplemental figures. Up to 10 supplemental videos or flash animations are allowed. A summary of all supplemental material should appear at the end of the Materials and methods section.

We have adapted the supplemental files accordingly by combining multiple video's and by omitting the CCV development example movie of control zebrafish. Based on reviewers questions we performed new experiments of junction remodeling in mosaic monolayers and with dynamin-2 knockdown cells and thus added thes as a new videos. The total number is now 15 video's, which was approved by personal communication with the handling editor. Supplemental figures were combined and are now 5 in total.

Please note that JCB now requires authors to submit Source Data used to generate figures containing gels and Western blots with all revised manuscripts. This Source Data consists of fully uncropped and unprocessed images for each gel/blot displayed in the main and supplemental figures. Please be sure to provide one Source Data file for each figure gels, blots, and/or capillary electrophoresis assays along with your revised manuscript files. File names for Source Data figures should be alphanumeric without any spaces or special characters (i.e., SourceDataF#, where F# refers to the associated main figure number or SourceDataFS# for those associated with Supplementary figures). For traditional gels and blots, the lanes of the gels/blots should be labeled as they are in the associated figure, the place where cropping was applied should be marked (with a box), and molecular weight/size standards should be labeled wherever possible. Source Data Figures should be provided as individual PDF files (one file per figure). Authors should endeavor to retain a minimum resolution of 300 dpi or pixels per inch. Please review our instructions for export from Photoshop, Illustrator, and PowerPoint here: https://rupress.org/jcb/pages/submission-guidelines#revised

We have included corresponding Source data files for Figure 6 and Supplemental Figure 4.

When submitting the revision, please include a cover letter addressing the reviewers' comments point by point. Please also highlight all changes in the text of the manuscript.

All changes are highlighted in the track changes Word version of the revised manuscript.

We hope that the comments below will prove constructive as your work progresses. We would be happy to discuss them further once you've had a chance to consider the points raised in this letter.

Thank you for this interesting contribution to Journal of Cell Biology. You can contact us at the journal office with any questions at cellbio@rockefeller.edu.

Sincerely,

Sandrine Etienne-Manneville
Monitoring Editor
Journal of Cell Biology

Gabriele Stephan
Scientific Editor
Journal of Cell Biology

Reviewer #1:

The authors evaluated asymmetrical adherens junctions (AAJs) between leader and follower cells and their modulation using complex and state-of-the-art techniques including FIB-SEM and high resolution live cell imaging, which enable detailed insights into the ultrastructure and dynamics of these junctions. Further, the authors identify specific BAR proteins as novel regulators of front-to-rear polarity and AAJs in collective cell migration of endothelial cells. The study is interesting and novel by adding insights into the differential contributions of BAR proteins for regulating membrane curvature in AAJs, which profoundly contribute to the process of collective cell migration. Clear strengths of the study are the provision of AAJ 3D reconstructions and the application of different cell culture approaches together with the inclusion of a zebrafish model. However, some weaker aspects remain which should be improved as indicated below.

Major points:

1. In their screen, the authors identified several AAJ-associated bar proteins, selected some of these and tested them further in loss of function studies, demonstrating contributions to collective cell migration. However, it is unclear whether these changes are a result of the BAR proteins localized at the AAJ, as the authors argue, or whether proteins in different localizations are relevant, too. One way to test this would be to select one of the proteins present in endothelial cell but not localized to AAJs and evaluate the functional outcome by silencing approaches. This may serve as excellent negative control, too.

We thank the reviewer for this important point. Indeed, our current model is that SNX9 and the other junctional BAR proteins we describe contribute to collective cell migration specifically because they are recruited to asymmetric adherens junctions that form during this process. We agree that formally distinguishing between AAJ-localized and non-junctional roles of BAR proteins would further strengthen the study. Our data support a localization-specific role based on the strong enrichment of the investigated BAR proteins at AAJs (Fig. 2), the correlation between junctional localization of PACSIN2, SNX9 and BIN1 with local junction remodeling stages (Fig. 4), and the functional effects of their depletion on collective migration (Fig. 2) and junction dynamics (Fig. 6). At the same time, we would not dare to make the claim that non-junctional BAR proteins do not contribute to migration-related phenotypes, as this remains an open question.

Importantly, our dataset already includes elements that partially address this concern from the reviewer. As shown in Fig. 3a, knockdown of the BAR proteins PICK1 and ASAP1, both of which localize to junctions, did not result in detectable defects in endothelial collective migration. On the other hand, the depletion of SH3GL1/Endophilin-A2 and BIN3 decreased wound closure capacity, however no significant differences were detected in front-rear polarity, suggesting that signaling between leader and follower cells was unaffected in these cells. Together, these findings suggest that not all junction-associated BAR proteins contribute equally to this process and can therefore serve as internal controls supporting the specificity of our observations regarding BIN1 and SNX9.

We also acknowledge the reviewer’s point that additional subcellular pools of junctional BAR proteins may play important roles. Experimentally disentangling these spatial contributions is, however, challenging. For example, mutating the BAR domain of proteins such as SNX9 or BIN1 would disrupt

their membrane-binding and curvature-sensing properties globally, thereby affecting their functions across multiple cellular compartments rather than specifically at AAJs. Such approaches would therefore not allow us to directly address the localization-specific contribution.

We have now clarified these points and significantly expanded the discussion in the revised manuscript (see track changes in Discussion section), where we explicitly acknowledge the limitations and highlight important direction for future investigation. In addition, we have expanded the overall BAR-protein localization dataset by including the localization characterization with additional subcellular compartments that were observed: junctional, punctate or vesicular, tubular, cytoplasmic, cortical or other observed localizations to point readers toward non-junctional localization of BAR proteins in ECs. (see revised Table 1).

2. Figure 2: How did the authors decide what BARs associate with AAJs? The morphology and size of the cells appears somewhat different in the images provided. Please comment on potential differential states of cells influencing AAJ morphology and BAR association. How did the authors ensure similar states (in terms of density, proliferative stage, quiescence, dividing phase, migratory phase)?

We thank the reviewer for this comment. The assessment of cellular localization of GFP-tagged BAR proteins is now performed 3 independent times for each BAR protein using a standardized transfection protocol. The next day transfected cells were seeded to glass coverslips at defined densities to obtain reproducibly subconfluent endothelial monolayers at the time of fixation and confocal imaging. Imaging was consistently performed within a fixed time window after transfection to minimize variability in expression levels and cellular state.

To reduce heterogeneity in cell behavior, only cells within the monolayer were analyzed, while isolated cells and dividing cells were excluded. This ensured that the majority of analyzed cells were in a comparable, junction-forming state. While some variability in cell morphology and size is inherent to endothelial cultures, these controlled conditions minimized differences related to cell density, proliferative status, or migratory behavior.

Regarding BAR-AAJ association, we observed that the localization patterns of BAR proteins varied substantially, ranging from clear overlap with VE-cadherin at adherens junctions to partial or no overlap, as well as differences in the fraction of AAJs labeled within a given cell. Due to this heterogeneity and the lack of a uniform signal pattern across conditions, automated image analysis approaches were not sufficiently effective.

Instead, BAR association with AAJs was assessed by manual inspection, based on the overlay of GFP (BAR proteins) and VE-cadherin signals. Importantly, this analysis was performed independently by at least three experienced researchers using consistent criteria for defining junctional association: Only signals that were clearly co-localized with or directly adjacent to VE-cadherin-positive junctions were scored as associated. The consistency of observations across independent evaluators supports the robustness of this approach. For SNX9, BIN1 and PACSIN2 the observed localization patterns were further confirmed in live imaging experiments.

We have now added these clarifications for the readers in the revised Methods, please see textual section under the “Screen localization of BAR-GFP proteins in endothelial cells” heading.

3. Does the AAJ/BAR protein association (at least of those BARs the authors later focus on) also occur under the clear collective cell migration conditions in Figure 3?

In Figure 4a-k we have studied the spatiotemporal recruitment of PACSIN2, SNX9 and BIN1 in collectively migration endothelial cells of scratched endothelial monolayers (as indicated by "*live imaging of migrating BOECs" in the text*). We apologize for not sufficiently explaining this, we have now adapted the corresponding results text, methods and figure legends to emphasize this more clearly.

4. The temporal relationship of BIN1, SNX9 and PACSIN in Figure 4 is hard to follow based on the images provided in j and k (disclaimer: my Macbook refused to play the .avi videos). How many AAJs/cells were imaged? Please provide an analysis.

We thank the reviewer for highlighting this point and for noting the issue with the .avi format. We have now converted all videos to .mp4 format in accordance with journal guidelines to ensure compatibility across platforms. In Video 8, at least five AAJs can be tracked, each displaying sequential recruitment of PACSIN2 followed by SNX9. Across our dataset, we consistently observed this temporal pattern qualitatively. To further support this observation, we now performed a quantitative temporal analysis by measuring fluorescence intensity over time at individual AAJs (n = 10 AAJs per condition, from at least 3 independent live imaging). This analysis revealed that SNX9 recruitment follows PACSIN2 with a delay of approximately 170 seconds, whereas BIN1 and PACSIN2 are often recruited simultaneously. This quantitative analysis confirms our initial qualitative observations and has been added to the revised manuscript as Fig. 4l. To improve clarity, we have also revised the corresponding figures and videos by adding coloured arrows emphasizing one AAJ involving representative recruitment of PACSIN2 and BIN1/SNX9.

5. Similarly, the claim that "dynamin-2 is recruited to AAJs precisely at the moment of junctional scission and internalization" is not comprehensively shown in the image sequence of Fig 5J. Please provide an analysis and a better visualization.

We thank the reviewer for this comment and apologize for the lack of clarity in the original visualization. We have revised the corresponding figure and video by simplifying the indications with coloured arrows pointing at VE-cadherin internalization events that co-occurr with dynamin-2 recruitment. In addition, we have improved the indicators in Fig. 5k to better illustrate the temporal relationship between dynamin-2 and the investigated BAR proteins. To better reflect the temporal resolution of our data, we have toned down our statement in the Results section to: "Live-cell imaging of ECs expressing dynamin2-GFP further revealed that dynamin-2 is recruited to AAJs during junctional scission and internalization".

6. Figure 6: Since SNX9 and dynamin-2 are co-recruited and overlap at the AAJ, can the authors show whether the effects of shSNX9 could be rescued by Dynamin-2? Further, is dynamin-2 also required for AAJ stability?

We thank the reviewer for these important questions and have further investigated the functional relationship between SNX9 and dynamin-2. The more efficient shRNA (shDNM2-2) resulted in loss of EC adhesion, showing its importance, but precluding functional analysis. The moderate depletion achieved by shDNM2-1 was sufficient to perturb junction turnover, resulting in increased AAJ length and prolonged lifetimes (Fig. 6j-l and Video 12). Next, we assessed the role of dynamin-2 in endothelial collective migration. Notably, shDNM2-1 ECs failed to orient the Golgi toward the scratch wound (Fig. 6m; Supplementary Fig. 4f), however, these cells still migrated randomly and closed the wounds within

a comparable timeframe to shControls (Fig. 6n; Supplementary Fig. 4g and Video 13). Combined shSNX9 and shDNM2 knockdown did not further exacerbate the migration defect relative to shSNX9 alone (Fig. 6n; Supplementary Fig. 4g and Video 13). Together, these findings show that SNX9 regulates VE-cadherin turnover at AAJs, an effect that may be partially mediated through the recruitment of dynamin-2. The manuscript has been revised to include these new insights.

7. Do AAJs also occur in the zebrafish model? Does SNX9 decorate AAJs in the migrating cardinal vein ECs? It would be important to show the relevance of the authors' findings in vivo.

We thank the reviewer for raising this point. During collective migration of endothelial cells in the CCV, junctions are formed and dynamically remodeled in a manner reminiscent of AAJs observed in cultured endothelial cells (see new Supplementary Fig. 5a and see below). Unfortunately, suitable antibodies for reliable immunofluorescence detection of SNX9 in zebrafish are currently not available, precluding direct assessment of its subcellular localization in this model.

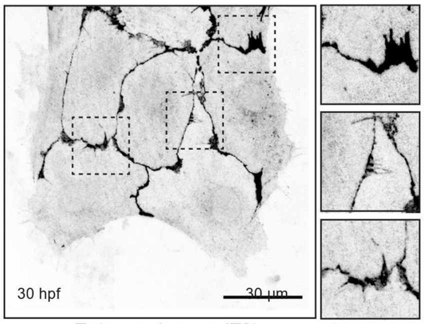

However, time-lapse imaging revealed that snx9ab crispants still form AAJ-like structures, although endothelial cell–cell junctions in migrating CCV endothelial cells appeared noticeably less straight compared to those in uninjected siblings. These observations support a role for SNX9 in regulating endothelial junction organization during collective migration in vascular development. At the same time, our findings suggest that there may be differences in junctional dynamics between endothelial cells *in vitro* and *in vivo*, which we now mention in the concluding paragraph of the Discussion section of the revised manuscript.

Minor points:

1. The authors use different loading controls for the western blot experiment in Supp figure 4a. Specifically, ARHGAP17 and VE-cadherin are not valid controls since they are not housekeeping proteins, and their expression might be modulated by the knockdown of BAR proteins shown.

We thank the reviewer for raising this point. Upon revisiting the original data, we realized that ARHGAP17 was incorrectly labeled in the Illustrator file of Supplementary Fig. 4a; the blot was in fact probed with an anti-vinculin antibody (which runs at ~124 kDa), which serves as an appropriate loading control. This labeling error has now been corrected in the revised figure and legend, and the original uncropped blots are provided in the Source Data file. We apologize for the confusion caused.

We agree with the reviewer that VE-cadherin is not an ideal loading control, as its expression may be influenced under these experimental conditions. The shRNA screen was performed in primary

endothelial cells and required substantial cell numbers for the scratch wound migration assays. Consequently, the available material per condition was limited, and it was not feasible to collect sufficient lysate for repeated Western blot validation or re-probing with additional housekeeping controls.

Importantly, the primary purpose of these Western blots is to verify that the shRNAs used in the puromycin-selected, transduced cells achieved knockdown of their target proteins in the same cell populations used for the scratch assay screen of Figure 3. These blots therefore serve as a qualitative validation of knockdown efficiency rather than as quantitative measurements, and were performed once per condition due to material constraints. We chose to include them in the manuscript to support the validity of the knockdown screen. Importantly, follow-up functional experiments on the top candidate, SNX9 and dynamin-2, were independently repeated at least three times and quantitatively analyzed, confirming the robustness of the observed knockdown phenotypes (Figure 6).

2. Supp figure 2: Please provide high magnification images for the junctions to the images provided. How many cells were evaluated to decide if a BAR protein localizes to an AAJ?

We appreciate the reviewer's request for higher-magnification views of the junctional regions. To provide a comprehensive overview of BAR protein localization across the cell, we intentionally prioritized lower-magnification images in Supplementary Fig. 2, allowing visualization of the overall subcellular distribution within the endothelial monolayer. Due to space constraints, we were unable to include high-magnification ROIs for all GFP-tagged BAR proteins in the supplementary figures. Instead, we uploaded a high resolution version of Supplemental Figure 2 to allow readers to zoom in on subcellular details. Of note, we have included additional representative high-magnification images of all the junctional localized BAR-protein in Figure 2b. To assess whether a given BAR protein localizes to the AAJ, we analyzed three independent transfections and examined at least 12 transfected cells per construct that displayed clear expression. This information has now been clarified in the Methods section of the revised manuscript.

3. Please provide exemplary images for the begin and end time points of the results in Figure 3b in the supplements.

We have now included images of t=0 and t=7 hours post scratch as Supplemental Fig 4b.

4. The legend for PACSIN2 in Figure 4f is not clearly visible with the yellow color. Please adapt.

We have now adapted the legend to enhance visibility.

5. Please provide individual data points for Supplementary Figure 4b and statistical analysis. Are the transcripts normalized to an internal control like GAPDH or actin?

We thank the reviewers for this important point regarding qPCR reporting and reproducibility. The qPCR data in question were derived from samples collected during the scratch wound assays presented in Figure 3. Due to the use of primary HUVECs following lentiviral transduction, the available cellular material for downstream analyses was limited during these experiments. As a result, qPCR measurements could only be performed for a single independent experiment, which does not allow for meaningful statistical analysis. To avoid potential overinterpretation, we have removed the corresponding qPCR panel from the supplemental data. Instead, knockdown efficiency for the shRNA constructs used in the scratch migration assays was independently validated at the protein level. As

we answered earlier at minor point 1 from the reviewer, these blots serve as a qualitative validation of knockdown efficiency.

6. The color legends for the figure in Figure S4c is missing.

Legend is now included.

7. The y-axis is incomplete for Figure S5a.

Axis label has been corrected.

Reviewer #2:

This manuscript by Janssen et al describes to nanoscale architecure of the asymmetric adherens junctions which are membrane invaginations formed between leader and follower migrating endothelial cells. The localization and distribution of a large number of BAR domain proteins was screened, identifying several of interest. Of these SNX9 and BIN1 were investigated in detail for their distribution and dynamics leading to the discovery that these proteins are required for collective endothelial cell migration. The imaging is excellent and I found the manuscript enjoyable to read. The in vivo experiment is a convincing demonstration of the role of SNX9. My detailed comments are below.

1. Overall, the largest question I have is about which cell, leader or follower, is recruiting the BAR proteins. Given the diversity of membrane curvature, there is positive and negative curvature in the AAJ membrane from each cell. The authors should discuss this concept and provide their thoughts.

We thank the reviewer for raising this important point. At adherens junctions, the double-membrane structure can be perceived intracellularly as either convex or concave, depending on the cellular perspective. In principle, this suggests that BAR proteins could be recruited from either side of the junction, depending on their curvature-sensing properties. Importantly, in a collectively migrating monolayer, leader and follower identities are not fixed but are defined locally by the direction of cell movement. Thus, individual cells can simultaneously exhibit both leader and follower regions depending on their position and dynamics within the tissue.

Secondly, our FIB-SEM analysis revealed both concave and convex curvatures across the junction, and we found BAIAP2L2 a negative curvature sensor to also localize at follower-side AAJs. This may indicate that local negative curvatures still recruit negative curvature sensors, however, we did not find any of the other negative curvature sensors (I-BARs) with strong follower-cell AAJ association hinting at a preference for convex BAR proteins.

We have previously addressed this question for PACSIN2, demonstrating that this F-BAR protein is recruited from one side, the followers', at the cell-cell contact interface (Dorland et al, 2016; PMID: 27417273). In line with this, our live-cell imaging analyses (e.g., SNX9, BIN1, Video 5) suggest that BAR protein recruitment events at AAJs occur on the side of the junction corresponding to the leading edge of the follower cell. These observations show that recruitment is not determined by intrinsic follower or leader cell identity, but rather by the local geometry and dynamics of the junctional landscape.

To confirm the cellular origin of SNX9 recruitment for the revisions, we performed live-cell imaging in mosaic endothelial monolayers, in which half the cell population expressed lentivirally transduced GFP-SNX9 and the other half mScarlet-SNX9. These experiments showed that the presence of SNX9 at AAJs is either GFP or mScarlet fluorescent (Fig. 4m and Video 9), indicating that the junctional recruitment of SNX9 occurs from one side. Consistently, SNX9 recruitment was observed at the leading edge of the follower cell, i.e., the side of the junction facing the direction of movement. This is now discussed in the corresponding results section of Figure 4m.

2. An experiment to test the contribution of BAR domain proteins in leader vs follower cells would add to the mechanism, but may be beyond the scope and is not required. One could imagine mixing BAR domain expressing and knockdown cells or cells expressing BAR-GFP or BAP-mScarlet and then examining the AAJs between them. This would allow distinction of recruitment and localization from the leader or follower cell.

We thank the reviewer for this insightful suggestion. As mentioned at point 1, to address this question we live imaged mosaic endothelial monolayers, in which half the cell population expressed lentivirally transduced GFP-SNX9 and the other half mScarlet-SNX9. These experiments showed that the presence of SNX9 at AAJs is either GFP or mScarlet fluorescent (**Fig. 4m and Video 9**), indicating that the junctional recruitment of SNX9 occurs from one side, that of the follower cell, to the junctional interface.

3. This comment is related to 1. The screen identified many different BAR domain proteins (F-BAR, I-BAR, etc.). Each is known to bind or shape membranes in different ways. Given the different membrane curvatures quantified, can the authors comment on where/which locations the proteins of interest are most likely recruited to?

We thank the reviewer for this important point. AAJs display complex membrane geometries (Fig. 1), including regions of both convex and concave curvature. This heterogeneous curvature landscape provides a structural framework that could accommodate the recruitment of different BAR domain proteins according to their known curvature preferences.

Based on this, we propose that BAR proteins with distinct curvature-sensing properties may be recruited to specific sub-junctional regions within AAJs. For example, proteins with preference for convex curvature may localize to outwardly curved membrane regions, whereas those sensing concave curvature may associate with inwardly curved domains. We attempted to map this out for PACSIN2 with the cryo-SIM approach. However, the current technical limitation is the resolution of the SIM in lateral and axial dimensions in combination with the FIB-SEM to map the specific subjunctional regions with corresponding BAR proteins, for which no better techniques exist yet.

Secondly, our screen indicates that the intrinsic properties of BAR domains alone are not sufficient to ensure their recruitment to AAJs, as not all BAR proteins of the same family are localizad to junctions in similary patterns, suggesting that additional factors such as protein-protein interactions or local cellular context contribute to their localization.

We agree that determining the exact side and sub-junctional membrane geometry recognized by each BAR protein would be highly informative. However, systematically addressing this would require extensive experimental efforts, which are beyond the scope of the current revision. We have now expanded the Discussion section accordingly by adding this paragraph:

"However, curvature preference alone is insufficient to explain how the various BAR proteins are recruited to AAJs. The localization screen revealed that not all members of a given BAR subfamily localize to junctions equivalently. This indicates that other protein-protein interactions through other domains and additional cellular determinants beyond membrane geometry govern their recruitment. Resolving which curvature subdomains each BAR protein occupies within AAJs, and what auxiliary factors license their recruitment, represent an intriguing direction for future studies."

4. In Figure 1D,E I found the analysis of curvature confusing. For example, some highly curved membranes like the small circles in the bottom of AAJ3 are indicated to have a very low curvature where by eye they seem to have high convex curvature. It may be informative to divide this analysis into convex and concave (or positive and negative) curvature. Currently I interpret the image as showing all convex curvatures denoted as extremely large or tending toward infinity. The authors could also clarify which "side" the curvature is being interpreted from (ie from the trailing cell).

We thank the reviewer for this helpful comment and for pointing out the potential source of confusion. The curvature analysis shown in Fig. 1 is interpreted from the follower cell's side and we have now clarified this explicitly in the Results section and figure legends. Indeed, very little of the surfaces of the junctional membrane showed a mean negative curvature. That may sound a little counter-intuitive, but the reasoning is that while the junctions have areas that curve "in" somewhat in one direction, they are still highly positively curved in the orthogonal direction. We've attached a sketch drawing to help illustrate this in 3D for the reviewer. This causes that the local Gaussian curvature is mainly dominated by the positive component. Please note that the images in Fig. 1e also show junctional regions with yellow and white signals showing the existence of narrow convex curvatures.

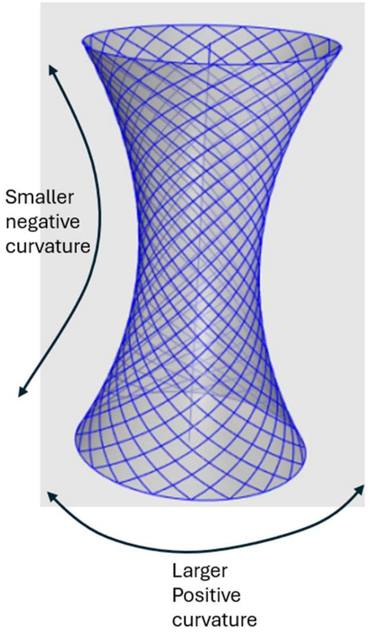

5. In AAJ1and AAJ2 in Fig 1 it appears there are vesicles inside the finger structure. Could these be endocytosed from the leader cell? It seems that as endocytosis happens in the follower cell, a process to balance the membrane area should be happening in the leader cell.

This is a very interesting observation. Previous pulse labelling experiments for VE-cadherin have shown that there is about 9% trans-endocytosis of VE-cadherin from AAJs between leader and follower cells (Dorland et al 2016), indicating that membrane exchange between adjacent cells can occur at these sites. While we can only speculate, it is possible that membrane internalization on the follower cell side is accompanied by compensatory membrane trafficking on the leader cell side to maintain junctional balance. In support of this, we indeed found single membrane structures budding from the junctional interface toward both the leader and follower cell sides (Supplementary Fig. 1b).

Together, these observations are consistent with the possibility that membrane turnover at AAJs involves bidirectional trafficking, which may contribute to maintaining membrane balance during junction remodeling. However, further experiments would be required to directly test the origin and fate of these vesicular structures.

6. Can the authors please note how many junctions were examined by FIBSEM? Is there a population based analysis, perhaps of curvature and VE-Cad or PASCIN2 distribution possible?

We thank the reviewer for this question, which aligns well with the original ambition of the experiment. In the manuscript, we present curvature analyses of representative individual PACSIN2-positive adherens junctions (AAJ1–3), which are shown in Fig. 1 for SIM overlays and 3D renderings. In total, five junctions were examined by FIB-SEM. These included three PACSIN2-positive junctions (AAJ1–3), one junction with low PACSIN2 staining (AAJ4), and one junction without detectable PACSIN2 recruitment (AAJ8).

Using this dataset, we explored whether membrane curvature correlates with PACSIN2 recruitment, i.e., whether specific curvature features are enriched in PACSIN2-positive junctions. However, analysis of the overall curvature distributions did not reveal clear differences between PACSIN2-positive and negative junctions. These preliminary data are provided as information for the reviewer below.

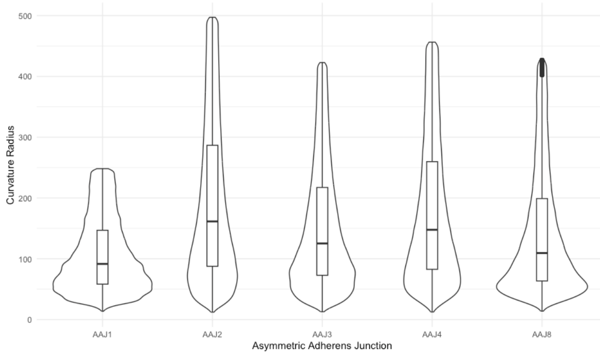

AAJ	PACSIN2	Curvature range (k)	Most frequent value k
1	+	0.011 – 0.27	0.046
2	+	0.0111-0.327	0.03
3	+	0.0088 – 0.27	0.041
4	+ / -	0.0083 - 0.3068	0.02913264
8	-	0.0092 - 0.286	0.044

We further attempted to perform a population-based analysis by registering the detailed curvature maps derived from FIB-SEM at 4x4x4 nm onto the corresponding SIM images to correlate curvature with subjunctional PACSIN2 intensity. However, the SIM data proved to be of insufficient spatial resolution with lateral x,y resolution ~100-130 nm, and z ~100-250 nm to reliably correlate specific curvature values with PACSIN2 intensity on a per-pixel basis. The resolution of the SIM (100 lateral, 100-250 axial) is insufficient to recognize subtle differences in the narrow curvature ranges present at AAJs (30-150 nm). This limitation we encountered stresses the need for further technical developments that combine different super-resolution fluorescence microscopy methods, such as single molecule localization microscopy, with electron microscopy. We have now explicitly noted this technical limitation of our study in the Discussion.

Minor Points

Very nice screen of BAR domain proteins. Could the authors describe if there a specific metric used to define if a BAR domain protein was associated and indicate "n" for these screens.

The assessment of cellular localization of GFP-tagged BAR proteins was now performed 3 independent transfections for each BAR protein in the revised manuscript. Regarding BAR-AAJ association, we observed that the localization patterns of BAR proteins varied substantially, ranging from clear overlap with VE-cadherin at adherens junctions to partial or no overlap, as well as differences in the fraction

of AAJs labeled within a given cell. Due to this heterogeneity and the lack of a uniform signal pattern across conditions, automated image analysis approaches were not sufficiently effective.

Instead, BAR association with AAJs was assessed by manual inspection, based on the overlay of GFP (BAR proteins) and VE-cadherin signals. Importantly, this analysis was performed independently by at least three experienced researchers using consistent criteria for defining junctional association. Only signals that were clearly co-localized with or directly adjacent to VE-cadherin-positive junctions were scored as associated. We have now clarified these points for the readers by adding a "Screen localization of BAR-GFP proteins in endothelial cell" paragraph in the revised Methods section.

In Fig. 4 The wording around the BAR domain protein distribution in AAJs was somewhat confusing. The definitions of "mid", "over" and "trailing end" are clear in the text and in Fig 4B linescans. The term "subjunctional region" evoked thinking the classes referred to specific locations along the AAJ. Consider "subjunctional distribution" or something similar? Action not required.

We thank the reviewer for this remark. To clarify this for readers we now refer to subjunctional distribution instead.

Reviewer #3:

In this manuscript, Janssen and colleagues set to expand our understanding on the formation and dynamics of VE-cadherin-based asymmetric adherens junctions (AAJs) in endothelial cells. Using state-of-the-art correlative cryo-SIM/FIB-SEM, they provide a volumetric view of AAJs and reveal pronounced curvature heterogeneity. Building on this, the authors utilize a screen of BAR-domain proteins and identify several AAJ-associated factors with functional impact on collective migration. Among these, BIN1 and SNX9 are developed further, with spatiotemporally distinct localization and evidence that SNX9 contributes to junctional turnover in vitro and collective migration in vivo. Together, the study extends our understanding of polarized endothelial junction behaviour and points to a sequential machinery that sculpts highly curved and functional structures, with potential relevance beyond endothelial biology. It is also a technical tour de force. That said, several parts need clearer explanation or occasionally additional support for some of the main claims.

- Most notably, the screen and how BAR-protein 'hits' and 'non-hits' were filtered should be spelled out so readers don't mistake technical negatives for biological ones.
- The link between the impressive volumetric imaging and the later assignment of new AAJ proteins could also be tightened by re-use of established methodology.
- Finally, a few narrative jumps (e.g., choosing GOI-rich junctions, possible overexpression bias) make some results read stronger than they're currently controlled for.

With them addressed, the study would make a solid and broadly useful contribution. Below I list major and minor points by chapter/figure, followed by notes on discussion and methods.

Multiple BAR proteins are recruited to asymmetric adherens junctions (Fig. 2a-b, 2c; Table 1; Supp. Figs 2-4)

Major

• Screen sampling and basic tallies. It could help readers if the library screen included simple counts - how many cells/fields and experiments per construct - and the fraction of cells displaying AAJs. Brief

tallies may also clarify cases where a few AAJs appear positive (e.g., FCHSD2, ACAP1) or where patterns vary (e.g., BAIAP2 on outward vs inward AAJs), reducing the chance that technical negatives are read as biological ones.

We thank the reviewer for the comments to improve the BAR protein screen. The assessment of cellular localization of GFP-tagged BAR proteins was performed using a standardized Lipofectamine transfection protocol. The following day, transfected cells were seeded onto fibronectin coated glass coverslips at defined densities to obtain reproducibly subconfluent endothelial monolayers at the time of fixation and confocal imaging. Imaging was consistently performed within 48 hours after transfection to minimize variability in expression levels and cellular state. Only cells within the monolayer were analyzed; isolated cells and dividing cells were excluded. BAR protein association with AAJs and other cellular compartments was assessed by manual inspection based on the overlay of GFP (BAR proteins) and VE-cadherin signals. This analysis was performed based on three independent transfections and at least 12 transfected cells per construct that displayed clear expression. Only signals that were clearly co-localized with or directly adjacent to VE-cadherin-positive junctions were scored as junction-associated. This information has now been clarified in the Methods section of the revised manuscript. Importantly, based on our live-cell imaging experience, BAR protein recruitment to AAJs is highly dynamic and transient. As a result, in fixed-cell conditions, only a subset of junctions within a given cell is expected to display detectable recruitment at any given time point. Therefore, consistent detection of junctional localization across multiple cells and independent experiments was considered indicative of bona fide association. Proteins were classified as “non-hits” when no cells with clear junctional association were detected, or when such association was observed only sporadically (e.g., in fewer than two cells in each experiment), indicating a lack of reproducible localization.

• Overexpression/EGFP aggregation. Transparency around occasional EGFP-related aggregation (e.g., SNX8, Endophilin-B1) could be beneficial(1). Noting when aggregates were observed - and adding an "overall localization phenotype" plus an "OE aggregate observed: yes/no" column to Table 1 - would make strengths and limits of the screen easier to weigh.

We have included this information to the revised Table 1 of the manuscript.

• Subset verified by IF (Fig. 2c). A brief note on why this subset was validated (e.g., antibody performance/availability) could prevent over-interpretation. Since some non-IF-validated proteins reappear later, a short pointer may help readers not to infer, for example, that FER has been validated to lack endogenous or junctional signal.

We thank the reviewer for this helpful comment. The subset of BAR proteins shown in Fig. 2c was selected based on antibody availability and performance in immunofluorescence assays. Specifically, only proteins for which antibodies produced reliable and specific signals were included for validation at the endogenous level. First, antibodies were tested in overexpression conditions to confirm their ability to detect the target protein, and subsequently, those that were validated, were subsequently used to assess endogenous localization in non-transfected cells. We have now clarified this as follows in the results section: “we investigated the bona fide localization of endogenous BAR proteins in ECs <u>using a selection</u> of commercially available validated antibodies”.

Minor

• Fig. 2a/b. A small cartoon summarizing the three phenotypes (overlapping, non-overlapping, trailing) could orient readers quickly.

We apologize for not explaining this sufficiently clear. We wanted to indicate that not each BAR protein is recruited to the junctions in a similar pattern. The mentioned (non) overlap with VE-cadherin was an example for observed differences between e.g. FER and BIN2. We have adapted the corresponding phrase textually. Since we quantified the different localization patterns of BAR proteins in Fig. 4 as mid, over and trailing end, and to help readers orient better in the AAJ ROIs, we have now generated a cartoon to explain these junctional distributions more clearly for readers. See replace figure panel 4b.

• Image mark-up. Color-coded arrows on composites and grayscale panels - similar to Figs 4-5 - would likely make BAR-junction co-localization easier to follow.

Thank your for mentionig this. To improve these visualizations, we have improved the use of coloured arrow heads to indicate junctional events in Videos 7, 8 and 10 and changed the panels across Figure 4 and 5 to follow a consistent visual style.

• Supp. Fig. 2. Clustering proteins by domain class (as in Supp. Fig. 3) may make the survey more readable.

We have followed this nice suggestion and organized the image panels according to BAR domain family member.

• Supp. Fig. 3 (expression filter). Because cell atlas averages can mask subtype patterns (e.g., FCHO1 is present in various EC subtypes), a brief cross-check against a few EC datasets (HUVEC, HAEC, HAOEC, HBMEC and/or venous/arterial ECs) could illustrate heterogeneity or, conversely, support broader generality. This can be useful to even expand in discussion; how differential expression of these proteins can result in differential junctional dynamics in veins vs. arteries.

We thank the reviewer for this valuable suggestion. We have now added an analysis of hierarchically clustered expression levels of genes encoding human BAR domain proteins across arterial, venous, capillary and lymphatic endothelial cell types in scRNA-seq data from Tabula Sapiens dataset (Tabula Sapiens Consortium et al., 2022). The results were added to the revised manuscript as **Supplementary Fig. 3b.** Log10-transformed nCPM values per gene are indicated per gene and a row-wise scaling was applied for color mapping across the cell types. Lower panel indicates gene expression of cluster markers for arterial (GJA5), venous (ACKR1), capillary (CA4) and lymphatic (LYVE1) endothelial cells. The overall analysis shows that from the junctional and endothelial expressed BAR proteins: ASAP1, FER, PICK1, Endophilin-A2 (SH3GL1), SNX9 and FBP17/FNBP1 are expressed across arterial, venous, capillary and lymphatic endothelial cell types. ARHGAP17 and BIN3 are expressed at slightly lower levels in lymphatic endothelial cells compared with other endothelial cell types, whereas BIN1 is more highly expressed in lymphatic endothelium.

We have also added to the discussion section the following statement: "In this regard, the notion that BIN1 and other BAR proteins, such as SRGAP3, are highly expressed in lymphatic ECs (Supplementary Fig. 3b), indicate that differential BAR protein expression may finely tune junctional dynamics across different vascular beds."

• Supp. Fig. 4a (WB layout). Standardizing crop sizes and briefly noting housekeeping choices or intended cross-gene effects that were aimed to be demonstrated(ARHGAP17) could improve legibility and interpretation.

We thank the reviewer for raising this point. Upon revisiting the original data, we realized that ARHGAP17 was incorrectly labeled in the Illustrator file of Supplementary Fig. 4a; the blot was in fact probed with an anti-vinculin antibody (which runs at ~124 kDa), which serves as an appropriate loading control. This labeling error has now been corrected in the revised figure and legend, and the original uncropped blots are provided in the Source Data file. We apologize for the confusion caused.

We agree with the reviewer that VE-cadherin is not an ideal loading control, as its expression may be influenced under these experimental conditions. The shRNA screen was performed in primary endothelial cells and required substantial cell numbers for the scratch wound migration assays. Consequently, the available material per condition was limited, and it was not feasible to collect sufficient lysate for repeated Western blot validation or re-probing with additional housekeeping controls.

Importantly, the primary purpose of these Western blots is to verify that the shRNAs used in the puromycin-selected, transduced cells achieved knockdown of their target proteins in the same cell populations used for the scratch assay screen of Figure 3. These blots therefore serve as a qualitative validation of knockdown efficiency rather than as quantitative measurements, and were performed once per condition due to material constraints. We chose to include them in the manuscript to support the validity of the knockdown screen. Importantly, follow-up functional experiments on the top candidate, SNX9 and dynamin-2, were independently repeated at least three times and quantitatively analyzed, confirming the robustness of the observed knockdown phenotypes (Figure 6).

BIN1 and SNX9 control endothelial front-rear polarity and collective migration (Fig. 3; Suppl. Fig. 4)

Major

• Wound-healing assays and cell density. In several images/videos the overall cell density appears different between control and shRNA groups - most noticeably with shBIN1. A short description of seeding density, confluence at start, and any differences in observed proliferation or viability would be valuable context.

For the collective cell migration screen, cells were seeded at approximately 80% confluence the day before the experiment. At the time of scratch wounding, cultures were close to 100% confluent, as intended. Proliferation rates and viability were not formally quantified following knockdown. However, based on routine monitoring, overall viability appeared normal, with the exception of a shRNA construct targeting ASAP-1 (indicated as lethal shRNA in the manuscript). Notably, in shBIN1 conditions, cultures appeared slightly less confluent from the starting point, despite plating similar numbers of cells. This suggests that BIN1 depletion may slightly affect cell proliferation or growth dynamics rather than cell viability.

Of note, in these experiments, to control for collectivity, we quantified the directionality of follower cells using single-cell tracking to assess coordination of migration paths. We also measured cell velocity. Across the dataset, we observed a negative correlation between local cell packing density and migration velocity (Pearson's $\rho = -0.48$, $p = 2.2 \times 10^{-9}$), independent of the targeted gene (see figure for reviewer, left panel). This finding is consistent with previous work showing that endothelial cell coordination increases within the range of 200–800 cells/mm² (Hayer et al., 2016). Importantly, directionality did not scale with cell packing density within this range (see figure for reviewer, right panel), indicating that confluence was sufficient to support collective behavior across conditions in

the scratch assays. Thus, significant differences in directionality between knockdown groups reflect the effect on collective migration.

Because single cell migration velocity scaled with cell density and minor differences in packing density were present across conditions, velocity measurements were excluded from the final comparative analysis to avoid density-driven bias.

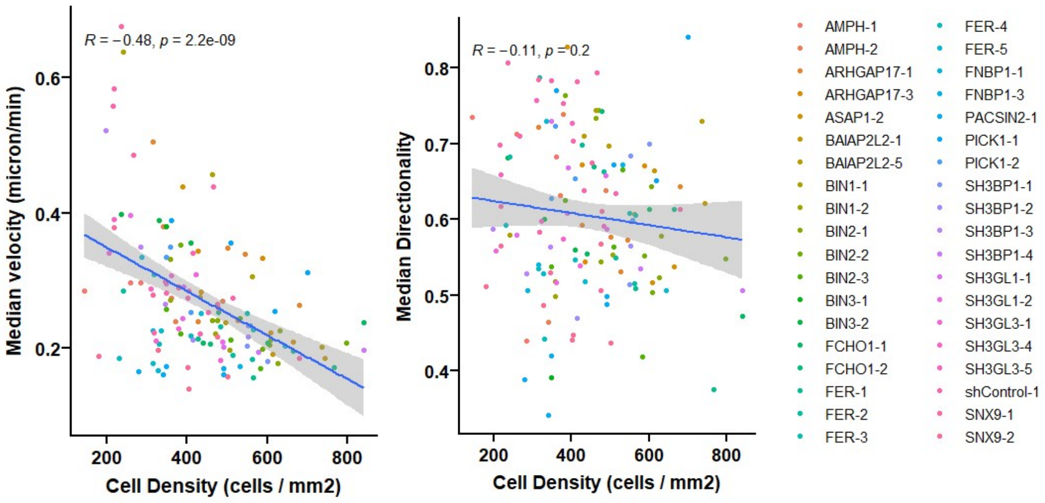

• Polarity readout versus junction state. The statement that unchanged Golgi polarity implies migration independent of junction-driven behavior may read a bit strongly given the Suppl. Fig. 4 examples (e.g., thinner junctions with shBIN3, reduced cytosolic VE-cadherin with shEndophilin-A2 in the shown ROI). Complementing the polarity metric with a basic junction intensity/morphometric readout - or adding a line that some candidates may still influence AAJs despite a negative polarity endpoint - could reduce the chance that readers interpret these as negatives.

The reviewer is correct. To prevent overinterpretation we have deleted the statement "suggesting that these proteins contributed to cell migration independently of junction-driven collective behavior".

We further added a discussion section to discuss this as follows: "However, not all junctional-localizing BAR proteins contributed equally to migration: for instance the knockdown of ASAP1 and PICK1, produced no detectable migration defect, while the depletion of Endophilin-A2 and BIN3 delayed wound closure, without affecting follower's cell polarity. Because several BAR proteins localize to similar regions at endothelial junctions, the loss of one protein may be compensated by others or by increased expression of related family members. As a result, the effects of individual knockdowns may underestimate the overall role of BAR proteins in junctional remodeling and endothelial migration. Future studies using combined genetic perturbations or acute protein degradation approaches will therefore be informative to define their collective functions."

Spatiotemporal recruitment of PACSIN2, BIN1 and SNX9 during junction remodelling (Fig. 4)

• The image analysis is excellent and clearly lays out time-resolved recruitment to AAJs.

Thank you!

Minor

• Because PACSIN2's sub-junctional pattern mirrors the curvature landscape in Fig. 1, a correlative cryo-SIM/FIB-SEM snapshot for BIN1 and SNX9 (even a single well-chosen example per protein) could nicely reinforce spatial separation narrative. If additional cryo-data aren't feasible, a brief note on how the ROIs in Fig. 4 map onto the curvature bins in Fig. 1 would also help readers connect the two datasets.

We thank the reviewer for this insightful suggestion. We agree that directly correlating the spatial distributions of BIN1 and SNX9 with the curvature landscape would further strengthen the interpretation. However, generating the original dataset for Fig. 1, including acquisition, processing, and curvature registrations of the FIB-SEM data, already required substantial effort over an extended period. As such, acquiring additional correlative cryo-SIM/FIB-SEM datasets is unfortunately not feasible within the scope of the current study.

Our FIB-SEM analysis shows that AAJs encompass a broad spectrum of membrane curvatures at the subjunctional level (~12–500 nm). The enrichment of PACSIN2 in these regions is consistent with its known role in sensing and/or inducing membrane curvature within this range. Given that BIN1 and SNX9 are also BAR domain-containing proteins, their subjunctional localization may reflect interactions with membranes spanning this curvature spectrum. However, this relationship remains qualitative, as direct spatial registration between datasets is not feasible (see also our answer to comment 6/reviewer 2).

SNX9 associates with dynamin-2 during asymmetric junction remodeling (Fig. 5)

Major

• Mechanistic link to DNM2 . The independence from PACSIN/EHD4/MICAL-L1 is well shown, and the SNX9-DNM2 co-localization is convincing. To close the loop on mechanism, silencing DNM2 alongside SNX9 to observe whether the observed junctional, polarity, and migration defects are further exacerbated, would make the conclusion land more firmly.

We thank the reviewer for these important questions and have further investigated the functional relationship between SNX9 and dynamin-2. To determine whether dynamin-2 acts downstream in this process, we depleted dynamin-2 using two independent shRNA clones (**Fig. 6h, i**). The more efficient shRNA (shDNM2-2) resulted in loss of EC adhesion, showing its importance, but precluding functional analysis. The moderate depletion achieved by shDNM2-1 was sufficient to perturb junction turnover, resulting in increased AAJ length and prolonged lifetimes (**Fig. 6j-l and Video 12**). Next, we assessed the role of dynamin-2 in endothelial collective migration. Notably, shDNM2-1 ECs failed to orient the Golgi toward the scratch wound (**Fig. 6m; Supplementary Fig. 4f**), however, these cells still migrated randomly and closed the wounds within a comparable timeframe to shControls (**Fig. 6n;**

Supplementary Fig. 4g and Video 13). Combined shSNX9 and shDNM2 knockdown did not further exacerbate the migration defect relative to shSNX9 alone (**Fig. 6n; Supplementary Fig. 4g and Video 13**). Together, these findings show that SNX9 regulates VE-cadherin turnover at AAJs, an effect that may be partially mediated through the recruitment of dynamin-2.

Minor

• Cell type context. BOECs and HUVECs appear interchangeably across experiments. A short rationale (e.g., availability, expression profile, or imaging suitability) and a line confirming that the key trends are consistent across the two would reduce perceived variability when comparing Fig. 4 and Fig. 5.

Our apologies for not explaining the choice of endothelial cells. BOECs and HUVECs are comparable in terms of junction organization. A benefit of BOECs is that these cells can be cultured for longer passages, which facilitates the generation and selection of lentiviral transduced fluorescent cells. Both BOECs and HUVEC are used interchangeably across the manuscript, which also validates findings across different cell sources.

SNX9 controls collective migration in the zebrafish CCV (Fig. 7)

Major

• Staging and controls. It would be helpful to know exactly when morphology was assessed in uninjected vs crispants, and whether any subtle developmental delay was observed. Given that the CCV phenotype is relatively modest, a brief control gRNA cohort and/or a snx9a/b rescue (even on a smaller subset) would be reassuring. If these additions aren't practical, a clear note on limitations and staging checks would still strengthen interpretation.

We thank the reviewer for this comment. For the morphology assessment, we staged the embryos at three moments. The first staging occurs 3 hours after injections when sorting the embryos at the same developmental stage, the second when selecting the GFP-positive embryos for images we ensured to check the vasculatures were developing at the same rate by looking at the ISVs and initiation point of the CCVs. Lastly, when analyzing the CCVs we assessed the growth rates between CCVs for the lengths across time for the individual conditions and observed a wider variation of 'starting' heights of the CCVs which was not crispant-specific. We then aligned all CCVs across the different experiments and timepoints to the growth rate of the uninjected controls and selected the maximum length of the CCVs that were fully visible at 40 hpf. The temporal alignment based on CCV length we have now noted in the 'shape analysis' and 'CCV analysis and cell tracking' section in the methods, and 'zebrafish experiments' section. Furthermore, after imaging experiments, heart beat and blood flow was assessed to ensure embryo viability was not affected by long-term imaging experiments.

Minor

• In vitro alignment. The endpoint data from the scratch assays (Supp. 4c) could be lightly re-analyzed with the same junction straightness/bifurcation descriptors used in the live AAJ analyses. Even a small concordance plot (e.g., straightness vs condition, focusing on shSNX9) would help tie the in vitro and in vivo readouts together.

For these overview images a lower magnification objective was used and the resulting resolution would not allow for an informative level of junction analysis.

Discussion

• Endothelial heterogeneity & curvature context. While the manuscript nicely emphasizes membrane composition and curvature preferences, a brief nod to heterogeneous expression across EC subtypes (e.g., arterial vs venous beds) and the possibility that curvature dynamics differ by vascular context could help frame generalizability. Even if outside the present scope, flagging this as a promising follow-up (e.g., profiling in defined arterial/venous settings or subtype-matched lines) would guide readers on where this could go next.

See our earlier response to this suggestion and see new Supplementary Fig. 3b for analysis of hierarchically clustered expression levels of genes encoding human BAR domain proteins across arterial, venous, capillary and lymphatic endothelial cell types in scRNA-seq data. Based on this, we have included in the Discussion section the following statement: "In this regard, the notion that BIN1 and other BAR proteins, such as SRGAP3, are highly expressed in lymphatic ECs (Supplementary Fig. 3b), indicate that differential BAR protein expression may finely tune junctional dynamics across different vascular beds."

• Redundancy and genetic compensation. The relatively modest or absent phenotypes for some candidates may reflect functional overlap. It could be helpful to underline this as a limitation and point to combinatorial perturbations or inducible degradation as a natural next step to map the machinery more completely. Framing it this way keeps the current results intact while signaling realistic routes to sharpen mechanism (e.g., pairwise KOs or acute degron tools to reduce compensation)

We agree that this is an important aspect to consider and have now added the following statements to the Discussion: " *Because several BAR proteins localize to similar regions at endothelial junctions, the loss of one protein may be compensated by others or by increased expression of related family members. As a result, the effects of individual knockdowns may underestimate the overall role of BAR proteins in junctional remodeling and endothelial migration. Future studies using combined genetic perturbations or acute protein degradation approaches will therefore be informative to define their collective functions*."

Methods

• For qPCR, a few extra lines could make replication easier: qPCR chemistry (dye vs probe), primer sequences/efficiencies and melt-curve checks, reference gene choice and ΔΔCt details (including how shControl baselines were handled), n values and outlier handling

We thank the reviewers for this important point regarding qPCR reporting and reproducibility. The qPCR data in question were derived from samples collected during the scratch wound assays presented in Figure 3. Due to the use of primary HUVECs following lentiviral transduction, the available cellular material for downstream analyses was limited. As a result, qPCR measurements could only be performed for a single independent experiment, which does not allow for meaningful statistical analysis. To avoid potential overinterpretation, we have removed the corresponding qPCR panel from the supplemental data. Instead knockdown efficiency for the shRNA constructs used in the scratch migration assays was independently validated at the protein level. As mentioned these blots serve as a qualitative validation of knockdown efficiency.

Minor comments

1. Figure formatting. Slight alignment/size tweaks across subpanels could make the multi-panel figures read more cleanly.

Thank you for mentioning this, we have aligned figure panels across the figures for improved presentation.

2. Nomenclature. Unifying Endophilin-A2 vs SH3GL1 across text and figures would avoid confusion.

The *SH3GL1* gene encodes the protein Endophilin-A2 (emphasized more clearly in the text now). In the revised manuscript we followed JCB's guidelines by referring to *SH3GL1* for gene-related contexts and use Endophilin-A2 for protein-related contexts.

3. Live imaging videos. Simple arrow/ROI labels (mirroring the figures) could guide viewers to the key events without rewatching.

To improve clarity, we have revised supplemental videos 1, 7, 8 and 10 by using Zooms and adding coloured arrows emphasizing AAJ remodeling and/or BAR protein recruitment.

June 1, 2026

RE: JCB Manuscript #202509207R

Stephan Huveneers
Amsterdam University Medical Centers

Dear Dr. Huveneers:

Thank you for submitting your revised manuscript entitled "Nanoscale junctional membrane curvatures recruit BIN1 and SNX9 for endothelial collective migration". The reviewers now support publication so we would be happy to publish your paper in JCB pending final revisions necessary to meet our formatting guidelines (see details below).

To avoid unnecessary delays in the acceptance and publication of your paper, please read the following information carefully.

A. MANUSCRIPT ORGANIZATION AND FORMATTING:

Full guidelines are available on our Instructions for Authors page, http://jcb.rupress.org/submission-guidelines#revised.
Submission of a paper that does not conform to JCB guidelines will delay the acceptance of your manuscript.

1) Text limits: Character count for Articles is < 40,000, not including spaces. Count includes abstract, introduction, results, discussion, and acknowledgments. Count does not include title page, figure legends, materials and methods, references, tables, or supplemental legends.

2) Figures limits: Articles may have up to 10 main text figures.

3) Figure formatting: Scale bars must be present on all microscopy images, including inset magnifications. Molecular weight or nucleic acid size markers must be included on all gel electrophoresis. Aspect ratios of images may not be altered.

4) Statistical analysis: Error bars on graphic representations of numerical data must be clearly described in the figure legend. The number of independent data points (n) represented in a graph must be indicated in the legend. Statistical methods should be explained in full in the materials and methods. For figures presenting pooled data the statistical measure should be defined in the figure legends. Please also be sure to indicate the statistical tests used in each of your experiments (either in the figure legend itself or in a separate methods section) as well as the parameters of the test (for example, if you ran a t-test, please indicate if it was one- or two-sided, etc.). Also, if you used parametric tests, please indicate if the data distribution was tested for normality (and if so, how). If not, you must state something to the effect that "Data distribution was assumed to be normal but this was not formally tested."

**5) Abstract and title: The abstract should be no longer than 160 words and should communicate the significance of the paper for a general audience. The title should be less than 100 characters including spaces. Make the title concise but accessible to a general readership.

Given that your abstract slightly exceeds the 160 word limit, we kindly ask you to shorten it a bit.

**6) Materials and methods: Should be comprehensive and not simply reference a previous publication for details on how an experiment was performed. Please provide full descriptions in the text for readers who may not have access to referenced manuscripts. For example, in the "DNA plasmids, lentiviral transduction and transfections" paragraph of your methods section, you state: "Lentiviral particles were produced as described previously (Malinova et al., 2021)." You must remove this and write out the full methodology. The same goes for all other places in the methods where you refer to methods "...as described previously."

7) All antibodies, cell lines, animals, and tools used in the manuscript should be described in full, including accession numbers for materials available in a public repository such as the Resource Identification Portal. Please be sure to provide the sequences for all of your primers/oligos and RNAi constructs in the materials and methods. You must also indicate in the methods the source, species, and catalog numbers (where appropriate) for all of your antibodies. Please also indicate the acquisition and quantification methods for immunoblotting/western blots.

8) Microscope image acquisition: The following information must be provided about the acquisition and processing of images:
a. Make and model of microscope
b. Type, magnification, and numerical aperture of the objective lenses
c. Temperature
d. Imaging medium

e. Fluorochromes
f. Camera make and model
g. Acquisition software
h. Any software used for image processing subsequent to data acquisition. Please include details and types of operations involved (e.g., type of deconvolution, 3D reconstitutions, surface or volume rendering, gamma adjustments, etc.).

9) References: There is no limit to the number of references cited in a manuscript. References should be cited parenthetically in the text by author and year of publication. Abbreviate the names of journals according to PubMed.

10) Supplemental materials: There are strict limits on the allowable amount of supplemental data. Articles may have up to 5 supplemental figures. Please also note that tables, like figures, should be provided as individual, editable files. A summary of all supplemental material should appear at the end of the Materials and methods section.

11) eTOC summary: A ~40-50-word summary that describes the context and significance of the findings for a general readership should be included on the title page. The statement should be written in the present tense and refer to the work in the third person.

12) Conflict of interest statement: JCB requires inclusion of a statement in the acknowledgements regarding competing financial interests. If no competing financial interests exist, please include the following statement: "The authors declare no competing financial interests." If competing interests are declared, please follow your statement of these competing interests with the following statement: "The authors declare no further competing financial interests."

**13) ORCID IDs: ORCID IDs are unique identifiers allowing researchers to create a record of their various scholarly contributions in a single place. Please note that ORCID IDs are now *required* for all authors. At resubmission of your final files, please be sure to provide your ORCID ID and those of all co-authors.

14) A separate author contribution section following the Acknowledgments. All authors should be mentioned and designated by their full names. We encourage use of the CRediT nomenclature.

Please note that JCB now requires authors to submit Source Data used to generate figures containing gels and Western blots with all revised manuscripts. This Source Data consists of fully uncropped and unprocessed images for each gel/blot displayed in the main and supplemental figures. For assays performed using capillary electrophoresis and/or immunoassay-based detection, authors should instead provide the electropherogram graph(s) for each experiment, plotting fluorescence/chemiluminescence intensity vs. molecular weight/size. Please be sure to provide one Source Data file for each figure gels, blots, and/or capillary electrophoresis assays along with your revised manuscript files. File names for Source Data figures should be alphanumeric without any spaces or special characters (i.e., SourceDataF#, where F# refers to the associated main figure number or SourceDataFS# for those associated with Supplementary figures). For traditional gels and blots, the lanes of the gels/blots should be labeled as they are in the associated figure, the place where cropping was applied should be marked (with a box), and molecular weight/size standards should be labeled wherever possible. For capillary electrophoresis assays, each trace in the graph should be color-coded and labeled to indicate which protein, gene, or sample is being measured (please try to avoid red/green combinations to accommodate our color-blind readers).

Source Data files will be made available to reviewers during evaluation of revised manuscripts and, if your paper is eventually published in JCB, the files will be directly linked to specific figures in the published article.

Source Data Figures should be provided as individual PDF files (one file per figure). Authors should endeavor to retain a minimum resolution of 300 dpi or pixels per inch. Please review our instructions for export from Photoshop, Illustrator, and PowerPoint here: https://rupress.org/jcb/pages/submission-guidelines#revised

Journal of Cell Biology now requires a data availability statement for all research article submissions. These statements will be published in the article directly above the Acknowledgments. The statement should address all data underlying the research presented in the manuscript. Please visit the JCB instructions for authors for guidelines and examples of statements at (https://rupress.org/jcb/pages/editorial-policies#data-availability-statement).

B. FINAL FILES:

Please upload the following materials to our online submission system. These items are required prior to acceptance. If you have any questions, contact JCB's Managing Editor, Lindsey Hollander (lhollander@rockefeller.edu).

-- An editable version of the final text (.DOC or .DOCX) is needed for copyediting (no PDFs).

-- High-resolution figure and MP4 video files: See our detailed guidelines for preparing your production-ready images, https://jcb.rupress.org/fig-vid-guidelines.

-- Cover images: If you have any striking images related to this story, we would be happy to consider them for inclusion on the journal cover. Submitted images may also be chosen for highlighting on the journal table of contents or JCB homepage carousel. Images should be uploaded as TIFF or EPS files and must be at least 300 dpi resolution.

It is JCB policy that if requested, original data images must be made available to the editors. Failure to provide original images upon request will result in unavoidable delays in publication. Please ensure that you have access to all original data images prior to final submission.

The license to publish form must be signed before your manuscript can be sent to production. A link to the license to publish form will be sent to the corresponding author only. Please take a moment to check your funder requirements before choosing the appropriate license.

Additionally, JCB encourages authors to submit a short video summary of their work. These videos are intended to convey the main messages of the study to a non-specialist, scientific audience. Think of them as an extended version of your abstract, or a short poster presentation. We encourage first authors to present the results to increase their visibility. The videos will be shared on social media to promote your work. For more detailed guidelines and tips on preparing your video, please visit https://rupress.org/jcb/pages/submission-guidelines#videoSummaries.

Thank you for your attention to these final processing requirements. Please revise and format the manuscript and upload materials within 14 days. If you need an extension for whatever reason, please let us know and we can work with you to determine a suitable revision period.

Please contact the journal office with any questions at cellbio@rockefeller.edu.

Thank you for this interesting contribution, we look forward to publishing your paper in Journal of Cell Biology.

Sincerely,

Sandrine Etienne-Manneville
Monitoring Editor
Journal of Cell Biology

Gabriele Stephan
Scientific Editor
Journal of Cell Biology

Reviewer #1:

The authors satisfactorily addressed my comments.

Reviewer #2:

I thank the authors for their careful and thoughtful responses. I especially appreciated the detailed discussions of membrane curvature in leader and follower cells. The authors have addressed all my concerns, and I find this work to be an important contribution, with cutting-edge multi-scale imaging providing significant insight and the basis for mechanistic advances.
